# Exploring treatment options in cancer: tumor treatment strategies

**DOI:** 10.1038/s41392-024-01856-7

**Published:** 2024-07-17

**Authors:** Beilei Liu, Hongyu Zhou, Licheng Tan, Kin To Hugo Siu, Xin-Yuan Guan

**Affiliations:** 1 Department of Clinical Oncology, The University of Hong Kong-Shenzhen Hospital, Shenzhen, China; 2 Department of Clinical Oncology, The University of Hong Kong, Hong Kong, China; 3 State Key Laboratory for Liver Research, The University of Hong Kong, Hong Kong, China; 4 Advanced Energy Science and Technology Guangdong Laboratory, Huizhou, China; 5 MOE Key Laboratory of Tumor Molecular Biology, Jinan University, Guangzhou, China

**Keywords:** Drug development, Drug development

## Abstract

Traditional therapeutic approaches such as chemotherapy and radiation therapy have burdened cancer patients with onerous physical and psychological challenges. Encouragingly, the landscape of tumor treatment has undergone a comprehensive and remarkable transformation. Emerging as fervently pursued modalities are small molecule targeted agents, antibody-drug conjugates (ADCs), cell-based therapies, and gene therapy. These cutting-edge treatment modalities not only afford personalized and precise tumor targeting, but also provide patients with enhanced therapeutic comfort and the potential to impede disease progression. Nonetheless, it is acknowledged that these therapeutic strategies still harbour untapped potential for further advancement. Gaining a comprehensive understanding of the merits and limitations of these treatment modalities holds the promise of offering novel perspectives for clinical practice and foundational research endeavours. In this review, we discussed the different treatment modalities, including small molecule targeted drugs, peptide drugs, antibody drugs, cell therapy, and gene therapy. It will provide a detailed explanation of each method, addressing their status of development, clinical challenges, and potential solutions. The aim is to assist clinicians and researchers in gaining a deeper understanding of these diverse treatment options, enabling them to carry out effective treatment and advance their research more efficiently.

## Introduction

Cancer has become a crucial public health challenge. Daily, over 52,900 individuals are diagnosed with cancer, and more than 27,000 people lose their lives to this disease.^1^ It is estimated that by 2040, there will be 28 million new cases and 16.2 million deaths worldwide.^2^ The best strategy for continuously reducing global cancer mortality is the widespread implementation of precise and individualized treatment and increased investment in advancing cancer drug research. The cancer treatment timeline documents the evolution of therapies over the past 170 years, highlighting the transformative treatments that have emerged to enhance clinical outcomes and improve patients’ quality of life. Starting from the early use of general anesthesia in surgical resections in the mid-1800s, to Wilhelm Conrad Röntgen’s invention of X-rays at the end of the 19th century,^3^ which initiated the era of combining radiation with surgery for cancer treatment, to the breakthroughs in chemotherapy during World War II (WWII),^4^ and the recent advancements in immunotherapy and gene therapy, each milestone has played a pivotal role in the ongoing fight against cancer.

**Fig. 1:**
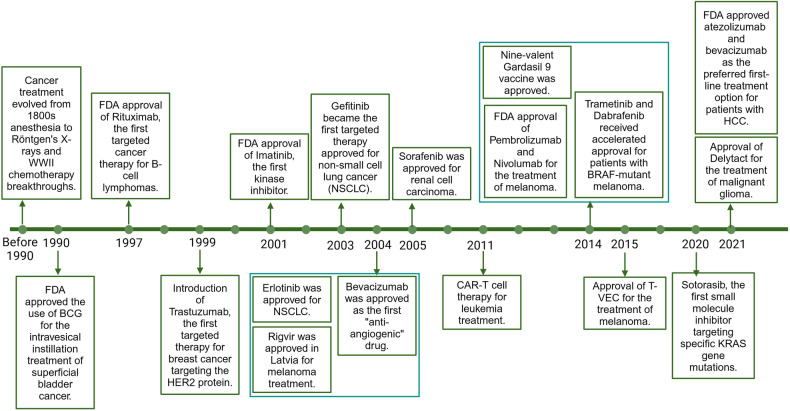
**The milestone of cancer therapy development**. This timeline illustrates the significant advancements in cancer therapy over the past 170 year. Beginning with the adoption of general anesthesia for surgical procedures in the mid-1800s and the groundbreaking invention of X-rays by Wilhelm Conrad Röntgen in the late 19th century, which paved the way for radiation therapy combined with surgery, the field of oncology has witnessed a series of transformative treatments.Key developments include the introduction of chemotherapy during World War II, the advent of immunotherapy, and the more recent progress in gene therapy. The 1990s marked a turning point with the FDA approval of BCG for bladder cancer treatment and Rituximab for B-cell lymphomas, initiating the era of targeted therapies. Trastuzumab and Imatinib further revolutionized the treatment of breast cancer and chronic myeloid leukemia, respectively. The new millennium brought targeted therapies for non-small cell lung cancer with drugs like Gefitinib and Erlotinib, and the first anti-angiogenic drug, Bevacizumab, which targeted tumor blood supply. Rigvir’s approval in Latvia for melanoma treatment signified the global reach of cancer advancements. The successful application of CAR-T cell therapy by Carl June in 2011 and the FDA approval of Pembrolizumab and Nivolumab in 2014 for melanoma treatment highlighted the potential of immunotherapy. Preventive measures also evolved, as evidenced by the approval of the nine-valent Gardasil 9 vaccine, offering broader protection against HPV strains linked to cervical cancer. The resurgence of oncolytic viruses was evident with the approval of T-VEC and Delytact for melanoma and malignant glioma, respectively. The 2020 s have introduced targeted therapies for KRAS gene mutations with Sotorasib and the combination of atezolizumab and bevacizumab as a preferred first-line treatment for hepatocellular carcinoma, as endorsed by the NCCN guidelines in 2021. This figure encapsulates the continuous innovation and dedication to enhancing cancer care, reflecting the dynamic nature of the fight against cancer and the pursuit of improved patient outcomes. This figure was created with Biorender.com

In 1990, the U.S. Food and Drug Administration (FDA) approved the use of BCG for the intravesical instillation treatment of superficial bladder cancer.^5^ In 1997, the FDA approved Rituximab, the first targeted therapy for B-cell lymphomas,^6^ marking the beginning of a new era of targeted treatments. Two years later, Trastuzumab was introduced, becoming the first targeted therapy for breast cancer by targeting the HER2 protein,^7^ significantly impacting treatment strategies. The year 2001 saw another milestone with the FDA approval of Imatinib,^8^ the first kinase inhibitor, which revolutionized the treatment of chronic myeloid leukemia and other rare gastrointestinal tumors. In 2003, Gefitinib became the first targeted therapy approved for non-small cell lung cancer (NSCLC),^9^ followed by Erlotinib in 2004,^10^ expanding the options for NSCLC patients. Also in 2004, Bevacizumab^11^ was approved as the first “anti-angiogenic” drug, demonstrating a new approach to cancer therapy by targeting the blood supply to tumors. That same year, Rigvir^12^ was approved in Latvia for melanoma treatment. Subsequent years saw a steady stream of targeted drugs, with Sorafenib^13^ in 2005 for renal cell carcinoma. In 2011, Carl June’s successful application of CAR-T cell therapy for leukemia treatment marked a significant step forward in immunotherapy.^14^ The 2014 FDA approval of Pembrolizumab and Nivolumab for the treatment of melanoma, along with the accelerated approval of Trametinib and Dabrafenib for patients with BRAF-mutant melanoma, marked a new beginning in cancer immunotherapy.^15–17^ Preventive measures also advanced, with the approval of the nine-valent Gardasil 9 vaccine in December 2014,^18^ offering broader protection against HPV strains associated with cervical cancer. The approval of T-VEC in 2015 and Delytact in 2021 for melanoma and malignant glioma, respectively, highlighted the resurgence of oncolytic viruses as a cancer treatment modality.^19,20^ The 2020 s have seen further advancements with the FDA approval of Sotorasib, the first small molecule inhibitor targeting specific KRAS gene mutations.^21^ In 2021, the National Comprehensive Cancer Network (NCCN) guidelines highlighted the combination of atezolizumab and bevacizumab as the preferred first-line treatment option for patients with hepatocellular carcinoma (HCC).^22^ This recommendation underscores the importance of immunotherapy and anti-angiogenic therapy in the frontline management of this aggressive form of cancer, reflecting the evolving landscape of cancer care and the continuous efforts to improve patient outcomes (Fig. 1). As of December 2021, there were 107 operational proton and heavy ion therapy centers worldwide, including 12 carbon ion therapy centers.^23^

Now, the field of oncology is experiencing a proliferation of tumor drugs, leading to a flourishing phase in the oncology drug market. According to Frost & Sullivan, the global anti-tumor drugs market is expected to grow at a CAGR of 4.20% from 2022 to 2029, with an estimated market size of USD 94,340 million in 2022 and projected to reach USD 125,825.86 million by 2029.^24^ With the thriving development of tumor basic research in cell targets, signaling pathways, immune escape, etc., there are several trends in the development of tumor drugs. Firstly, the functions of tumor drugs are becoming increasingly specialized. Tumor drugs are no longer limited to chemotherapy-based tumor killing, such as Alkylating agents (e.g., Mechlorethamine, Cyclophosphamide),^25^ but also include differentiation in angiogenesis inhibition (e.g., EGFR inhibitors like Lapatinib and Gefitinib and VEGFR inhibitors e.g., Sunitinib, Sorafenib),^25^ tumor metabolism regulation (e.g., IDO1 inhibitors, IDH1 and IDH2 inhibitors),^26^ and restoration of self-immunity (e.g., anti-CD20 antibodies (rituximab and obinutuzumab).^27^ The combination of various drugs makes tumor therapy more precise and effective. Secondly, there is a gradual shift in tumor drugs from small molecules to large molecules. The evolution includes small molecule inhibitors (e.g., Imatinib, Lapatinib, and Neratinib), peptides (including Sandostatin, Lutathera, Kyprolis, and Zoladex), antibody drugs (e.g., trastuzumab deruxtecan [Enhertu] and Trastuzumab emtansine [T-DM1]), and cell therapies (comprising various chimeric antigen receptor [CAR]-T cells, NK cells, macrophages, and tumor-infiltrating lymphocyte [TIL] therapy). The design and preparation of tumor drugs have become more complex, and the preparation methods have advanced. Thirdly, tumor drugs are shifting from inhibiting tumor cell functions (e.g., tumor neoantigen suppression, surface receptor inhibition) to regulating self-immune activation, such as with Antibody-Drug Conjugates (ADCs). Fourthly, the treatment landscape has expanded from monotherapy to combination therapies, encompassing a variety of immunomodulators, anti-angiogenic drugs, chemotherapies, and targeted therapies. Lastly, a diverse array of treatments, including previously ‘undruggable’ targets, peptide drugs, monoclonal antibodies, ADCs, cell therapies, gene therapy, neoantigen and cancer vaccines, oncolytic viruses, immunologic adjuvants, innate immunity activators, proton therapy, carbon ion therapy, photothermal and photodynamic therapy, and anti-angiogenesis therapy, is reshaping the old cancer drug market and ushering in a more diversified era of tumor treatment.

In this review, we will introduce different types of anti-tumor drugs based on the principles of drug classification. We will analyze these different anti-tumor drug strategies from the perspective of their mechanisms of action, development history, basic design principles, advantages, disadvantages, and current existing bottlenecks. We aim to contribute to the translation of basic scientific research into clinical drug selection.

## Small molecule inhibitors

Small molecule inhibitors can interfere with or block the activity of specific molecules by interacting with them. These molecules are typically proteins, which play important roles in cell signaling, gene expression, and metabolism. By binding to target molecules, small molecule inhibitors can disrupt their normal function, thereby interfering with disease progression or treating certain conditions.

### Artificial intelligence and cryo-EM for protein structure analysis

Computational modeling has become an important tool in small molecule drug discovery, enabling faster and more successful identification of drug candidates. Computational methods are applied at three main stages of small molecule drug discovery, including the initial identification of active substances (i.e. lead compound discovery) through large-scale exploration of chemical space and the use of molecular docking, hit-to-lead compound selection using machine learning and physics-based methods to refine lead compounds, and multi-parameter optimization of lead compounds using physics-based, structure-based, QSAR, and machine learning methods to achieve the desired target product features.^28^ These methods allow for a balance of potency, selectivity, and ADMET properties to support the efficacy relationship required for in vivo pharmacokinetics/pharmacodynamics under tolerable exposure. By integrating computational modeling with experimental validation, more efficient and successful drug discovery can be achieved.

In the step of initial identification of active substances, structure biology is crucial for small molecular inhibitor discovery. With the availability of atomic resolution data on the active or regulatory sites of proteins, the design of drug structures becomes feasible. At present, three main techniques are used in structural biology research: X-ray crystallography, nuclear magnetic resonance (NMR), and cryo-EM.^29^ X-ray crystallography reveals atomic details for small, crystalline complexes up to 150 kD but is limited for larger or membrane proteins.^30^ NMR can analyze smaller proteins (up to 50 kD) in solution without crystals, needing isotope labeling.^31^ Cryo-EM determines structures of large complexes and membrane proteins without crystallization, capturing various conformations for easier structural analysis, which is highly convenient for elucidating structures.

Due to structural analysis using cryo-EM of biologically important macromolecules becomes increasingly complex, Artificial intelligence (AI) and cryo-electron microscopy (cryo-EM) have emerged as powerful tools to solve this problem. The current workflow for cryo-EM combined with AI includes particle identification, three-dimensional reconstruction, resolution enhancement, automated high-throughput analysis, pattern recognition, drug design assistance, and data interpretation as well as hypothesis generation. Since cryo-EM AI processes biological proteins while maintaining their bioactivity, a lower electron dose is used to reduce radiation damage.^32–34^ Noisy images with low contrast were generated, making it difficult to discern details from the raw micrographs. With machine learning models, such as DeepPicker,^35^ DeepEM,^36^ and convolutional neural networks (CNNs), AI are trained to recognize and classify different types of particles. Through deep learning of two-dimensional image data, feature extraction, and classification of key information, AI can construct initial models for the targeted proteins. Further iterative optimization, parameter adjustments—such as particle orientation, position, and scaling—and resolution enhancement lead to the final acquisition of high-resolution 3D protein models suitable for research and drug development. Based on the established models, AI can perform high-throughput data analysis, decipher protein conformations, calculate molecular binding affinities, predict, and explore interactions between small molecules and proteins, and assist in discovering and optimizing potential drug candidates. Finally, by predicting the function of drugs and the generated protein structures, the inhibitory efficacy of small molecule drugs on proteins is evaluated. The application of AI in cryo-EM has significantly increased the speed and quality of data analysis, accelerating the entire process from raw data to final structural determination, and bringing revolutionary changes to the fields of structural biology and drug discovery.

### The advantage and disadvantage of small molecular inhibitors

As of 2023, the US FDA has approved 72 small molecule therapeutic protein kinase inhibitors for cancer.^37^ There are 57 anti-solid tumor drugs.^37^ For example, Imatinib (Glivec), as the first small molecular inhibitor specifically designed to address the mechanisms of tumor formation, has heralded a new epoch in cancer therapy with its successful development and application. Ithas been approved for the treatment of eight different diseases, including certain types of leukemia and gastrointestinal stromal tumors (GIST).^38^ Imatinib is a tyrosine kinase inhibitor that targets specific abnormal protein kinases involved in the growth and proliferation of cancer cells. Its approval for multiple indications highlights its efficacy and versatility in treating various types of solid tumors.^38^

Compared to other drugs, small molecule drugs have simpler and less expensive synthesis and preparation processes. They are often administered orally, which is more convenient for patients and has fewer side effects. However, small molecule drugs target proteins, typically enzymes or receptors, which may result in limited inhibitory effects on membrane proteins and secretory proteins. Additionally, due to the influence of metabolism, it is challenging to adjust the dosage of small molecule inhibitors to achieve optimal efficacy. In particular, the challenge of undruggable proteins which has hampered the design of drugs that target many oncogenes.

### Strategies for undruggable proteins

It has been reported that notable and infamous players in cancer initiation and progression, which are not treatable by conventional therapies, include transcription factors (such as p53, MYC, E2F, or Kruppel-like factor 4 (KLF4)), phosphatases (such as PP2δ, PP2A, or PTP1B), and the well-known RAS family. Despite being identified as the first human mutated cancer gene in 1982, the RAS family has remained undruggable.^39^ It wasn’t until 2013 that Shokat first reported the feasibility of using small molecule covalent binding to target the KRAS^G12C^ mutant (one of the most common RAS mutations in non-small cell lung cancer (NSCLC)).^40^ New inhibitors targeting the KRASG12C mutation, such as Adagrasib^41^ and Sotorasib,^42^ have shown clinical efficacy in patients with locally advanced or metastatic NSCLC, and the FDA has approved these drugs for the treatment of patients with KRAS^G12C^ mutated NSCLC. However, not all RAS mutations are G12C, and compounds targeting other KRAS subtypes beyond G12C are also in development, which are commonly found in NSCLC, pancreatic cancer, and colorectal cancer.^43^ Compared to RAS, other fusion transcription factors commonly seen with pediatric cancers have been deemed undruggable due to large protein–protein interaction (PPI) interfaces or their lack of deep protein pockets.^44^ Based on the various characteristics of existing undruggable proteins, current drug design strategies include covalent modulation, allosteric inhibition, Proteolysis-targeting chimeras (PROTAC) and Molecular Glue Degraders (MGDs).^45^

#### Covalent modulation

Covalent modulation refers to the binding of small molecule inhibitors to their targets through the formation of irreversible covalent bonds (i.e., chemical bonds) to alter their activity and function. The interaction between the drug molecule and the target is highly stable until the covalent bond is broken by specific biochemical processes, such as metabolism. This approach enables drug design for proteins that lack surface pockets. It is important to highlight the KRAS family of genes, which have been synonymous with undruggable targets. KRAS, as a widely acting gene, frequently undergoes mutations in various cancers. The G12C mutation, for instance, accounts for approximately 25% of mutations in non-small cell lung cancer.^46^ Normal KRAS protein functions as a GTPase, binding to GTP in its active state and switching back to an inactive state by binding to its hydrolysis product GDP after GTP hydrolysis.^47^ KRAS mutations (such as the G12C mutation) reduce the GTPase activity of the KRAS protein, causing it to persistently bind to GTP, thereby continuously activating downstream signaling pathways, such as MAPK/ERK and PI3K/AKT, promoting tumor proliferation.^48^ Due to the relatively smooth surface of the KRAS protein, which lacks obvious pockets, and its propensity to bind GTP, the development of drugs that competitively inhibit KRAS binding to GTP has been challenging. Moreover, the development of drugs targeting downstream signaling molecules such as RAF, MEK, ERK, and PI3K has also encountered significant difficulties.^49^ Research on KRAS-related inhibitors has been on hold. AMG 510 (Sotorasib), approved by the FDA in 2021, is the first small molecular inhibitor targeting specific KRAS gene mutations.^50^ It leverages the unique chemical properties of the cysteine (Cys) residue in the KRAS G12C mutant to covalently bind to this residue, locking KRAS G12C in an inactive state and preventing it from binding to GTP, thereby inhibiting KRAS-mediated downstream signaling pathways and suppressing tumor growth.^51^ The launch of AMG 510 signifies a milestone in the “undruggable” proteins’ history. Currently, drugs produced using covalent binding methods target both EGFR and P53. These include EGFR inhibitors Afatinib, Dacomitinib, and Osimertinib, as well as P53 inhibitor KG13. Clinical trials for other targets are also underway.

#### Allosteric inhibition

Allosteric inhibition refers to inhibitors bind to allosteric sites on proteins to change the protein’s conformation, thereby altering its biological function. Allosteric modulation is prevalent in various intricate cellular activities, such as Signal Transduction, Enzymatic Catalysis, Cellular Metabolism, and Gene Regulation.^52^ G protein-coupled receptors (GPCRs) and protein kinases are two large classes of molecules related to numerous cellular activities. For example, Asciminib^53^ (ABL001) is an allosteric inhibitor for chronic myeloid leukemia (CML) that locks BCR-ABL1 in an inactive conformation by binding to the myristoyl pocket (STAMP), overcoming drug-resistant mutations such as the T315I mutation. Cobimetinib is a MEK kinase inhibitor, used for treating melanoma with BRAF V600E or V600K mutations.^54^ SHP099 is an allosteric inhibitor that inhibits SHP2’s activity by binding to multiple structural domain interfaces of SHP2. In terms of design, allosteric modulator target high-entropy, low-conservation allosteric sites, and their significant variability determines that the corresponding allosteric drugs have higher selectivity.^55^ Unlike traditional inhibitors, which rely on competitive occupancy, these protein-protein interactions drugs bind with a transient nature, allowing low doses of the medication to achieve the desired effect and exhibiting greater resistance to drug resistance. However, allosteric inhibitors also face challenges. Firstly, allosteric inhibition is highly dependent on protein model analysis, thus requiring increased computational power and algorithmic improvements to assist in identifying allosteric sites. Secondly, drug-resistant mutations and species differences in animal models and human may cause changes in the binding of allosteric inhibitors to their sites, requiring further in-depth research to circumvent this issue.

#### PROTACs

PROTACs are a strategy that utilizes protein degradation mechanisms to eliminate target proteins by simultaneously connecting the target protein with an E3 ubiquitin ligase to form a ternary complex molecule for the degradation of the target protein. The development of anticancer PROTACs primarily uses ligands for E3 ligases such as CRBN, VHL, MDM2, IAPs, AhR, DCAF15, DCAF16, RNF4, and RNF114.^56–62^ These ligands are tumor-specific to prevent off-target toxicity of PROTACs. Currently, PROTACs have been developed for various targets to combat solid tumors and malignant hematological cancers, such as those targeting AR (Bavdegalutamide^63^ (also known as ARV-110), CC-94676,^64^ AC176,^65^ HP518,^66^ and ARV-766^67^), ER (ARV-471^68^ and AC682^69^), and BTK (NX-2127,^70^ NX-5948,^71^ BGB-16673,^72^ and HSK29116^73^). These drugs have entered clinical trial stages, with Bavdegalutamide (NCT03888612), ARV-471 (NCT04072952), and NX-2127 (NCT04830137) showing their therapeutic effectiveness; the PROTAC targeting MAP4K1 has the potential to be a “first-in-class” therapy mimicking PD-1/PD-L1 targeted therapy; while PROTACs targeting BCL-XL^74^ and ALK have shown broad-spectrum antitumor activity, effectively killing leukemia and solid tumor cells in both in vitro and in vivo preclinical experiments. Like allosteric inhibition, PROTACs also showed the advantages of low dose, safety and resistance to drug resistance. However, the current size of PROTACs exceeds 1000 Daltons, making tissue and cellular permeability remain as major challenges.

#### MGDs

MGDs are a class of small molecules that facilitate the interaction between target proteins and E3 ubiquitin ligases, leading to the ubiquitination and subsequent degradation of them. They offer potential advantages over PROTACs, such as improved pharmacokinetic properties, including better membrane permeability, cellular uptake, and blood-brain barrier penetration. Despite their promise, the development of MGDs is largely serendipitous and lacks systematic design approaches, necessitating further theoretical exploration to aid in the rational design of these drugs. Notably, the FDA has greenlit clinical trials for several molecular glue agents, such as MRT-2359 (NCT05546268), a GSPT1-targeting molecular glue for multiple solid tumors, SP-3164 (NCT05979857) for its anti-tumor activity in follicular lymphoma models with expected clinical trials in 2023, and IK-595 (NCT06270082) for advanced solid tumors with RAS-MAPK pathway alterations, set to begin in 2024.^75–77^

### Challenges

With the development of the aforementioned technologies, proteins that were once considered “undruggable” are gradually shedding that label and becoming “yet-to-be-drugged” proteins. The application of small molecule inhibitors has been further expanded. However, the future of small molecule inhibitors still faces many challenges. For instance, patients with EGFR mutations treated with first and second-generation EGFR tyrosine kinase inhibitors (such as Gefitinib, Erlotinib, Afatinib, and Dacomitinib) approximately have a 50–60% chance of developing resistance within one year of treatment.^78^ It is necessary to continue treating patients by replacing other therapeutic methods. Next is the challenge of designing small molecule inhibitors, including the need for further breakthroughs when dealing with highly homologous protein families and “undruggable” targets. Finally, there is the consideration of the safe dosage and metabolic stability of small molecule inhibitors in the body. It is necessary to ensure that small molecule inhibitors are safe and effective within their therapeutic window.

## Peptide drugs

Peptide drugs refer to specific therapeutic peptides synthesized chemically, through genetic recombination, or extraction, typically composed of 10–50 amino acids. During the exploratory period before the 1960s, significant advancements were made in peptide drug development. The successful extraction of insulin in 1921 marked a major milestone, leading to improved symptoms in diabetic patients.^79^ The rapid development period from 1960 to 2000 saw revolutionary advancements in peptide synthesis. The invention of solid-phase peptide synthesis (SPPS) by Robert Bruce Merrifield made synthesis more convenient and faster.^80^ The 1980s witnessed the emergence of recombinant technology and phage display technology, enabling the production of larger molecular weight peptide drugs and the screening of peptides with specific characteristics from large libraries.^81^ In the explosive period after 2000, the field of peptide drugs experienced significant growth. Natural peptides were enriched through techniques such as peptidomics from venom and new chemical modification methods.^82^ This facilitated the discovery of novel peptide drugs. Additionally, the emergence of novel technologies like multifunctional peptides, constrained peptides, conjugated peptides, oral peptides, long-acting formulations, and delivery systems further advanced the field.

### Peptide-based imaging and therapeutic approaches

In addition to their pharmaceutical applications, the high affinity of peptides for specific receptors has developed a specific application in the diagnosis and treatment. For example, peptide Scintigraphy and Peptide Receptor Radionuclide Therapy (PRRT) are two important techniques in neuroendocrine tumors (NETs). Peptide Scintigraphy, as a nuclear medicine imaging technique, uses peptides labeled with radioactive isotopes such as 111In (indium)^83^ and 68Ga (gallium),^84^ like 111In-octreotide and 68Ga-DOTATOC, to detect tumor cells. These radiopharmaceuticals circulate through the bloodstream to tumor cells that express specific peptide receptors and are imaged using Single Photon Emission Computed Tomography (SPECT) or Positron Emission Tomography (PET). Peptide Scintigraphy is very useful for assessing the staging, distribution, and treatment response of NETs. Peptide Receptor Radionuclide Therapy (PRRT) is a targeted therapy that combines the targeting ability of peptides with the cytotoxicity of radionuclides. Therapeutic peptides are labeled with radionuclides such as 90Y (yttrium)^85^ or 177Lu (lutetium),^86^ which emit beta particles that are lethal to tumor cells. PRRT is particularly effective for tumors that express somatostatin receptors (SSTRs). Additionally, technetium-99m labeled peptide GX1, which specifically binds to tumor vessels of gastric, colorectal, and glioma tumors, shows promise as a new tumor imaging biomarker.^87,88^ Copper-64 labeled PD-L1 affinity peptide WL12,^89^ with PET imaging results indicating that [64Cu] WL12 can be used as a radiotracer to specifically detect tumors expressing PD-L1, providing a basis for the development of tumor immunotherapy strategies.

### The advantage and disadvantage of peptide drugs

As of January 2023, there are approximately 180 peptide drugs on the market globally.^90^ International peptide drugs are mainly distributed in diabetes and cancer. In the list of the top 25 best-selling peptide drugs in 2022, four are anti-tumor medications: Sandostatin and Lutathera from Novartis, Kyprolis from Amgen, and Zoladex from AstraZeneca (Table 1).^90–92^ Compared to small molecule inhibitors, peptide drugs operate at lower concentrations and offer better effects. Classical therapeutic peptides, such as hormones, growth factors, and ion channel ligands, enhance the specificity of peptide cancer therapy by efficiently triggering intracellular effects through specific receptor binding. Compared to other large biomolecule formulations, peptides exhibit lower immunogenicity and are generally safer. They have good tissue penetration, are easily chemically synthesized, and modified, and are cost-effective to produce with high efficacy. However, the drawbacks of peptide drugs are quite evident. Compared to chemical drugs, peptide-based medications have unstable physicochemical properties, shorter half-lives, are easily cleared by the body, and most cannot be administered orally. Chemical modifications, such as acetylation and methylation of the N-terminus, protect peptides from recognition and clearance by proteases or peptidases. The use of synthetic or non-canonical amino acids, such as α-aminobutyric acid and β-amino acids, as well as the design of isomeric amino acid surrogates, are both helpful for increasing their stability. Notably, many peptide drugs are designed for targeting extracellular proteins due to their poor membrane permeability. To improve passive membrane permeability, chemical modifications like peptide cyclization, N-methylation, and the formation of intramolecular H-bonds, along with novel prodrug strategies, are introduced into peptide drug design. Additionally, peptides linked to ligands of membrane receptors can be increasingly absorbed via active membrane transport. Low oral bioactivity is also a primary obstacle for their therapeutic application. To avoid cleavage in the intestinal tract, combining peptides with several protease inhibitors, as well as with permeation enhancers such as citric acid and ethylenediaminetetraacetic acid (EDTA), can increase the efficiency of oral delivery. To decrease renal clearance and extend the half-life of peptides, the molecule’s size is typically enlarged through binding with plasma proteins, such as albumins.^93^

**Table 1:** Summary of tumor-related peptide drugs ranked in the global top 25 sales of peptide drugs in 2022

Product Name	Company	Disease	Approval Date	2022 sales revenue (in billions USD)
Sandostatin	Novartis	Tumour	1988	12.38
Zoladex	AstraZenca	Cancer	1989	9.27
Decapeptyl	Ipsen	Hormone-dependent prostate cancer	1986	5.57
Lutathera	Novartis	Gastrointestinal pancreatic neuroendocrine tumor	2017	4.71

## Monoclonal antibody therapy

Monoclonal antibodies (mAbs) are produced by B cells and specifically target antigens. There are five isotypes of mAbs: IgG, IgA, IgM, IgD, and IgE. Because of its extended half-life and high affinity, IgG, —particularly IgG1 and IgG4,isotypes are frequently used in the development of monoclonal antibodies. Since the introduction of the first monoclonal antibody drug, Rituximab, in 1997, immunoglobulins have been potent drugs for cancer treatment in recent decades.^94^ By 2023, the US FDA has approved 79 therapeutic monoclonal antibodies, of which at least 48 are used for cancer treatment, as summarized in Supplementary Table 1.^95^ These monoclonal antibodies are a class of proteins that target specific antigens to exert single or multiple effects for eliminating cancers.

### Ligands or receptors blockades

The anti-tumor effects of representative receptor blockades, such as trastuzumab (Herceptin) and cetuximab (Erbitux), are primarily achieved by directly interacting with the tumor surface receptors HER-2 and EGFR, respectively. This significantly inhibits the progression and migration of cancer, especially in cases of HER2+ breast cancer (Herceptin),^96^ metastatic colorectal cancer (Eribitux), and advanced head and neck cancer (Erbitux).^97^ Two additional HER-2-targeting monoclonal antibodies, margetuximab (Margenza) and pertuzumab (Perjeta), have been approved for the treatment of metastatic HER-2+ breast cancer, either in combination with or as an alternative to Herceptin.^98,99^

Ligand blockades, such as bevacizumab (Avastin), work by preventing VEGF-A from binding to its receptors, thereby suppressing angiogenesis and neovascularization. As a first-line anti-angiogenic therapy, Avastin has been approved for treating several types of cancer, including colorectal, lung, and ovarian malignancies.^11,100^

### Cytotoxic mAbs

This cytotoxic monoclonal antibody works by targeting antigens, a strategy frequently used in the treatment of hematological tumors. In 1997, the first mAb licensed for the treatment of B-cell lymphoma was rituximab, also known as Rituxan. It induces B cells to become cytotoxic and initiates caspase-independent programmed cell death by directly blocking the CD20 antigen on B lymphocytes. Rituximab-treated B-cell lymphomas have shown improved overall survival (OS) and progression-free survival (PFS). Compared to 56% for rituximab, the objective response rate for ibritumomab tiuxetan (Zevalin), a CD20-targeting radio-conjugate, was higher at 80%.^101^

Additionally, cytotoxic antibodies that directly against CD52 (alemtuzumab), CD47, HLA-DR, CD74, and CD99, without the need for caspase, also lead to the death of target cells. Notably, in multicenter clinical studies, the CD52-targeting monoclonal antibody alemtuzumab demonstrated an extended median OS (12–35.8 months), a median PFS (4.7–19.6 months), and an overall response rate (ORR)(31–54%).^102^ Oncology treatment involving monoclonal antibodies that target CD47, HLA-DR, CD74, and CD99 are still under investigated.

Besides, clinical trials are underway to investigate hhow novel cytotoxic mAbs activate apoptosis-related receptors, such as TNF-related apoptosis inducing-ligand (TRAIL) death receptors DR4 and DR5, leading to Fas- and caspase-dependent cell apoptosis.^103^

### Immune checkpoints blockades (ICIs)

In addition to directly inhibiting tumor antigens, it’s critical to modify anti-tumor immunity. Tumor cells can trick the immune system by abnormally activating inhibitory signals that weaken cytotoxic T cells, leading to a state of immune tolerance and exhaustion. This process has become a promising approach for developing new cancer therapies.

PD-1 (Programmed Death 1) and PD-L1 (Programmed Death-Ligand 1) ICIs are a type of cancer treatment that enhances the immune system’s ability to fight cancer by blocking the proteins cancer cells use to evade immune cells. PD-L1 is a protein that can be upregulated on cancer cells, interacting with PD-1 to effectively ‘instruct’ T cells to stand down and not attack the cancer cells. Notably, Pembrolizumab, Nivolumab, and Cemiplimab are representative anti-PD-1 agents,^104^ while Atezolizumab, Avelumab, and Durvalumab are examples of anti-PD-L1 agents.^105^ The use of these drugs has been associated with improved outcomes in non-small cell lung cancer (NSCLC), melanoma, renal cell carcinoma (RCC), and other cancers. This progress marks a significant breakthrough in cancer therapy due to the development of PD-1 and PD-L1 ICIs.

Cytotoxic T-lymphocyte-associated protein 4 (CTLA-4), highly expressed in T cells, is an immune inhibitory molecule that interacts with B7 family of ligands (CD80 and CD86) to induce its immunosuppressive effect. In cytotoxic T cells, CTLA-4 competitively shatters the bond between ligands and its co-receptor CD28, thereby destroying its anti-tumor function. Ipilimumab was approved as an anti-CTLA-4 monoclonal antibody (2011) for interrupting B27/CTLA-4 binding andrestoring T lymphocytes cytotoxicity.^106^ In a HIMALAYA clinical trial, tremelimumab (Imjudo), a CTLA4-targeting monoclonal antibody, coupled with durvalumab led to a longer OS (16.43 months) for liver cancer patients compared to 13.8 months for the sorafenib-treated group.^107^

T cell immunoglobulin and mucin-domain-containing 3 (TIM-3), lymphocyte activation gene-3 (LAG-3), and T cell immunoglobulin and ITIM domain (TIGIT) represent additional significant immune checkpoint inhibitors (ICIs) for monoclonal antibodies (mAbs). TIM-3, a transmembrane protein, expressed on the surface of various immune cells, including T cells, macrophages, and dendritic cells. It plays a role in the immune response to intracellular pathogens and has been linked to the suppression of Th1 and pro-inflammatory responses. TIM-3 is one of the most extensively studied immunotherapeutic targets to date; however, no drugs targeting TIM-3 have been marketed internationally or domestically. Novartis’ BMS-986207^108^ is in Phase 3 clinical trials, while Bristol Myers Squibb’s BMS-986258^109^ and Incyte/Agenus’ INCAGN2390^110^ are in Phase 2 clinical trials. In China, Hengrui’s SHR-11702^111^ and BeiGene’s BGB-A425^112^ are in Phase 1 clinical trials, and other companies such as Fulong Hanlin and Fanenshi are still in the preclinical stage.

LAG-3 is another immune checkpoint molecule that is upregulated on activated T cells. It binds to MHC class II molecules on antigen-presenting cells (APCs) and transmits inhibitory signals that reduce T cell responses. Similar to PD-1 and CTLA-4, LAG-3 is overexpressed on exhausted T cells within the tumor microenvironment, aiding in cancer cells’ immune evasion. At least 16 drugs targeting the LAG-3 molecule have entered clinical research worldwide, with BMS’s BMS-986016 being the most advanced.^113^

TIGIT is primarily expressed on the surface of T cells and NK cells, and CD155 on tumor cells is a high-affinity ligand for TIGIT. TIGIT can inhibit immune activation by binding to CD155. Roche’s Tiragolumab and BMS’s BMS-986207^114^ are examples of independently developed antibody drugs targeting TIGIT, which are still undergoing clinical trials. Additionally, we have listed other ICIs that are currently on the market and their therapeutic effects in patients.

CD24, an emerging class of cancer immunotherapies that modulate immune responses within the tumor microenvironment by targeting the CD24 protein. CD24 is a cell surface molecule that is highly expressed on various cancer cells but has low expression on normal cells. It binds to the Siglec-10 receptor on macrophages, sending a “do not eat me” signal that helps tumor cells evade immune system surveillance and clearance.^115^ By blocking this signaling pathway, anti-CD24 monoclonal antibodies can enhance the phagocytic activity of macrophages against tumor cells and potentially activate a broader immune response. Several drugs targeting CD24 are currently in development. IMM47, developed by YiMab, is a pioneering anti-CD24 monoclonal antibody in China for clinical trials, showing tumor eradication in preclinical studies.^116^ Pheast Therapeutics, founded by a key figure in CD24 research, has raised substantial funds for drug development.^117^ OncoC4 advances a varied pipeline, including CAR-T and bispecific antibodies, towards clinical trials.^118^ These drugs target the CD24 protein to enhance immune responses against tumors.

### The advantages, disadvantages and challenges of mAbs

In contrast to traditional therapies like chemotherapy and radiotherapy, which are effective in killing tumor cells but also cause damage to normal cells, monoclonal antibodies therapy targets cancer cells with high specificity and reduced toxicity to health cells. Several benefits have been highlighted for mAbs therapy in treating cancers. Firstly, monoclonal therapeutic antibodies are engineered to target specific antigens or proteins with high specificity and affinity, which allows for increased effectiveness and more precise targeted treatment for patients. Secondly, due to their high specific mode of action, monoclonal antibodies pose a lower risk of side effects on health cells. Thirdly, mAbs are fast-responders, and their therapeutic effects could be observed within a short time. Last but not least, several mAbs are immune modifiers, sustaining the long-term activity of immune cells. This activated immune response might persist beyond the duration of treatment, offering long-term therapeutic protection against relapse.

However, mAbs also face several challenges and limitations. The first challenge pertains to high production costs. Monoclonal immunoglobulins are large (~150 kDa) multimeric proteins that contain numerous disulfide bonds and N-linked glycosylation sites, requiring complicated eukaryotic machinery for mass production with high purity in vitro. Large amounts of mAbs are required to achieve clinical efficacy, leading to high productions costs. Alternatively, other production systems, like microorganisms and plants, are under evaluation to lower the cost.^119,120^

Another unavoidable drawback for therapeutic drugs, including mAbs, is antibody-related side effect. The causes of these sides effects stem from following mechanisms: inherent immunogenicity, suppressive effects on other cells, and over- or long-term activation of immune system. Numerous strategies have been devised to mitigate these side effects. For example, the first therapeutic mAbs are derived from murine system, resulting in poor immune response and a plethora ofside effects. Substitution with a fully human Fc fragment or alternative engineered formats addresses immunogenic inaccessibility. Several mAbs, such as trastuzumab, rituximab, daratumumab, target specific proteins and elicit antibody-dependent cytotoxicity like complement (e.g., C1q) system activation or cell-mediated cytotoxicity via Fc domain interaction with Fcγ receptors (FcγR) on effector cells.^120^ This additional immune activation enhances therapeutic effectiveness and extends cytotoxicity. Exploring alternative antibody isotype mAbs, like panitumumab, an anti-EGFR IgG2 antibody for treating colon cancer, and engineering the Fc region through mutagenesis, such as producing defucosylated mAbs, present potential approaches to augment Fc affinity for its Fc receptors, hence diminishing antibody-dependent cytotoxicity.^121^

Importantly, low penetration efficiency and long half-life remain a challenge for therapeutic mAbs. It’s reported that no more than 20% administered dose may penetrate the target sites, with the majority remaining in the bloodstream. The remaining antibodies could interact with various types of cells including endothelial cells, monocytes, barrier sites through binding with its Fc-neonatal Fc receptor (FcRn). An extended serum half-life is associated with increased risks of immune-related adverse effects. The large molecule size of mAbs limits tumor penetration and reduces the rate of renal clearance. Besides, FcRn helps to extend the half-life of mAbs and protects them from the clearance of lysosome. Novel antibody engineering technologies are designed to overcome these shortcomings. For example, scFv fragment was developed with short half-life for imaging;^122^ medium size (~60 kDa) Diabodies have demonstrated rapid tumor uptake and clearance, suitable for imaging and radioimmunotherapy;^123^ and small antibody fragment, such as single variable domains or chemically modified antibodies e.g., anti-TNFα PEGylated Fab fragment^124^ or fusion antibody fragments with peptides,^125^ are under investigation. Other engineering methods, including fusions with effector proteins, bispecific antibodies, and intrabodies, hold promise for achieving the desired therapeutic benefits while minimizing side effects.

## Antibody drug conjugates (ADCs)

**Fig. 2:**
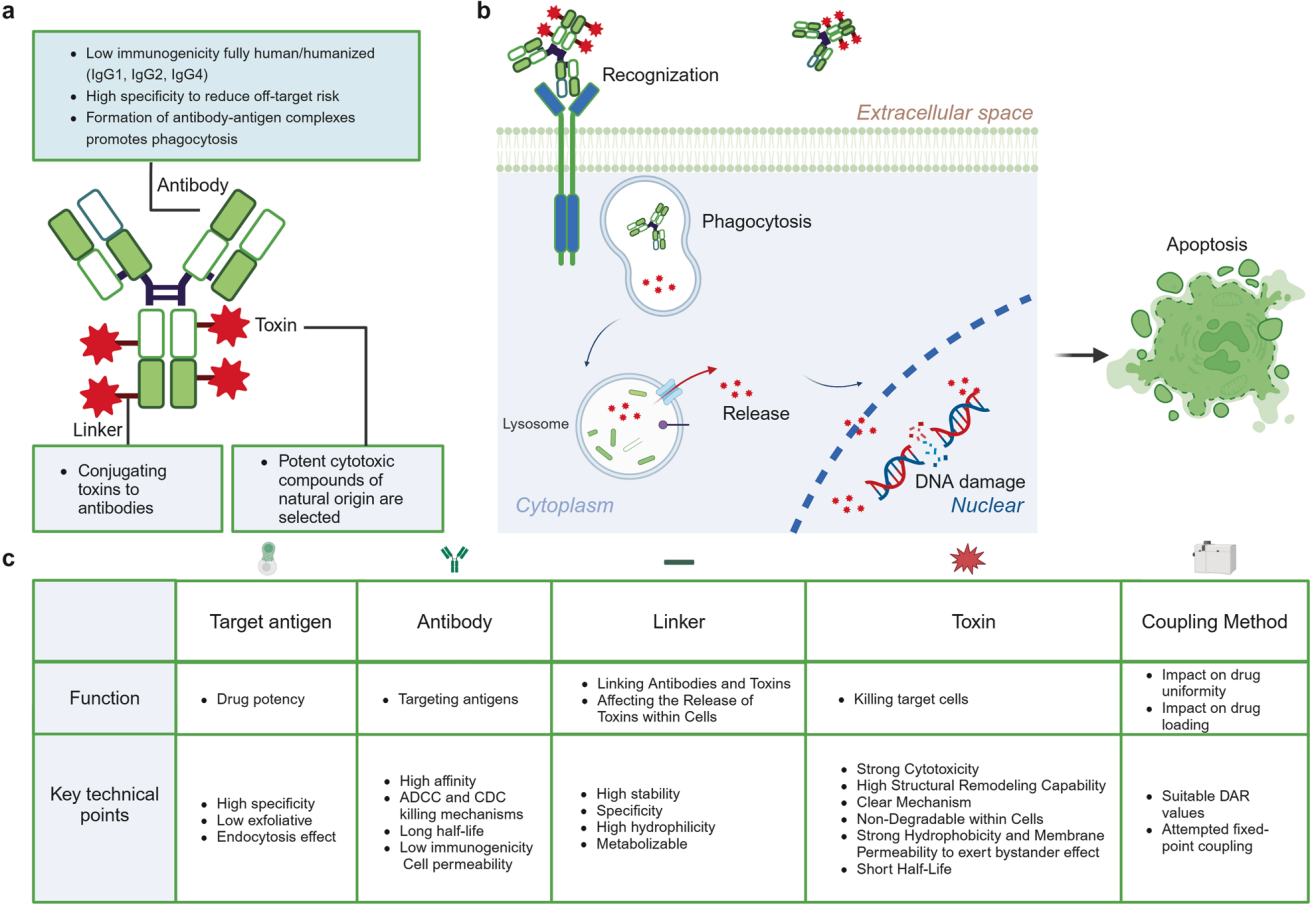
**Composition of ADCs and Mechanism of Action in Targeting Cells. a** Composition of ADCs. ADCs are composed of an antibody, a linker, and a toxin. The antibody serves as the backbone of the ADC, required to conjugate with the other two components for specific targeted endocytosis within the human body. Therefore, the demands for this part include low immunogenicity and targeting specificity. The linker is the component within the system that connects the antibody to the toxin. The toxin, the element that ultimately kills the tumor, is attached to the linker and travels with the antibody to the target site to exert its effect. **b** Mechanism of Action of ADCs Targeting Cells. ADCs exert their therapeutic effects through a process that begins with the specific binding of the antibody component of the ADC to antigens on the surface of tumor cells. Upon binding, the ADCs trigger endocytosis, a cellular process where large molecules are internalized into vesicles. These vesicles, known as endosomes, mature by moving through the cell and eventually fuse with lysosomes, where the ADCs are broken down. The cytotoxic drug component, which is linked to the antibody via a cleavable or non-cleavable linker, is then released into the lysosome or directly into the cytoplasm, where it exerts its toxic effects on the tumor cell, leading to cell death. In some cases, the released drug can also affect neighboring tumor cells through a bystander effect, enhancing the overall therapeutic impact. **c** The five core elements that influence the effectiveness of ADCs. The table outlines the requirements for various components and synthesis processes of ADC. Initially, the selection of ADC antigen substances must prioritize high specificity and low exfoliative and endocytic effects to ensure targeted action. The antibody, a key component of ADCs, demands high affinity, rapid internalization, low immunogenicity, and reliance on ADCC (Antibody-Dependent Cellular Cytotoxicity) and CDC (Complement-Dependent Cytotoxicity) mechanisms, along with a prolonged half-life for effective targeting and action. The linker should exhibit high stability to prevent rupture during circulation, enable specific release in the target area, and be hydrophilic and degradable for bystander effects. The toxin should have potent cytotoxicity, the ability to undergo structural remodeling, a clear mechanism of action, resistance to degradation within cells, strong hydrophobicity for membrane permeability to induce bystander effects, and a short half-life. The coupling method affects drug uniformity and loading, requiring optimal DAR (Drug-Antibody Ratio) values and the implementation of site-specific conjugation strategies. This figure was created with Biorender.com

Antibody-drug conjugates (ADCs) are a unique class of drugs formed by conjugating monoclonal antibodies, which specifically target antigens, with cytotoxic small molecule drugs (Fig. 2a). This innovative system is often referred to as a “biological missile” as it mimics the concept of a missile, where the antibody serves as the “guidance system” and the toxin acts as the “warhead”. This allows for precise and targeted “strikes” on specific tissue targets within the human body. Once the ADC drug enters the bloodstream, the antibody component recognizes and binds to the specific antigen present on the target cell. Subsequently, the ADC-antigen complex is internalized by the target cell through endocytosis. Within the cell, the toxin is released after being degraded by lysosomes, ultimately leading to the apoptosis of the target cell (Fig. 2b).

### Five core elements of ADCs

The effectiveness of ADCs is influenced by five core elements: target antigen, antibody, linker, toxin, and coupling method (Fig. 2c).

The target antigen for an antibody-drug conjugate (ADC) must possess high specificity to ensure precise targeting of cancer cells rather than normal cells, exhibit low exfoliative properties to minimize shedding of the antigen into the bloodstream, and have effective endocytosis capabilities to facilitate the internalization of the ADC into the cancer cells, thereby enhancing the drug’s therapeutic impact.

As the precision guidance component of antibody-drug conjugates (ADCs), antibodies specifically target and deliver the toxin carrier function. They can recognize tumor cell surface target antigens with high specificity, delivering the payload to the tumor cells and mediating the localization and endocytosis of the antibody-drug conjugate within the tumor cells. The ideal antibody characteristics necessitate prolonged circulation half-life, low immunogenicity, cell permeability, and relies on ADCC (Antibody-Dependent Cellular Cytotoxicity) and CDC (Complement-Dependent Cytotoxicity) killing mechanisms.^126^ IgG1 is the primary antibody scaffold utilized in ADCs. These IgG1 antibodies boast a prolonged blood half-life, enhanced FcγR binding efficiency, potent ADCC and CDC effects, and a reduced propensity to form oligomers.^127^ Employing humanized or human monoclonal antibodies significantly diminishes immunogenicity and alleviates autoimmune effects. Addressing the issue of antibody endocytosis involves adjusting the antibody’s size to ensure sufficient cellular penetration without jeopardizing its half-life.

The linker must possess high stability, ensuring no rupture during the cycling process, enable specific release in the target area, exhibit high hydrophilicity, and be degradable to exert bystander effects.^128^ Linkers can be divided into two main categories based on their performance: cleavable linkers (chemical cleavage linkers, enzyme catalyzed cleavage linkers, photo-cleavable linkers) and non-cleavable linkers (sulfide bond linkers, maleimide bond linkers); cleavable linkers are a prerequisite for exerting bystander killing effect, hence becoming the mainstream trend of ADC linkers. The development directions of ADC linkers are to increase hydrophilicity (reduce ADC clearance rate, broaden compatibility with hydrophobic toxins) and increase the effective payload number on a single linker.^128^

The toxin must exhibit strong cytotoxicity, high structural remodeling capability, a clear mechanism, non-degradability within cells, demonstrate strong hydrophobicity, and possess membrane permeability to induce bystander effects, while having a short half-life.^128^ The coupling method impacts drug uniformity and loading, necessitating suitable DAR (Drug-Antibody Ratio) values and attempted fixed-point coupling.^128^

### FDA approved ADC drugs

Currently approved ADC drugs primarily target specific proteins overexpressed on tumor cells. HER2 is the most prominent target for ADC development globally, and Ado-trastuzumab emtansine (Kadcyla), Trastuzumab deruxtecan (Enhertu), and Trastuzumab emtansine (T-DM1), which are designed to target HER2-positive cancer cells, have been approved for use in treating HER2-positive breast cancer and other types of cancer. DS-8201 (Trastuzumab Deruxtecan) is a novel antibody-drug conjugate (ADC) composed of a humanized anti-HER2 antibody and an irinotecan-class chemotherapy drug. It has demonstrated promising antitumor activity in various types of cancer, including breast cancer, gastric cancer, and colorectal cancer. Particularly in patients with breast cancer that exhibits low expression of HER2, DS-8201 has shown a significant improvement in therapeutic outcomes compared to other treatment options. Based on the results of the DESTINY-Breast04 study, the FDA has granted accelerated approval for the use of DS-8201 in the treatment of HER2-low expressing breast cancer.^129^ As of March 2024, the FDA has approved a total of 15 ADC drugs and there are over 500 clinical trials in progress. The details of 15 FDA approved ADC drugs as shown in the Table 2.^130–133^

**Table 2:** Summary of ADC drugs approved by the FDA as of march 2024

Drug	Maker	Target	Toxin	Linker	Indications	DAR	Approval Date	Effect
Mylotarg	Pfizer	CD33	Calicheamicin	hydrazone	AML	2 ~ 3	2000	Only Chemotherapy mPFS: 9.5 months, Combined Chemotherapy mPFS: 17.3 months
Adcetris	Seagen	CD30	MMAE	dipeptide	Hodgkin’s lymphoma	4	2011	2-year PFS at 82.1%, Control at 7.2%, progession risk lowered by 34%
Kadcyla	Roche	HER2	DM1	organic sulphide	Early-stage and metastatic HER2+ breast cancer	3.5	2013	mPFS at 6.2 months, control at 3.3 months; ORR at 31.3% vs. 8.6%
Besponsa	Pfizer	CD22	Calicheamicin	hydrazone	BCP-ALL	5 ~ 7	2017	CR at 80.%, Chemotherapy at 29.4%, mOS at 7.7 vs. 6.7 months
Lumoxiti	AstraZeneca	CD22	PE38	mc-VC-PABC	HCL	/	2018	ORR at 75%, CR at 36%
Polivy	Roche	CD79β	MMAE	dipeptide	LBCB	3.5	2019	CR at 40, control at 19%, mOS at 12.4 vs. 4.7 months
Enhertu	Daiichi Sankyo	HER2	Dxd	Boc-Gly-Gly-Phe-Gly-OH	Breast/Stomach Cancer	8	2019	mOS at 12.5 months, Chemotherapy at 8.4 mpnths, ORR at 41% vs. 11%
Padcev	Seagen	Nectin-4	MMAE	mc-VC-PABC	Bladder Cancer	4	2019	mOS at 12.9 months, Chemotherapy at 9.0 months
Trodelvy	Immunomdecis	TROP-2	SN38	CL2A	TNBC	8	2020	mPFS at 4.8 months, Chemotherapy at 1.7 months; mOS at 11.8 vs. 6.9
Belenrep	GSK	BCMA	MMAF	Not Cleavable	multiple myeloma	4	2020	ORR at 32%, mPFS at 11 months, mOS at 11.8 vs. 6.9
Akalux	Rakuten Medical	EGFR	IRDye700DX	N/A	Head and Neck Cancer	/	2020	ORR at 43.3%, CR at 13.3%
Zynlonta	ADC Therapeutics	CD19	PBD	dipeptide	LBCB	2.3	2021	ORR at 48.3%, CR at 24.8% (Including failed CAR-T patients)
RC48 -ADC	RemeGen	HER2	MMAE	mc-VA-PABC	Stomach cancer	3.5	2021	ORR at 40%, mPFS at 6.3 months
Tivdak	Genmab/Seagen	TF	MMAE	Enzyme-cleaved	Cervical cancer	4	2021	OOR at 24%, DOR at 8.3 months
Elahere	ImmunoGen	FRα	DM4	Non-cleavable	ovarian cancer	3.4	2022	ORR 42.3%, mPFS 5.62 months, OS 16.5 months
Trastuzumab Deruxtecan	Daiichi Sankyo and AstraZeneca	HER2	Deruxtecan	Chemical cleavable	Breast cancer	8	2022	ORR 43.2%, DCR 79.5%, mPFS 5.6 months, 12.8 months

*mPFS* Median Progression-Free Survival, *ORR* Objective Response Rate, *CR* Complete Response, *DCR* Disease Control Rate, *DOR* Duration of Response, *mOS* Median Overall Survival, *PFS* Progression-Free Survival, *LBCB* Large B-Cell Lymphoma, *BCP-ALL* B-Cell Precursor Acute Lymphoblastic Leukemia, *HCL* Hairy Cell Leukemia, *TNBC* Triple-Negative Breast Cancer, *EGFR* Epidermal Growth Factor Receptor, *BCMA* B-Cell Maturation Antigen, *MMAE* Monomethyl auristatin E, *TROP-2* Trophoblast Cell Surface Antigen 2, *TF* Tissue Factor, *HER2* Human Epidermal Growth Factor Receptor 2, *FRα* Folate Receptor Alpha. Data accessed on 24th March 2024

### The advantages, disadvantages, and challenges of ADC drugs

As demonstrated above, the greatest advantage of ADCs lies in their reliance on the specific binding of antigen-antibody targeting. This helps to reduce systemic toxic side effects that are characteristic of chemotherapy treatments. Additionally, unlike conventional monoclonal antibodies, ADCs exert their tumor-killing function through the toxin carried by the linker, which allows for greater design flexibility. Furthermore, ADCs can be tailored to target a variety of cancers based on the selection of antigens and antibodies. Moreover, the design of ADCs permits precise control of the ratio of drug to antibody (DAR, Drug-to-Antibody Ratio), which aids in optimizing therapeutic efficacy and safety.

The main drawbacks of ADCs, such as their low internalization, low efficiency, and target-off toxicity, should be addressed by creating more effective ADCs, such as the introduction of bispecific ADCs. For example, ZW38, an asymmetric bispecific CD19-directed CD3 T cell engager antibody, has a significantly higher affinity (>30-fold) to CD3 + T cells than CD19 + B cells. It is specifically designed to mediate effective T cell-guided targeting B cell reduction while eliciting a more “controlled” T cell activation compared to blinatumomab, thereby leading to lower toxicity.^134^ Another preclinical study has proved that bispecific ADCs for EGFR/c-MET and HER2/PRLR exhibit improved internalization, increased affinity, and decreased toxicity.^135,136^ Additionally, striking a balance between therapeutic activity within a confined DAR and the toxicity of payloads presents another challenge. Novel approaches to alter payloads with versatile functional groups, such as amine or thiol groups, will provide new perspectives on addressing this issue.^128^ Meanwhile, several site-specific conjugation techniques are being developed to generate homogeneous ADCs, which aim to increase efficiency and decrease toxicity.^137^

The development of ADCs still faces challenges. Certain types of cancer lack effective neoantigens. For instance, the discovery and validation of neoantigens are time-consuming, and the expression of antigens varies among individuals. Additionally, tumors may alter the expression of their surface antigens to evade the immune system’s attack, which can make it difficult to identify suitable antibodies. Furthermore, ADCs undergo complex metabolic processes. Due to their diverse designs, ADCs lack uniform metabolic properties. Even ADCs targeting the same antigen may exhibit differences in plasma stability, in vivo metabolism, PK/PD relationships, and adverse reactions owing to variations in antigen epitope recognition, linker sites, coupling chemistry, and the choice of small molecule toxins.^138,139^ Additionally, ADCs still have toxicities, such as on-target/off-tumor toxicity and off-target/off-tumor toxicity, with the latter being caused by the premature release of toxins into the bloodstream, non-tumor tissues, or the tumor microenvironment.^140^ Furthermore, the mechanisms of ADC resistance have not been thoroughly studied, and the production and quality control of ADCs also pose difficulties, all of which affect the production and clinical application of ADC drugs.

## Cell therapy

Cell therapy, also known as cellular therapy, is a cutting-edge approach in medicine that utilizes living cells to against cancers. Ongoing research and advancements in cell therapy continue to pave the way for revolutionary breakthroughs in healthcare, providing hope for patients in need of novel and effective treatment options. Currently, popular tumor cell therapies include Chimeric Antigen Receptor T-Cell Therapy (CAR-T), T-Cell Receptor Modified T cells (TCR-T), Tumor-Infiltrating Lymphocytes (TIL), Chimeric Antigen Receptor-Modified Natural Killer (CAR-NK) cells, T-Cell Receptor Modified T-Cells (TCR-T) and Chimeric Antigen Receptor-Modified Macrophages (CAR-M).

### CAR-T

**Fig. 3:**
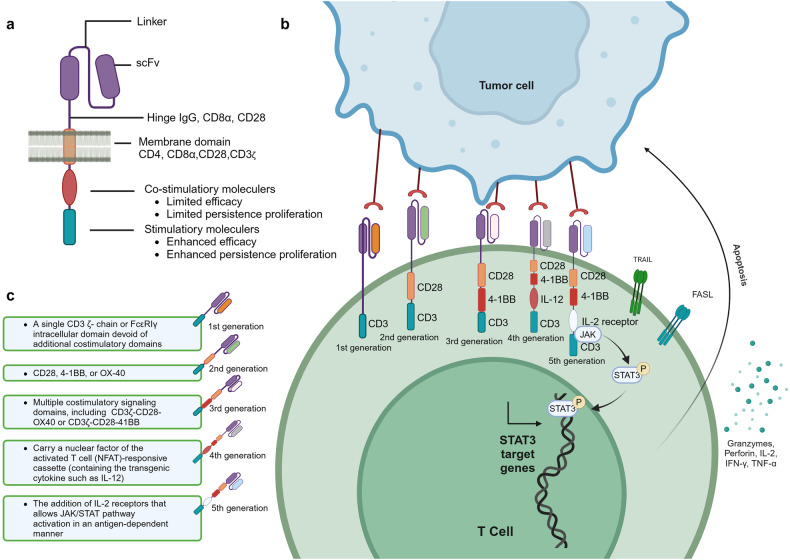
**The composition and generation of CAR-T. a** Illustrates the structure and mechanism of action of a Chimeric Antigen Receptor (CAR). The CAR is composed of three main domains: the extracellular domain, which includes the antigen recognition domain (scFv) and the hinge region; the transmembrane domain that anchors the receptor in the cell membrane; and the intracellular domain responsible for signaling. The scFv is engineered to recognize specific tumor-associated antigens, such as CD19, CD20, and CD22. The hinge region allows for flexibility in the CAR’s structure, while the transmembrane domain links the extracellular recognition capabilities to the intracellular signaling pathways. The intracellular domain typically contains co-stimulatory motifs that enhance T cell activation upon antigen recognition. **b** The therapeutic process of CAR-. Genetically engineered CAR-T cells are infused into the patient, where they specifically recognize and bind to tumor antigens via their CARs. This interaction leads to the activation of the CAR-T cells and the release of cytotoxic molecules, such as perforin and granzyme B, which induce apoptosis in the tumor cells. **c** The evolution of CAR-T. It outlines the five generations of CAR-T cell development. First-generation CAR-T cells had basic signaling domains but lacked co-stimulatory signals, resulting in limited in vivo proliferation and clinical efficacy. Second-generation CAR-T cells included additional co-stimulatory domains, significantly improving their potency and persistence. Third-generation CAR-T cells further enhanced tumor lysis and cytokine secretion by incorporating dual co-stimulatory molecules. Fourth-generation CAR-T cells were designed with controllable suicide genes and pro-inflammatory cytokines for enhanced solid tumor targeting. Fifth-generation CAR-T cells, or “off-the-shelf” universal CAR-T cells, are created by CRISPR/Cas9 gene editing to generate allogeneic T cells, addressing potential GVHD issues. This figure was created with Biorender.com

The treatment principle of CAR-T involves extracting the patient’s own T cells, using gene editing technology to transfect the chimeric antigen receptor (CAR) gene, expanding the modified T cells in vitro, and then infusing them back into the patient. The CAR structure consists of three distinct domains: the extracellular domain, the transmembrane domain, and the intracellular domain. The extracellular domain of CAR includes the antigen recognition domain, also known as the single-chain variable fragment (scFv), and the hinge region. The scFv is composed of the variable regions of both the light and heavy chains of an antibody, connected by a peptide linker. CARs are being developed to target various tumor-associated antigens such as CD19, CD20, CD22.^141^ The hinge region connects the scFv to the transmembrane domain. The transmembrane domain serves as a link between the extracellular domain and the intracellular signaling domain of the CAR. Commonly used transmembrane domains are derived from proteins such as CD4, CD8α, CD28, and CD3ζ (Fig. 3a).^141^

The mechanism of CAR-T cell therapy involves several key steps. CAR-T cells are genetically engineered T cells that are designed to target specific antigens present on the surface of tumor cells. Once infused into the patient’s body, CAR-T cells recognize and bind to these tumor antigens through theCAR. This binding triggers the activation of the CAR-T cells, leading to the release of cytotoxic effector molecules such as perforin and granzyme B. These substances directly induce apoptosis, or programmed cell death, in the tumor cells (Fig. 3b). Furthermore, CAR-T cells can recruit other immune cells, such as natural killer cells and macrophages, to the tumor site.^142^ This recruitment is achieved through the secretion of cytokines by the CAR-T cells. The immune cells work together to attack and eliminate the tumor cells. One of the remarkable features of CAR-T cell therapy is the potential to form memory T cells. These memory T cells can persist in the body after the initial treatment and provide long-term protection against the recurrence of tumor cells. This memory response contributes to the sustained anti-tumor effects of CAR-T cell therapy. Understanding the mechanism of CAR-T cell therapy is crucial for its successful application in treating various types of cancer.

The development process of CAR-T has gone through five generations. The first-generation CAR-T cells contained intracellular signaling domains but lacked co-stimulatory molecules.^143^ These CAR-T cells had limited proliferation in vivo and were not effective in killing tumor cells on a large scale, resulting in unsatisfactory clinical trial outcomes. The second-generation CAR-T cells added a co-stimulatory domain (CD28, 4–1BB, OX40, etc.) to the intracellular domain. After the single-chain antibody on the extracellular domain recognized the tumor cells and bound to the tumor antigen, the T cells could simultaneously receive antibody stimulation signals and co-stimulatory signals.^144^ This made the second-generation CAR-T cells far more potent than the first-generation, with longer survival time and enhanced proliferation and tumor-killing ability. The third-generation CAR-T cells incorporate two co-stimulatory molecules simultaneously to enhance tumor lysis ability and increase cytokine secretion, thereby boosting the killing power against tumors. Common combinations include CD28 + 4–1BB or CD28 + OX40 as dual co-stimulatory molecules.^145^ Whether the third-generation CAR-T cells are superior to the second-generation ones still needs to be proven in clinical trials. The design of fourth-generation CAR-T cells involve adding controllable suicide genes and pro-inflammatory cytokines (such as IL-12, IL-15, IL-18) to the CAR structure, which allows for controlled survival time of CAR-T cells in the body and enhances the efficacy in killing solid tumors.^142^ The fifth-generation CAR-T cells, known as “off-the-shelf” universal CAR-T cells, are designed to disrupt the TCR genes and HLA class I genes of T cells using CRISPR/Cas9 gene editing (Fig. 3c). This generates allogeneic universal CAR-T cells and eliminates graft-versus-host disease (GVHD) concerns.^142^

#### The challenges and strategies about CAR-T cell therapy

**Fig. 4:**
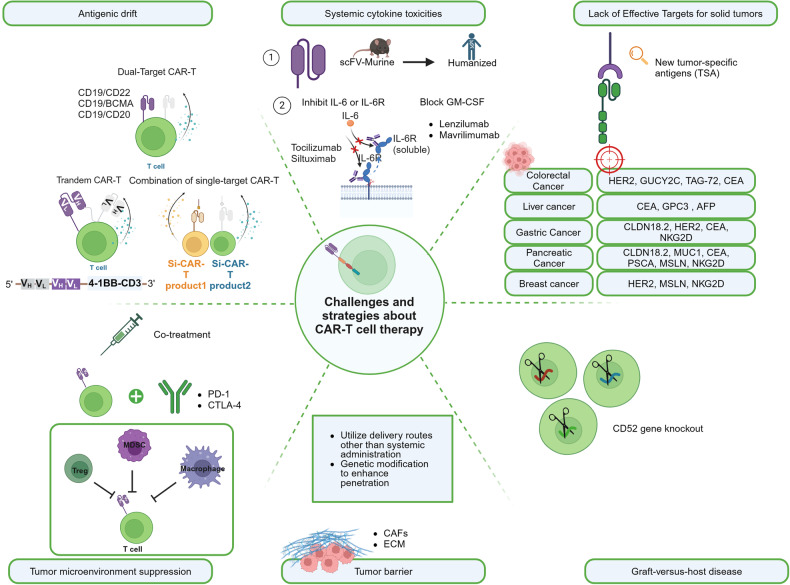
**The challenges and strategies about CAR-T cell therapy**. The main challenges currently faced in CAR-T therapy include: Antigenic drift, Systemic cytokine toxicities, Lack of effective targets for solid tumors, Tumor microenvironment suppression and epitope expansion, Tumor barrier, Graft-versus-host disease (GVHD), and Host immune rejection, along with their corresponding primary solutions. This figure was created with Biorender.com

In addition to the practical challenges of cost issues, limitations in meeting inclusion criteria, and unforeseen challenges during the gap period between leukapheresis and infusion, there are also ongoing technical barriers that need to be overcome in the field of CAR-T therapy (Fig. 4).

##### Antigenic drift

Antigenic drift is an immunotherapy resistance commonly observed in CAR-T therapy. Although, phase I trials of CD19 CAR T cells in patients with B-cell acute lymphoblastic leukemia (B-ALL) showed response rates between 70% and 90%, there are 7–25% patients resulting in CD19 antigen loss. Other studies demonstrated that loss of BCMA and GPRC5D is observed in 4% and 35% myeloma patients after treatment with BCMA CAR-T and GPRC5D CAR-T, respectively.^146^

To overcome these challenges, various strategies have been explored. One approach is the use of dual-target CAR-T, which targets multiple antigens simultaneously. In CD19 CAR-T therapy, it is generally accepted to use a combination of CD19 with CD22, BCMA, and CD20.^147^ A bispecific CAR-T (CD19–22.BB.z-CAR) dual-targeting CD19 and CD22 was investigated in a phase I study for patients with large B cell lymphoma (LBCL) and relapsed/refractory B cell acute lymphoblastic leukemia (R/R B-ALL). For both B-ALL and LBCL, the complete response (CR) rates were 29% and 100%, respectively. Minimal residual disease-negative (MRD-) complete remission was attained by 88% of R/R B-ALL patients. Four (29%) patients with LBCL and five (50%) patients with B-ALL exhibited low or negative CD19 expression at the time of progression after the treatment.^148^ A similar result was shown in 13 B-ALL patients, where 84.6% of patients achieved CR with dual CD19xCD22 CAR-T cell (SCRI-CAR19x22) therapy, and 95% were MRD-. In three out of the four relapses, CD19 expression remained negative.^149^ Aftertreatment with CD19/BCMA dual-targeting Fastcar-T GC012F for patients with relapsed/refractory B-cell non-Hodgkin’s lymphoma (R/R B-NHL), the 3-month overall response rate was 100%, with 77.8% (7/9) reaching CR and no relapse was noted within the follow-up period.^150^

Another strategy is tandem CAR-T, where a single CAR construct contains two single-chain variable fragments (scFvs) to target different antigens on one cancer cell surface. In a phase I clinical investigation, by day 28 following therapy with tandem bispecific anti-CD20, anti-CD19 (LV20.19) CAR T cells, 18 (82%) of the R/R B-ALL patients experienced an overall response, with 14 (64%) achieving a CR, and 4 (18%) having a partial response (PR). Remarkably, patients who experienced treatment failure or relapsed did not exhibit loss of the CD19 antigen.^151^ In Dai et al.’ study, after receiving another bispecific tandem CD19/CD22 CAR-T therapy, the patients demonstrated a favorable outcome, with a high MRD- complete response rate (100%) reached in B-ALL patients (n = 6); However, one patient relapsed at 5 months post-treatment with decreased CD22 density and negative CD19 expression.^152^ Additionally, the combination of different single-target CAR-T therapies is being investigated. These strategies aim to enhance the efficacy and durability of CAR-T therapy and improve outcomes for patients. Sequential administration of two single-target CAR-T therapies, such as the CAR22/19 cocktail CAR-T therapy, was investigated by Huang et al.^153^ Among the 50 R/R B-ALL patients, 48 (96%) achieved CR or complete remission with incomplete count recovery (CRi) with 47 (94%) obtaining MRD^-^ CR/CRi. 23 individuals relapsed, but none of them exhibited evidence of CD19 or CD22 antigen loss.

##### Systemic cytokine toxicities

Upon infusion, CAR-T cells become activated and rapidly proliferate, causing a massive release of cytokines and triggering non-specific inflammatory reactions. These may lead to systemic cytokine toxicities, including cytokine release syndrome (CRS), haemophagocytic lymphohistiocytosis (HLH)/macrophage activation syndrome (MAS), and immune effector cell-associated neurotoxicity syndrome (ICANS). To address these toxicities, one approach is modifying the CAR structure by replacing murine counterparts with humanizing or fully human antibody fragments, aiming to reduce immunogenicity and improve safety. Also, targeting specific cytokines associated with severe CRS, such as IL-6 and IL-1, has been explored [37]. Anti-inflammatory drugs such as tocilizumab and siltuximab can inhibit the action of interleukin-6 (IL-6) and the interleukin-6 receptor (IL-6R), thereby reducing the CAR-T-associated CRS without impairing CAR-T activities or causing T cell apoptosis.^154–159^

Inhibiting the inflammatory cascade initiated by GM-CSF (granulocyte-macrophage colony-stimulating factor) has also shown promise in mitigating cytokine toxicities effects.^160^ GM-CSF inhibitors such as Lenzilumab and Mavrilimumab can block the action of GM-CSF, thereby reducing the activation of inflammatory cells and the release of inflammatory cytokines.^161^

##### Lack of Effective Targets for solid tumors

Unlike the targets for hematologic malignancies that are mostly single and specific, tumor-specific antigens (TSA) are rare in solid tumors. Common tumor-associated antigen (TAA) targets include CEA, HER2, GPC3, EpCAM, etc. are often expressed on vital organs, which severely limit the application of CAR-T in solid tumors.^162^ The continuous discovery of highly expressed and CAR-T-developable targets has led to rapid growth of clinical pipelines, such as GPC3 highly expressed in liver cancer, CLDN18.2 highly expressed in gastric and pancreatic cancer, EGFRvIII in glioblastoma.^163,164^

##### Tumor microenvironment suppression and epitope expansion

In solid tumor, immune checkpoint such as PD-1, TIM-3, or CTLA-4 are commonly overexpressed and may cause the exhaustion of CAR-T cells. The tumor microenvironment (TME) contains immunosuppressive cells such as regulatory T cells (Tregs), myeloid-derived suppressor cells (MDSCs), and M2 macrophages. These cells release cytokines like transforming growth factor-beta (TGFβ) and interleukin-10 (IL-10) within solid tumors, which diminish the effectiveness of CAR-T cells in fighting tumors.^165^ To overcome this, strategies include combining CAR-T cells with immune checkpoint inhibitors such as PD-1 inhibitors in immunotherapy and genetically modifying CAR-T cells to enhance their immune response and resistance to inhibitory factors, thereby improving their anti-tumor activity.^166^ Several clinical trials on the combination of CAR-T and immune checkpoints blockades are summarized in Table 3.

**Table 3:** Representative clinical trials for the combination of ICIs and CAR-T therapy

Clinical Trials No.	Phase	No. Infused Patients	Combination regimens	Cancer Type	Preliminary Results	
			ICI	CAR-T Target		
NCT01822652	I	11	Pembrolizumab	GD2	Neuroblastoma	6 PD, 2 CR (after salvage), 5 SD
NCT02414269	I	27	Pembrolizumab	Mesothelin	Malignant pleural diseases, comprising metastatic lung and breast cancers and malignant pleural mesothelioma	2 CR, 8 SD (>6mons)
NCT03287817	I	19	Pembrolizumab	CD19/22 dual target	r/r DLBCL	64% ORR, 55% CRR
NCT03630159	Ib	4	Pembrolizumab	CD19	r/r DLBCL	1 PR, 2 PD
NCT03726515	I	7	Pembrolizumab	EGFRvIII	EGFRvIII + , MGMT-unmethylated glioblastoma	Low efficacy, 7 PD, median PFS: 5.2mons, median OS: 11.8months
NCT04991948	Ib	Estimated 34	Pembrolizumab	NKG2D	Colorectal Cancer	2 Deaths reported. Paused
NCT04995003	I	Estimated 25	Pembrolizumab or nivolumab	HER-2	Advanced Sarcoma	NA
NCT04003649	I	Estimated 60	Nivolumab and Ipilimumab	IL13Ra2	Glioblastoma	NA
NCT04539444	II	16	Tislelizumab	CD19/22 dual target	R/R B-NHL	CR 11, 1-year PFS: 68.8%, 1-year OS: 81.3%
NCT04381741	Ib	8	Tislelizumab	CD19	R/R DLBCL	4 CR, 1 PR, 2 PD
NCT02926833	I/II	28	Atezolizumab	CD19	DLBCL	75% ORR, 46% CR, 29% PR,7%SD, 14%PD
NCT02706405	I	29	Durvalumab	CD19	R/R LBCL	35% ORR, 27% CR
NCT03310619	I/II	Estimated 77	Durvalumab, Nivolumab, Relatlimab	CD19	R/R aggressive B-cell NHL	NA

*R/R DLCBL* Relapsed/Refractory Diffuse Large B Cell Lymphoma, *R/R B-NHL* relapsed/refractory B-cell non-Hodgkin lymphoma, *R/R LBCL* Relapsed/Refractory Large B Cell Lymphoma, *CR* complete response, *PR* partial; response, *PD* progression disease, *SD* stable disease, *ORR* overall response rate, *PFS* progression-free survival, *OS* overall survival, *NA* not applicable

##### Tumor barrier

The immunosuppressive tumor microenvironment and physical tumor barriers, such as the tumor stroma, can restrict the infiltration and migration of CAR-T cells, limiting their effectiveness in treating solid tumors. One main reason is the lack of relevant receptors on CAR-T cells that match the chemokines secreted by solid tumors, which hinders their homing ability to the tumor site. Strategies include utilizing delivery routes other than systemic administration, such as intrathoracic injection, which has demonstrated superiority in treating malignant pleural mesothelioma.^167^ Another approach involves genetic modification of CAR-T cells to enhance their penetration, such as expressing heparinase, an enzyme that degrades HSPG, which can enhance tumor infiltration and anti-tumor activity.^168^

##### Graft-versus-host disease (GVHD) and host immune rejection

Allogeneic CAR-T therapies face two main challenges: graft-versus-host disease (GVHD) and host immune rejection of foreign cells, both of which can limit the effectiveness and persistence of anti-tumor activity. To address these challenges, most allogeneic CAR-T therapies use gene editing to knock out endogenous T cell receptors (TCRs) and other proteins that may trigger host immune rejection. Gene editing tools like TALENs, ZFNs, or CRISPR/Cas9 are commonly used to target the TRAC gene and permanently knock out proteins that elicit immune rejection.^169^ However, gene editing also carries potential safety risks such as off-target effects and chromosomal abnormalities. Another strategy involves CD52 gene knockout to provide allogeneic T cells with resistance to lymphocyte depletion.^170^ Disrupting the B2M locus can also prevent host immune system destruction of allogeneic cells by preventing the formation of HLA-1 molecules on T cell surfaces and avoiding recognition as foreign entities.^171^

### CAR-NK

**Fig. 5:**
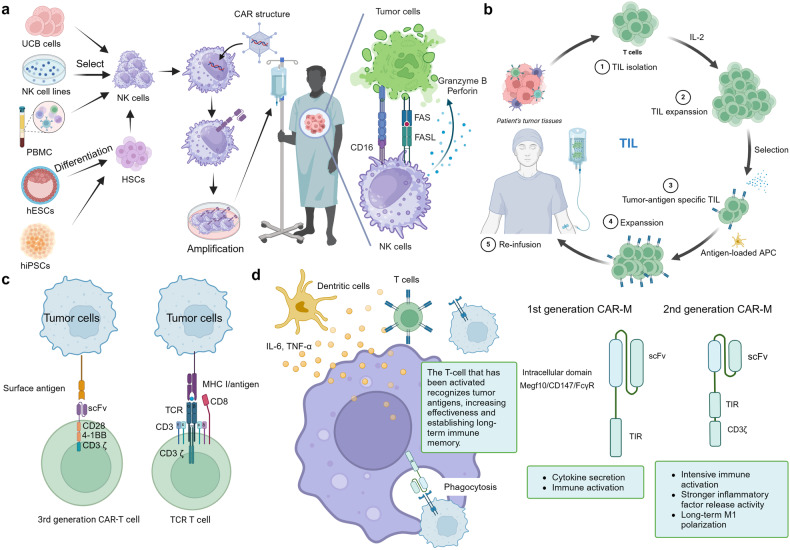
**Other kind of cell therapy. a** Preparation of CAR-NK and the mechanisms of CAR-NK cell. The preparation of CAR-NK cells involves isolating NK cells from sources such as umbilical cord blood or hematopoietic stem cells, followed by genetic modification to integrate a CAR construct that includes an antigen recognition domain. These cells are then expanded in vitro before being infused back into the patient. Once in the body, CAR-NK cells utilize their CAR to recognize and bind to specific tumor antigens, leading to their activation and the subsequent release of cytotoxic granules and cytokines to eliminate cancer cells. Additionally, the Fc receptor CD16 on NK cells can mediate ADCC, further enhancing their tumoricidal activity. **b** Preparation of TIL. The preparation of tumor-infiltrating lymphocytes (TIL) involves isolating immune cells from a patient’s tumor tissue, where they have naturally infiltrated. The process typically includes surgical removal of the tumor mass, followed by the mechanical and enzymatic dissociation of the tissue to obtain a single-cell suspension. The cells are then cultured in vitro, and the TILs, which are often CD8+ T cells, are selectively expanded through various techniques, such as interleukin-2 (IL-2) stimulation. Once a sufficient number of TILs are obtained, they are infused back into the patient as part of the adoptive cell transfer therapy, aiming to boost the immune system’s ability to target and destroy cancer cells. **c** The structure comparation between TCR-T and CAR-T. TCR-T cells are genetically modified to express a specific T-cell receptor that recognizes MHC-presented antigens, allowing them to target a broad range of tumor-associated antigens, including those derived from intracellular proteins. In contrast, CAR-T cells are engineered to express a chimeric antigen receptor that includes an antibody-derived antigen-binding domain, enabling them to target cell surface antigens without MHC restriction. **d** The overview of CAR-M (workflow and generation). Once inside the patient’s body, the CAR-M cells use their CAR to identify and attach to tumor cells, which triggers the activation of the macrophages, leading to the subsequent engulfment and destruction of the tumor cells. Additionally, they secrete pro-inflammatory cytokines that recruit other cells from the immune microenvironment to join in the attack against the tumor. CAR-Ms are differentiated into first and second generations. The second generation includes an extra CD3 domain compared to the first, endowing it with enhanced pro-inflammatory properties and sustained M1 macrophage activation. This figure was created with Biorender.com

CAR-NK involves introducing a CAR into NK cells, enabling them to specifically recognize and eliminate tumor cells. The cytotoxicity mechanisms of CAR-NK cells primarily include the secretion of cytotoxic granules such as Perforin and Granzyme, which directly kill target cells^172^ (Fig. 5a). Additionally, CAR-NK cells can express tumor necrosis factors like FasL and TRAIL, which induce apoptosis in target cells by binding to them.^173^ Another mechanism is ADCC, where CAR-NK cells express FcγRIII (CD16) receptors that bind to the Fc region of tumor antigen-specific antibodies.^174^ This activation leads to cytotoxicity and mediates the killing of target cells (Fig. 5a).

CAR-NK cell therapy offers several advantages over other cell therapies. Firstly, NK cells are widely available from various sources such as NK92 cell lines, PBMC, CD34 + HPC, UCB cells, and iPSC.^175^ Among them, NK92 cell lines are commonly used in clinical trials due to their unlimited proliferation capacity in vitro, ease of genetic modification, and no need for patient blood extraction. Secondly, CAR-NK cells are safer than other cell therapies due to their short survival time, reducing damage to non-tumor cells, and lower risk of cytokine release syndrome (CRS), as they produce mainly IFNγ and GM-CSF, which reduces the risk of CRS.^176^ Finally, CAR-NK cells are easier to produce for off-the-shelf use, as allogeneic NK cells do not express individual-specific TCR, and the risk of GVHD is much lower than that of allogeneic T cell therapy, making it more suitable for industrialization and large-scale production^177^ (Fig. 5a). Preclinical studies have examined several candidate targets for engineered CAR-NK therapy, including CD19 and CD22 for B-cell malignancies; CD38 and CD138 for multiple myeloma (MM); CD3, CD5, and CD7 for T-cell malignancies; and CD24, EGFR, HER-2 and NKG2D for solid tumors. There are ongoing clinical trials to assess the potential of CAR-NK (Table 4). In case of cord blood-derived CAR CD19-NK treatment for B-cell malignancies, one completed clinical trial (NCT03056339) demonstrated a promising efficacy with an ORR of 48.6%, and 1-year OS, and 1-year PFS rate of 68% and 32%, respectively. This suggests CAR-NK as a potential alternative for oncology treatment.^178^

**Table 4:** Representative clinical trials on CAR-NK cell therapy for oncology treatment

Clinical trial No.	Phase	Estimated Enrolled Participants	Target	Source of NK cells	Malignancies	Preliminary Results
NCT00995137	I	14	CD19	Peripheral blood	B-AML	NA
NCT01974479	I	20	CD19	Peripheral blood	B-AML	Suspended
NCT03056339	I/II	37	CD19	Cord blood	CD19 + B cell tumors	48.6% ORR, 1-year OS: 68%, 1-year PFS: 32%
NCT03383978	I	42	HER-2	NK-92	Glioblastoma	NA
NCT03415100	I	30	NKG2D ligands	Peripheral blood	Metastatic Solid Tumours	NA
NCT03656705	I	2	Unknown	NK92	NSCLC	NA
NCT03692663	Early Phase I	9	PSMA	Unknown	mCRPC	NA
NCT03824964	Early Phase I	10	CD19/CD22	Unknown	R/R BCL	NA
NCT03692767	Early Phase I	9	CD22	Unknown	R/R BCL	NA
NCT03690310	Early Phase I	9	CD19	Unknown	R/R BCL	NA
NCT03692637	Early Phase I	30	Mesothelin	Peripheral blood	EOC	NA
NCT04245722	I	98	CD19 ± CD20 antibody (rituximab or obinutuzumab)	iPSC	R/R BCL or CLL	1 case repored PR
NCT04623944	I	90	NKG2D ligands	Peripheral blood	R/R AML or Myelodysplastic Syndromes	4 CR/Cri
NCT05215015	Early Phase I	18	CD33/CLL1	Unknown	AML	NA
NCT04639739	Early Phase I	9	CD19	Unknown	NHL	NA
NCT03940833	I/II	20	BCMA	NK92	Multiple Myeloma	NA
NCT03940820	I/II	20	ROBO	Unknown	Solid tumors	NA
NCT03941457	I/II	9	ROBO1	Unknown	Pancreatic Cancer	NA
NCT03383978	I	42	HER2	NK92	Recurrent HER2-positive Glioblastoma	NA
NCT05336409	I	75	CD19	iPSC	Relapsed or Refractory CD19-Positive B-Cell Malignancies	NA
NCT04747093	I/II	12	CD19	Induced-T cell-like NK cells	Refractory B Cell Malignancies	NA
NCT04796675	I	27	CD19	Cord blood	AML, CLL, NHL	NA
NCT04887012	I	25	CD19	Peripheral blood	Refractory/Relapsed B-cell NHL	NA
NCT05020678	I	150	CD19	Peripheral blood	R/R NHL, CLL or B-ALL	8 CR, 3 PD, 1 SD
NCT05137275	Early Phase I	56	Trophoblast glycoprotein 5T4	Unknown	Locally advanced or metastatic solid tumors	NA
NCT05008536	Early Phase I	27	BCMA	Umbilical & Cord Blood	Relapsed and refractory MM	NA
NCT05247957	I	9	NKG2D	Cord blood	Recurrent refractory AML	NA
NCT05213195	I	38	NKG2D	Unknown	Refractory Metastatic Colorectal Cancer	NA
NCT04847466	II	55	PD-L1 combined with pembrolizumab and N-803	NK-92	Advanced gastric or head and neck cancer	NA
NCT05008575	I	27	CD33	Unknown	R/R AML	6 of 10 MRD-negative CR
NCT05194709	Early Phase I	40	Trophoblast Glycoprotein 5T4	Unknown	Advanced Solid Tumors	NA
NCT05379647	I	24	CD19 + CD20 antibody (rituximab)	iPSC	R/R BCL	NA
NCT05182073	I	168	BCMA	iPSC	MM	NA
NCT05110742	I/II	48	CD5	Cord Blood	Relapsed/ Refractory Hematological Malignances	NA
NCT05092451	I/II	94	CD70	Cord Blood	Relapse/ Refractory Hematological Malignances	NA
NCT05336409	I	75	CD19	iPSC	CD19-Positive B-Cell Malignancies	NA

Data obtained from clinicaltrial.gov (Accessed on 24th March 2024)

### TIL therapy

TIL therapy involves isolating TIL cells from tissue near the tumor, amplifying them in vitro with growth factor IL-2, and then reinfusing them into the patient’s body to expand the immune response and treat primary or metastatic tumors (Fig. 5b).^179^ TIL therapy focuses on identifying T cells that effectively target cancer’s specific mutations. After isolating these T cells, which are capable of specifically recognizing the tumor’s mutated cells, they are utilized to mount a cell-mediated immune response. This response primarily involves the direct destruction of tumor cells through the release of cytotoxins.

TIL therapy offers distinct advantages over other cell therapies. Notably, TILs are derived from tumor tissue, unlike the majority of cell therapies which originate from blood. This difference significantly influences the immune cells’ tumor recognition capabilities: over 60% of TILs can identify tumors, in contrast to less than 0.5% of blood-derived immune cells.^180^ Furthermore, TILs (Tumor-Infiltrating Lymphocytes) exhibit enhanced specificity due to their well-adapted chemokine receptor systems, which facilitate more effective infiltration into tumor tissues. These TILs are capable of selectively targeting tumor-specific T cells, effectively overcoming tumor heterogeneity without engaging normal tissues. Their high degree of tumor specificity enables TILs to identify and precisely target a range of tumor antigens.

TILs therapy is now being investigated as a potential second-line treatment for a number of malignancies, most notably advanced/metastatic melanoma. Concluding from a meta-analysis involving 13 trials (published from 1988 to 2016), TIL+ recombinant IL-2 treatment for metastatic melanoma, excluding uveal melanoma, was found to have an ORR of 41% and an overall complete response rate (CRR) of 12%. The ORR for the high-dose IL-2 group was 43%, while for the low-dose group was 35%.^181^In a phase 2 clinical trial, TIL treatment was effectively used to treat uveal melanoma; 35% (7/20) of patients showed an ORR, with 1 patient obtaining CR and 6 patients achieving PR.^182^ In a recent clinical trial, TIL in combination with high-dose IL-2 treatment for metastatic melanoma demonstrated an 80% disease control rate and a 36% ORR. Surprisingly, in this trial, patients with immunotherapy-refractory melanoma achieved an ORR and DCR of 41% and 81% respectively, suggesting that TIL therapy may offer a viable alternative for treating anti-PD-1 failure.^183^ A multi-center, randomized, Phase 3 clinical trial compared TIL therapy with anti-PD-1 immunotherapy in patients with metastatic melanoma. The results showed that patients receiving TIL therapy had better ORR (49% vs. 21%), longer OS (25.8 months vs. 18.9 months), and longer PFS (7.2 months compared to 3.1 months for ipilimumab).^184^ These findings suggested that for patients with advanced melanoma, TIL treatment may be more effective than immunotherapy. Future research should focus more on the complexity and variability of TIL preparations and assess the efficacy of TIL therapy in combination with immune checkpoint blockades.

### TCR-T

TCR-T is a gene editing technology that introduces the genes of T-cell receptors (TCRs) into the patient’s own T cells. This enables the expression of exogenous TCRs that can specifically recognize tumor antigens and have the activity of killing tumor cells. The TCR is a heterodimer composed of two transmembrane peptide chains, an α chain, and a β chain. Each chain contains a constant region and a variable region. The variable region recognizes and binds to antigen-presenting MHC molecules.^185^ TCRs bind with CD3 to form the TCR-CD3 complex, which activates T cells by recognizing and binding antigens presented by MHC molecules, promoting T-cell proliferation and differentiation^186^ (Fig. 3b).

CAR-T therapy uses artificially designed single-chain antibody fragments (CARs) to recognize antigens only on the surface of tumors and activate T cells through intracellular co-stimulatory molecules. TCR-T therapy, on the other hand, can recognize both surface and internal antigens of tumors, making it more suitable for solid tumor treatment. TCR-T therapy relies on affinity-optimized or naturally occurring TCRs to recognize antigens presented by tumor MHC molecules, transmitting stimulating signals through the TCR-CD3 complex. TCR-T therapy possesses the capability to recognize an extensive repertoire of hundreds or even thousands of intratumoral antigens, rendering it highly potent against solid tumors (Fig. 5c). Moreover, TCR-T cell therapy closely mimics the functionality of endogenous T cells in the human body.

Tumor-associated antigen (TAA), including tissue differentiation antigen (TDA), cancer germline antigen (CGA), and tumor-specific antigen (TSA), which comprise mutation-associated neoantigen, viral antigen and alternative tumor-specific antigens, represent two major classes of targets for TCR-T targets. The treatment with TDA-targeting TCR-T cell therapy has shown disappointing preliminary outcomes in multiple clinical trials, with an ORR of less than 30%.^187^ In a Phase 2 clinical trial using MAGE-A4-targeting TCR-T cell treatment for advanced solid tumors, encouraging findings were reported, including a median OS of 15.4 months, a median PFS of 3.8 months, and an estimated 1-year OS rate of 90%.^188^ Clinical trials continue to evaluate additional therapeutic targets, such as TSA, neoantigen, or viral antigen. Published data demonstrated that 50% of patients in a clinical trial using viral protein HPV-16 E7 TCR-T cell treatment for metastatic HPV-associated cancers had a positive response.^189^ In addition, results from eight ongoing clinical trials investigating the combination of anti-PD-1/anti-PD-L1 and TCR-T cell treatment for metastatic solid tumors are not yet available.

### CAR-M

With the advancement in macrophage-based scientific research, CAR-M has emerged as a new direction in cellular therapy. A key distinction between macrophages and T cells is that macrophages are the first responders in the body’s defense against viral infections. In 2018, Dr. Saar Gill and Dr. Michael Klichinsky, CAR-T cell therapy experts from the University of Pennsylvania, founded Carisma Therapeutics with a focus on developing CAR-M therapy for cancer treatment.^190^ In 2020, they published a paper in the journal Nature Biotechnology introducing a HER2-targeting CAR-M. In a SKOV3 human ovarian cancer mouse model, CAR-M demonstrated effective tumor killing and extended overall survival in mice.^191^ Additionally, CAR-M exhibited the ability to resist TAM-mediated polarization towards M2 macrophages and actively convert them into M1 macrophages.^192^ Macrophages recognize and engulf specific cancer cells through CAR while activating downstream inflammatory pathways. This leads to recruitment and activation of antigen-presenting cells and T cells. The body can maintain a long-lasting memory of the killing process to prevent tumor cell resurgence. At present, CAR-M is developing rapidly. The first-generation macrophage CAR uses TIR to replace CD3ζ in CAR-T, enhancing M1 polarization and immune activation. Second-generation CAR-M connects TIR and CD3ζ, leading to strong immunity and extended M1 activation^193^ (Fig. 5d). However, several technical challenges remain to be addressed. Firstly, a significant limitation is the restricted number of cells that can be procured for use. While T cells and NK cells from patients can be expanded in vitro, macrophages do not proliferate in this setting. Recently, Jin Zhang’s team published their findings in Nature Immunology, demonstrating that CAR-iM can be generated from induced pluripotent stem cells (iPSCs) and effectively eliminate tumors through cytoburial mechanisms.^194–197^ This research offers a potential solution to the issue of cell population limitation. Secondly, like CAR-T, CAR-M may also face challenges such as migration and inhibition within the tumor microenvironment, which need further research and investigation.

Clinical trials of CAR-M treatment are being considered for research involving humans. According to recently published research, three patients with malignant peritoneal mesothelioma and ovarian cancer experienced stable disease following intraperitoneal delivery of mesothelin-targeting CAR-M, which was generated from PBMC.^198^ In another study, three out of four patients (75%) with HER2-overexpressing solid tumors showed good tolerance and response to treatment with another anti-HER2 CAR M (CT-0508), achieving stablilization of their conditions within an eight-week follow-up.^199^ Other two clinical trials NCT05007379 (patients’ organoid derived-CAR-M for breast cancer) and NCT05138458 (PBMC derived-CAR-M for refractory/ relapsed T-cell lymphoma) are still ongoing.

### The comparison between different cell therapy

The cellular therapies mentioned utilize diverse technologies and demonstrate their respective potentials in cancer treatment. CAR-T cell therapy is renowned for its significant efficacy in certain hematological cancers but may induce severe cytokine release syndrome (CRS) and has limited effectiveness against solid tumors; CAR-NK cell therapy is considered a potential alternative due to its lower toxicity and CRS risks, yet its clinical application is still in the early stages; TIL therapy leverages immune cells extracted directly from tumors, offering high tumor specificity, but the preparation process is complex and costly; TCR-T therapy can recognize a broad range of tumor antigens, particularly intracellular antigens, but its development and manufacturing are more complex and clinical data are limited; CAR-M therapy enhances the tumor-engulfing capabilities of macrophages and may improve the tumor microenvironment, but as an emerging therapy, its clinical applications and long-term outcomes still require further research and validation. Overall, these cellular therapies in cancer treatment warrant further exploration in the future, while also facing technical challenges, safety concerns, and cost-effectiveness considerations.

## Genetic therapy

According to the American Society of Gene and Cell Therapy (ASGCT), gene therapy is described as “the introduction, removal, or modification of a person’s genetic code to treat or cure a disease.” This therapeutic approach involves directly introducing genetic material (usually DNA or RNA) into cells to alter genetic information and biological functions. Although it has shown promise in preclinical research and early-phase clinical trials, it still faces many challenges, including ensuring the safety of the treatment, improving the efficiency of gene delivery, and avoiding immune responses.

### mRNA

From May 2005 to March 2024, in total, there are at least 150 clinical studies related to oncology mRNA, including 89 clinical studies on mRNA drugs, 40 studies on mRNA therapies targeting dendritic cells (DCs), 18 studies on neoantigens, two studies on the treatment of HBV-positive HCC, and 1 study on the modulation of immune factors (Supplementary Table 2). Among them, the development of research targeting neoantigens has progressed rapidly since the onset of the COVID-19. Thirteen related clinical trials took place between 2022 and March 2024. Furthermore, 53.9% (48/89) of mRNA drug studies have seen an explosion in activity since the start of COVID-19. These results highlight the fervent pace of mRNA research. mRNA has shown promising efficacy in encoding neoantigens for cancer vaccines, tumor-associated antigens (TAAs) for activating dendritic cells, tumor suppressor factors for inhibiting tumor progression, CAR for engineered T-cell therapies, and genome-editing proteins for gene therapy. We have the details of mRNA vaccines in the following part.

mRNA therapy is an emerging medical technology that uses mRNA-based molecules to treat or prevent diseases. Compared to other therapies, mRNA technology has numerous advantages. It can produce a variety of vaccines and therapeutic drugs in a shorter time frame. The production cycle for mRNA, from In Vitro Transcription (IVT) to the preparation of mRNA-LNP complexes, takes approximately 10 days.^200^ Upon administration into the organism, mRNA does not integrate into the host genome, therefore, the risk of mutations can be avoided. It degrades in the body after a brief period of action, resulting in no long-term toxicity. Moreover, mRNA therapy does not require a viral vector, which also reduces the risk of infection.

However, there are also some disadvantages of the mRNA treatment. First, delivering mRNA molecules to specific cells or tissues presents challenges and requires effective delivery systems, such as lipid nanoparticles (LNPs). Secondly, naked mRNA molecules are less stable in vitro and are susceptible to degradation by RNases, necessitating higher-quality storage and transportation systems. Thirdly, while mRNA therapies are generally considered to have low immunogenicity, in some cases, they may activate the immune system, leading to adverse reactions. Fourthly, targeting tissues other than the liver (which is the primary target for mRNA therapies) requires improved delivery systems. Additionally, the long-term effects and safety of mRNA therapies are not yet fully established.

### ZFNs technology

Genome-editing modalities, including zinc finger nucleases (ZFNs), transcription activator-like effector nucleases (TALENs), and the CRISPR/Cas9 system, enable targeted genome modification in eukaryotic cells. They induce double-strand DNA breaks (DSBs) at specific loci and leverage cellular repair mechanisms, such as non-homologous end joining (NHEJ) or homology-directed repair (HDR), to precisely edit genetic sequences.^201–203^ Moreover, the emergence of Prime Editing has expanded the scope of precise genetic manipulation. These methods have significantly advanced cancer gene identification, tumor cell epigenetic regulation, targeted delivery systems, the development of oncological animal models, and the clinical application of cancer immunotherapy and prophylaxis.^204–206^

**Fig. 6:**
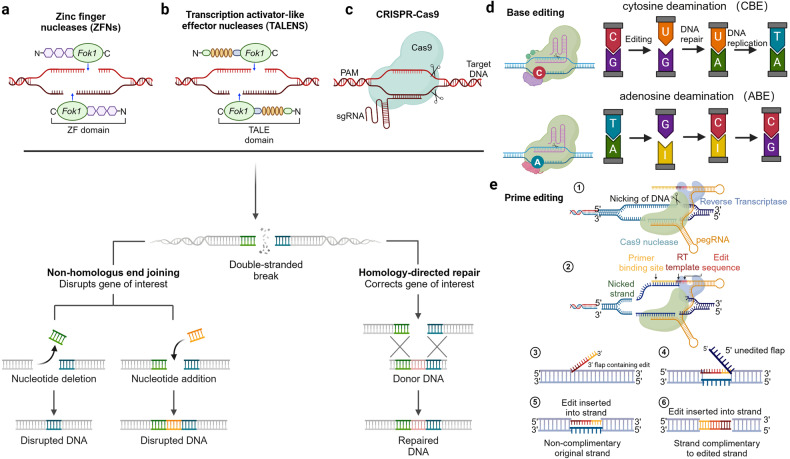
**Various modes of genome editing. a** ZFNs: By designing multiple zinc finger proteins to bind with specific DNA sequences, they guide the nuclease to cut the target gene, achieving precise gene editing through NHEJ and HDR. **b** TALENs: Specific transcription activator-like effector molecules are fused with nuclease domains. Two TALEN modules are necessary to bind to the target site, with a FokI nuclease fused to the DNA-binding domains, enabling precise gene editing through NHEJ and HDR. **c** CRISPR-Cas9: This system employs the Cas9 protein, guided by RNA molecules, to enable precise DNA cleavage at specific target sequences. It achieves accurate gene editing through NHEJ and HDR. **d** Base editing: an advanced form of Cas genome editing that enables the substitution of specific bases without inducing double-stranded breaks. This technique is categorized into two main types: C•G to T•A Base Editors (CBEs) and A•T to G•C Base Editors (ABEs). **e** Prime editing: The procedure involves constructing a prime editor complex, which includes a Cas domain, an RT (reverse transcriptase) domain, and a pegRNA. This complex searches for the DNA segment containing the target mutation and introduces a nick just in front of the mutation site. The nicked DNA strand then serves as a primer for DNA synthesis within the RT complex. The corrected, nicked strand is preferentially used over the original diseased strand, and the cell’s natural DNA repair mechanisms subsequently remove the diseased strand. This figure was created with Biorender.com

Zinc-finger nucleases (ZFNs) represent a sophisticated gene-editing platform that integrates the sequence-specific recognition of zinc finger proteins (ZFPs) with the DNA cleavage function of the FokI nuclease. This technology enables the precise targeting and cleavage of predetermined genomic sequences, thereby triggering the cell’s intrinsic repair machinery to execute site-specific genetic modifications (Fig. 6a).^207^ Predominantly, naturally occurring restriction enzymes recognize palindromic sequences of 4–6 base pairs (bp), whereas a solitary zinc-finger module, comprising approximately 30 amino acids, discerns a 3-bp DNA segment.^208^ By arranging multiple zinc-finger modules in a tandem array, ZFNs can discern sequences ranging from 9 to 18 bp, thereby achieving remarkable specificity within the expansive human genomic landscape, which encompasses approximately 6.8 billion base pairs.^209^ This advancement has been pivotal for the accurate localization of genetic sequences across the human genome.^210^

Furthermore, the strategic pairing of two ZFNs with distinct sequence specificities can facilitate cleavage at non-inverted repeat sites within a genome, provided they are correctly positioned and oriented.^209^ In the realm of cancer therapeutics, ZFNs have been harnessed to modulate both tumor cells and T cells. A clinical trial has documented the efficacy of ZFN-603 and ZFN-758 in treating high-risk human papillomavirus (HPV)-associated cervical intraepithelial neoplasia (CIN).^211,212^ Additionally, the employment of adeno-associated virus serotype 6 (AAV6) as a delivery vector has enabled the efficient correction of CCR5 or AAVS1 in CD8 and CD4 T cells.^213^

Despite ZFNs’ potential in cancer therapy, they encounter several clinical challenges. The synthesis of ZF proteins is less modular than the CRISPR system, necessitating intricate protein engineering for the design and assembly of DNA recognition sequences, which is both technically demanding and costly. Moreover, ZFNs are susceptible to off-target effects and non-specific binding. To mitigate these issues, researchers have engineered single-residue substitutions within the FoKI domain, which maintain on-target activity while diminishing off-target efficiency, exemplified by a 98% reduction in TRAC gene expression in T cells.^201^ However, ZFNs exhibit lower efficiency compared to other gene-editing modalities, such as CRISPR/Cas9. With the advent of CRISPR/Cas9, ZFNs have receded from the forefront of gene therapy for cancer. Nonetheless, ZFNs retain a significant role in specific applications where extreme sequence specificity is paramount, particularly in gene-editing tasks that require high precision.

### TALEN technology

Followed the footstep of ZFNs technology, the TALEN technology was developed in 2010.^214^ TALEN is composed of a non-specific DNA cleavage domain and a sequence-specific DNA-binding domain, which contains a highly conserved repeat sequence from transcription activator-like effector (TALE). Two TALEN modules are required to bind to the target site, and a FokI nuclease is fused to the DNA-binding domains to cleave DNA (Fig. 6b). The DNA binding domain of TALENs is composed of reiterated TALE modules, wherein the 12th and 13th amino acids within each module dictate the precise DNA binding site. By strategically altering these amino acids, TALENs can be engineered to recognize virtually any DNA sequence with high specificity. Also, because the TALENs must bind to adjacent target sites simultaneously to elicit a DSB, this reduces the chance of off-target effects compared to other technologies.

In contrast to ZFNs, which utilize a 30-amino-acid motif to bind three base pairs of DNA, TALENs necessitate 33–35 amino acids to identify a single base pair.^215^ This disparity in size can impede the co-delivery of both TALEN monomers within a single viral vector, particularly when packaging capacity is constrained. Furthermore, the propensity for instability in tandemly repeated sequences, as observed in TALENs, presents challenges for viral vector-based delivery. It is well known that multiple sequence repeats in TALEN genes hampers the use of lentiviral vectors. The lentiviral vectors can efficiently package TALEN-encoding mRNAs, but recombination during reverse transcription impedes successful delivery.^216^ Adenoviral systems are recommended as a valuable TALENs gene delivery platform.^217^

Although TALEN technology is not currently the prevalent method in the application of CAR-T therapy for cancer, it has been employed in specific research initiatives. A salient study from 2015 demonstrated the strategic use of TALENs to inactivate the TCR and CD52 genes within the CAR19-T cells, thereby averting the host’s immune rejection typically encountered in autologous CAR-T therapies and advancing the prospect of allogeneic CAR-T cell transplantation in CAR19 T treatment.^218^ Additionally, there’s a clinical trial about designated TALENs (T27 and T512) targeting HPV16 E6 and E7 to induce cell apoptosis in CIN.^219^ In summary, TALENs offer straightforward design and broad gene-targeting capabilities, but their potential in cancer therapy necessitates further exploration.

### CRISPR therapy

#### CRISPR/Cas9 therapy

Clustered regularly interspaced short palindromic repeats (CRISPR)-CRISPR-associated protein (Cas)9 (CRISPR/Cas9) is a revolutionary gene-editing technology that allows researchers to make precise and efficient edits in the genome of cells. CRISPR-related gene-editing technology is presently regarded as one of the most prominent tools. The most used CRISPR system is the Type II CRISPR-Cas9 system from Streptococcus pyogenes (SpCas9). It involves the utilization of the Cas9 protein, which is directed by RNA molecules, to facilitate precise DNA cleavage at targeted sequences. After the DNA cleavage, the cell’s inherent repair pathways can be harnessed to either incorporate or excise genetic material (Fig. 6c). This CRISPR-based therapeutic intervention holds promise in the treatment of genetic disorders through the rectification of pathogenic mutations and has additionally been investigated as a prospective therapeutic strategy for malignancies.

CRISPR has become a highly refined technology for gene editing in ex vivo adoptive T-cell therapies. Numerous clinical studies have employed CRISPR to eliminate PD1 expression on T cells to enhance their anti-cancer properties.^220–225^ But since this technology is still very new, only one clinical trial (NCT02793856)—a dose-escalation study of ex vivo knocked-out, expanded, and selected PD-1 knockout T cells from autologous origin—has been completed. Others are ongoing. One ongoing clinical trial (NCT03545815) evaluates the feasibility and safety of CRISPR-Cas9 mediated PD-1 and TCR gene-knocked out chimeric antigen receptor (CAR) T cells in phase 1 patients with mesothelin-positive multiple solid tumors. Another ongoing trial (NCT03081715) plans for 16 advanced esophageal cancer patients to receive two cycles of CRISPR knockout PD-1 engineered T cell infusion. Knocking out HLA in allogeneic T cells to reduce immune rejection is also a common procedural approach.^226^ A clinical study initiated on March 20, 2024 (NCT06321289), employs the CRISPR-Cas9 gene-editing tool to simultaneously knock out the endogenous receptor α constant (TRAC), human leukocyte antigen (HLA)-A/B, CIITA, and programmed death 1 (PD-1) genes in T cells from healthy donors to observe the reduction in GvHD toxicity with this strategy. Additionally, CRISPR-Cas9 serves as a mainstream technology for synthetic lethal screening of drugs, aiding in the development of anticancer drugs. Current exploration areas mainly include intrinsic cellular mechanisms, such as interactions between BRCA-PARP^227^ and BRAF-EGFR.^228^

However, applying CRISPR directly in the body raises several key issues, including precise targeting to avoid unintended effects, preventing unnecessary immune reactions, and ensuring the long-term efficacy and stability of the treatment. It is crucial to select an appropriate in vivo delivery system. Clinical trials using AAVs as a delivery system for CRISPR in vivo cancer treatment in patients have not yet been conducted. However, preclinical studies have shown a wealth of potential applications in brain tumors,^229,230^ lung cancer,^231–233^, and liver cancer.^234–236^

#### Base editing

Base editing is a modified Cas genome editing method that can replace specific bases without any double-stranded breaks. It is split into two classes: C•G to T•A Base Editors (CBEs) and A•T to G•C Base Editors^237^ (ABEs). CBEs consist of 3 components: Cas9n for DNA locus binding, a cytidine deaminase enzyme to transform cytosine to uracil, and an Uracil Glycosylase Inhibitor (UGI) to protect the uracil intermediate from Uracil DNA Glycosylase^237,238^ (UDG). ABEs consist of Cas9n and an adenosine deaminase.^237,239^ The Cas9n first binds to the target locus, allowing the cytidine deaminase or adenosine deaminase to deaminate C or A respectively, nicking the top strand, and allowing the cell’s indigenous DNA repair systems tothe DNA to repair the damage and edit the DNA to the correct base^237,239^ (Fig. 6d).

The fusion of rat APOBEC1 (rAPOBEC1) and Cas9 constitutes the earliest version of the cytosine base editor (BE1). Subsequently, by introducing UGI and modifying the activity of Cas9, researchers have developed more efficient versions of CBE, such as BE2, BE3, and BE4. Improved Cas9 variants such as eCas9, Cas9-HF are used to decrease off-target base editing^237,240^; BE3-Gam and BE4-Gam, where the Gam protein protects DSBs in DNA, are used to reduce accidental locus-based indels and increase product purity, and many other variants are produced.^241^

Base editing can be used in a cancer therapeutic setting by improving CAR-T technology or direct base editing. Studies have shown that autologous CAR-T cells may be produced through multiplex gene KO using base editors when combined with cell sorting. A current limitation of CAR-T therapy is the cost of allogenic CAR-T cells being used. The use of autologous CAR-T cells would require KO of MHC I and II, which can be done much more efficiently with BE combined with cell sorting.^242^ Alternatively, one study by Sayed et al. shows the potential of modifying genetic mutations in cancer cells using base editing by correcting KRAS and TP53 mutations on cancer organoids.^243^

#### Prime editing

Prime editing is a new modified Cas genome editing method that is able to search and replace the targeted gene without the need for double-strand breaks or donor DNA.^244^ The procedure works by constructing a Prime Editor complex (PE) with a Cas nickase domain and Reverse Transcriptase (RT) domain along with a pegRNA, which will have its search domain and replace domain on the same strand,^244^ with a spacer on the 5’ end and the primer binding site and edit template on the 3’ end. The Prime Editor complex searches for the DNA with the target mutation and nicks the strand just in front of the mutation.^244^The nicked DNA strand then primes for DNA synthesis in the RT complex.^244^ The corrected nicked strand is preferentially preferred over the diseased original strand, and the cell’s indigenous DNA repair mechanisms will excise the diseased strand.^244^ The PE will search for the protospacer adjacent motif (PAM) and align the photospacer with the spacer on the pegRNA.^244^ The PAM strand will be nicked and the 3’ side of the pegRNA will hybridize with the PAM strand, allowing the RT to polymerize edited DNA on the target site.^244^ The PE will then leave the strand, leaving the edited DNA with a 3’ flap containing the edit.^244^ The 3’ flap replace the original unedited strand, leaving the unedited 5’ flap to be removed.^244^ The mismatched DNA strand is then repaired to incorporate the edit^244^ (Fig. 6e).

The advantages of prime editing are multifold. It is capable of executing all different types of single-base substitutions, multiple-base substitutions, and small indels.^245^ It also has high editing and on-target precision, above contemporary CRISPR-Cas9 and other base editing methods.^245^ However, prime editing also involves many complicated steps, and despite having fewer moving parts than the other methods. Hence, many of the first developmental efforts involved improvements to the PE system. The first version of PE, hereby called PE1, have a low efficiency, and subsequent versions developed incorporated improved RT domains (PE2),^244^ optimized pegRNAs(PE3, G-PE, sPE, ePE),^244,246^ improved nicking strategy (PE3, PE3b),^244^ the addition of transient expression of a dominant MMR protein (MLH1dn) (PE4,PE5),^247^ addition of the sgRNA for nicking, enhanced nuclear localization (PE*, PEmax),^246,247^ and many other modifications. A possible drawback of the PE system may include genotoxicity to hematopoietic stem cells, according to Fiumara et al., that while the PE system have a higher accuracy that Cas9 and Base Editing, PE can still induce DNA DSBs and deletions at the target site.^248^

Prime editing is a versatile tool with many potential applications in cancer modelling and therapy. A study by Liu et al. has demonstrated the use of prime editing in cancer modeling by increasing tumor burden through somatic engineering, mutating the β-catenin gene with PE2* to increase tumor formation rates.^249^ A recent study by Davis et al. has confirmed that prime editing can be delivered and successfully executed in the mouse brain, liver, and heart using dual AAV delivery, suggesting a possible therapy for hard-to-reach areas;^250^ meanwhile, a study by Abuhamad et al. is able to revert TP53 mutation using prime editing breast cancer cells.^251^ A study by Jang et al. has similarly been able to correct oncogenic G12 and G13 KRAS mutations with prime editors in HEK293T/17 and human cancer cells in vitro.^252^ Currently, Prime Medicine, a start-up created by the original inventors of prime editing A. Anzalone and D. Li, had made some headway by multiplexing Prime Editing with PASSIGE, creating CAR-T cells by non-viral one-step delivery with high efficiency.^253^

### Delivery system

Currently, adeno-associated viruses (AAV) and lipid nanoparticles (LNP) are among the most advanced delivery systems in clinical use.

#### AAV delivery system

AAV, a single-stranded DNA virus without an envelope, has a genome length of approximately 4.7 kilobases (kb) and features two T-shaped inverted terminal repeats (ITRs). The genome encodes multiple proteins, including Rep and Cap proteins, which play roles in viral replication, assembly, and packaging. Different AAV subtypes have different tissue specificity. Selecting appropriate AAVs can achieve efficient gene delivery in specific tissues, thereby providing more precise and effective means for gene therapy. For example, the AAV9 subtype has a higher affinity for the heart and liver, while the AAV2 subtype exhibits greater selectivity for retinal cells.^254^ AAVs are widely present in humans and animals and is generally considered non-pathogenic. It has become the preferred delivery vector for gene therapy due to its broad host range, low immunogenicity, low cytotoxicity, multiple serotypes, and low frequency of integration into the host genome. However, there’s some limitations of the AAV delivery system. First of all, AAV’s limited packaging capacity and gene length restriction restrict its ability to carry payloads larger than 5 kb such as the SpCas9 protein. To overcome this problem, smaller CRISPR constructs, such as SaCas9, Nme2Cas9, Cas12f, Cas12j, sgRNA, etc., were used.^255^ Also, the approach to split the payload into two separate constructs allowed for more efficient packaging and delivery using viral vectors such as AAVs. Another strategy involves the use of overlapping AAV vectors, which can enhance the recombination efficiency and encourage natural homologous recombination (HR) between the vectors. Additionally, trans-splicing vectors are utilized to achieve precise and controlled gene expression. These vectors contain a splicing donor sequence that can be spliced into the target gene, leading to the production of a functional protein. Split inteins, on the other hand, are used to facilitate the splicing of two protein fragments, allowing for the reconstitution of a functional protein. Another concern with AAVs treatment is its potential impact on the liver. Reports indicate that AAV vectors have a high affinity for the liver.^256^ When targeting the liver, a lower dose is required, whereas higher doses are needed to treat other tissues. However, clinical trials with higher doses of AAVs have raised the risk of liver-related side effects. Several children died from liver function abnormalities after receiving high doses of AT132 (3×10^14 vg/kg in 2020; 1.3×10^14 vg/kg in 2021).^257^ In 2022, Novartis reported that two children died from acute liver failure after treatment with Zolgensma.^258^ Five Airedale terriers with hemophilia A developed cellular proliferation in the liver after receiving AAV8 delivery of the FVII gene.^259^ These issues have sparked controversy surrounding AAV therapy. Reducing the dosage of AAVs is a reliable measure to lower the risk. Sharif Tabebordbar et al. found that MyoAAV can be used for treatment with smaller doses, especially MyoAAV2A-CK8 for mouse treatment, which is 100 times lower than the dose used in the AT132 study.^260^ ASC Therapeutics has enhanced the secretion efficiency of therapeutic proteins by special gene modifications and optimization of the cellular unfolded protein response (UPR), thereby reducing the required dose of AAV in gene therapy.^261^ These approaches offer innovative ways to optimize the delivery and expression of therapeutic genes, providing researchers with tools to fine-tune gene expression and enhance the efficacy of gene therapies.

#### LNP delivery system

Lipid nanoparticle (LNP) systems have shown promise in the delivery of CRISPER therapeutics. These systems are composed of four main lipid components: ionizable lipids, helper lipids, cholesterol, and polyethylene glycol (PEG)-ylated lipids. These components form the structure that encapsulates and protects mRNA molecules, enabling them to resist degradation in the body and effectively deliver to target cells through the bloodstream. Compared to viral vectors, LNPs have numerous advantages. First, they have strong design flexibility because they are composed of four lipids, and the ratio of different lipids can be adjusted to achieve various effects. Additionally, they can anchor other ligands onto their own carriers. For example, tumor-specific antigens (TSAs) or hydrophobic antibody derivatives can be anchored on the surface of functionalized LNPs to enhance their targeting capabilities.^262^ Second, as a non-viral carrier, LNP generally has better safety and lower immunogenicity compared to viruses. Moreover, LNPs possess an innate adjuvant effect, which can promote CD4 + T helper 1 (TH1) mediated anti-cancer responses.^263^

For its disadvantage, the current side effects of LNPs mainly fall into three categories: allergic reactions, immune responses, and liver effects. At the same time, due to capacity limitations, LNPs have certain constraints when delivering complex genes and base editing. At present, the issue of liver burden can be addressed by optimizing the ionizable lipid and reducing the dosage. The percentage of PEG-lipids, cholesterol, and helper lipids, such as 1,2-distearoyl-sn-glycero-3-phosphocholine, can be adjusted to enhance its stability and reduce toxicity.^264^

LNP, as a delivery system for mRNA vaccines, has been successfully used for prophylactic vaccines against pathogens and is being tested in clinical trials in oncology The targeting efficiency of different LNPs in combination with sgRNA within various tissues in patients needs additional research. One preclinical data has demonstrated that targeted sgPLK1-cLNPs significantly enhance survival rates, achieving an 80% improvement in ovarian tumor mice models.^265^ The other two target PLK1 in A375 tumor-bearing mice^266^ and HepG2 tumor bearing mice^267^ didn’t show sufficient response. The application of LNPs for the delivery of CRISPR in cancer therapy is still in its early stages and requires further research and clinical trials to validate its safety and efficacy.

#### PNPs delivery system

In addition to lipid nanoparticles, polymer nanoparticles (PNPs) are another promising delivery system for gene therapeutics. PNPs are widely used as drug delivery systems, offering controlled release of drugs, enhancing the solubility of poorly soluble drugs, and targeted delivery to tissues or cells. A critical advantage of PNPs is similar to LNPs for customization and integration of diverse physicochemical characteristics to enhance their effectiveness. Moreover, PNPs are easy to be engineered as carriers, creating drug delivery systems that are both non-toxic and biocompatible. Several studies have been conducted on the use of PNPs as carriers for CRISPR in cancer applications. One study reported that they successfully established a co-delivery PNPs system composed of sg-VEGFR2 and Cas9, which led to more than 70% suppression of HepG2-induced HCC tumor progression.^268^ Additionally, Wu et al. utilized an sgHMGA2 PNPs system to achieve high-efficiency delivery (95%) and editing (82%) in gastric cancer.^269^ This system was shown to decrease immune responses in mice that actively targeted breast cancer tumor cells, aiding in the prevention of tumor re-growth upon re-challenge. Moreover, this approach was found to induce immunological memory, conferring resistance to lung metastatic spread.^270^ As the application of PNPs in gene editing is still in its infancy, no adverse events have been reported to date.

### Challenges to gene editors

In the development of gene therapies, simplicity of design is important, but it must be balanced with the complexity of achieving tropism and avoiding off-target effects. Long-term nuclease expression requires longer safety studies due to mutations, and in vivo characterization is necessary to account for species differences between NHPs, mice, and humans.^263^ Delivering therapies to organs other than the liver requires further study, and the limitations of different delivery systems must also be considered.

## Neoantigen and cancer vaccine

Genomic instability and mutations are fundamental characteristics of tumorigenesis, enabling the hallmark capabilities of cancer, including the maintenance of proliferative signaling and resistance to cell death.^271^ Concurrently, mutations can generate aberrant peptides that are presented by the major histocompatibility complex (MHC), which may activate anti-tumor adaptive immunity by lymphocytes.^272,273^ This process underlies the principle of neoantigens and cancer vaccines. Given the unique expression of neoantigens in tumor cells, they are regarded as an ideal target for tumor vaccines.

### Neoantigen identification and validation

**Fig. 7:**
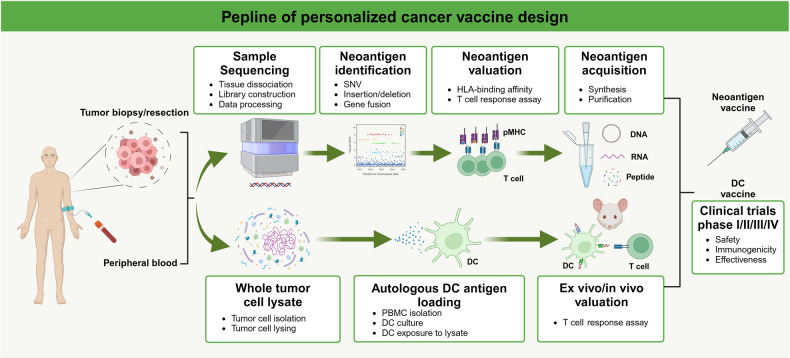
**The process of cancer vaccine development**. The preparation of cancer vaccines is a meticulous process involving multiple steps, aimed at developing vaccines that can stimulate the immune system to target cancer cells. This process begins with the selection and identification of specific tumor antigens, followed by the design of the vaccine, which may include peptides, recombinant proteins, genetic vectors, or dendritic cells. Subsequently, the vaccine undergoes immunological testing in vitro and is evaluated for its safety and efficacy in animal models. After successfully passing preclinical research, the vaccine proceeds to human clinical trials, including Phase I, II, and III trials, to assess its safety, immunogenicity, and therapeutic effectiveness. This figure was created with Biorender.com

Genetic alterations in cancer may result from single nucleotide variants, insertions, deletions, gene fusions, and oncogenic virus infections.^274–277^ Thanks to the development of multi-omics technologies, including genome sequencing and mass spectrometry for the proteome, it is now possible to efficiently screen for potential neoantigens in a large pool. Through next-generation sequencing data of tumor genomes or transcriptomes compared to normal ones, neoantigens can be preliminarily predicted in silico.^278^ Subsequently, computational algorithms that analyze the binding affinity between MHC molecules and the predicted neoantigens/peptides are used to select optimal candidates.^279,280^ Following bioinformatic prediction, experimental validation should be conducted to assess the TCR recognition of the peptide-MHC (pMHC) complexes using T cells derived from patients or healthy donors^281,282^ (Fig. 7). However, reports indicate that only a few bioinformatically predicted neoantigens/peptides are recognized by T cells,^281^ leading to controversy regarding the reliability of these predictions. An alternative approach to obtain immunogenic neoantigens/peptides involves loading autologous antigen-presenting cells (APCs), such as dendritic cells (DCs), with tumor lysates and validating them ex vivo by co-culturing the lysate-pulsed DCs with T cells.^283^

### Vaccination

After screening and validation, immunogenic neoantigens can be used in various forms of vaccines, including peptides, nucleic acid-based vaccines, and lysate-pulsed dendritic cells (DCs).

#### Peptide vaccines

Peptides are the most common form of cancer vaccines, consisting of either a single synthetic peptide or a mixture of several synthetic peptides.^284–286^ Clinical trials have confirmed that personalized peptide-based vaccines can induce tumor-specific immunity and long-term immune memory across various cancer types.^287–289^ To enhance the immune response, immunoadjuvants are often used in conjunction with peptide-based vaccines,^290,291^ as will be discussed in detail in the relevant section.

#### mRNA vaccines

mRNA vaccines have proven to be an efficient and safe therapeutic method during the COVID-19 pandemic, indicating a promising future in cancer immunotherapy. Compared to peptide-based vaccines, mRNA vaccines can encode full-length neoantigens or carry multiple neoantigens on a single mRNA molecule.^292^ To ensure stability and intracellular delivery, nanoparticles, such as lipids, are often used to facilitate the transport of mRNA vaccines.^293^ Several mRNA cancer vaccines have recently completed Phase I/II trials. Autogene cevumeran, an mRNA vaccine containing 20 personalized neoantigens delivered by lipoplex nanoparticles, showed T-cell expansion in 8 out of 16 pancreatic cancer patients in a Phase I study, with benefits in recurrence-free survival (RFS) after vaccination.^294^ Another personalized mRNA vaccine, mRNA-4650, contains 20 neoantigens based on sequencing data from individual patients. A Phase I/II study involving four patients with metastatic gastrointestinal cancer who received mRNA-4650 vaccination showed no objective clinical responses, although the vaccine elicited T-cell responses against predicted neoepitopes.^295^ Therefore, enhancing the immunogenicity, stability, and delivery methods of mRNA vaccines should be considered.

#### DNA vaccines

Similar to mRNA-based cancer vaccines, DNA vaccines are constructed using an expression vector, such as a plasmid, to encode the neoantigens identified through sequencing,^296^ which are more stable and easier to store and transport. Due to these advantages, DNA vaccines are increasingly applied in clinical practice. In 2014, a Phase I study focusing on a plasmid-based mammaglobin-A (MAM-A) DNA vaccine reported that the vaccine was efficient in eliciting cytotoxic antigen-specific T cells, and vaccinated breast cancer patients experienced prolonged PFS compared to those who did not receive vaccination due to HLA-screening failure.^297^ Another plasmid-based DNA vaccine targeting insulin-like growth factor binding protein-2 (IGFBP-2) also presented an activation effect on the T helper-1 immune response in ovarian cancer patients, as both IFNγ-secreting T cells and T-bet positive T cells were increased after immunization.^298^ A double-blind, placebo-controlled clinical trial targeting human papillomavirus (HPV) proteins in patients with cervical intraepithelial neoplasia (CIN) provided more compelling evidence of the therapeutic effects of DNA vaccines.^299^ Furthermore, one Phase IIb study aimed to assess VGX-3100, a double plasmid vaccine encoding HPV protein E6 and E7. After 36 weeks of follow-up, the result showed a significant difference in histopathological regression between the two groups (49.5% of the VGX-3100 group (107 patients) and 30.6% (36 patients) of the placebo group), indicating the potential of VGX-3100 as a non-surgical treatment option for patients with CIN2/3.

#### Dendritic vaccines

Since the dendritic cell is the key antigen-presenting cell to stimulate adaptive immunity,^300^ neoantigen-loaded dendritic cells (DCs) provide a direct way to initiate anti-tumor immunity.^301^ Whole-tumor lysate is the most used method for neoantigen loading. DCVax-L, an autologous whole-tumor lysate-loaded dendritic cell vaccine, demonstrated improved survival both in patients with newly diagnosed and recurrent glioblastoma.^302^ Additionally, synthetic peptides, based on sequencing and prediction analysis, can be used for antigen loading. A pilot study explored the efficacy of the personalized peptide-loaded dendritic cell vaccine, Neo-DCVac, in patients with advanced lung cancer.^283^ The T-cell response assay verified a strong increase in IFNγ secretion and cytotoxicity, thereby strengthening the efficiency of tumor therapy.

### The advantages, disadvantages, and challenges of cancer vaccine

Unlike other types of drugs, cancer vaccines are preventive in nature. Therefore, their first advantage is that they can effectively suppress the occurrence of tumors. In addition, research has shown that cancer vaccines have the potential to stimulate long-term immune memory, providing patients with lasting protection and reducing the risk of cancer recurrence. However, the main disadvantage of cancer vaccines is based on the issue of neoantigens. Due to the individual variability of antigens and the interference of the immune microenvironment, the efficacy of cancer vaccines targeting neoantigens exhibits inconsistencies.

Though great success has been achieved, it remains a pressing issue to be addressed in cancer vaccine. The most worrisome problem is the immunogenicity of the vaccine, as a considerable part of patients still showed no response to various cancer vaccines. One practical method is to add adjuvant along with vaccines to enhance innate immune response. Improving the form or sequence of synthetic peptides to enhance their efficiency of binding to MHC is another solution that could be taken into consideration.

## Oncolytic virus

The history of oncolytic viruses (OVs) can be traced back to the early 20th century. An Italian woman suffering from cervical cancer was bitten by a dog after being injected with a rabies virus vaccine. Miraculously, the tumor disappeared, and the woman enjoyed a cancer-free survival for 8 years.^303^ Though the underlying mechanisms of this phenomenon were uncertain at that time, physicians began to use rabies virus vaccines to treat patients with cervical cancer. A hundred years have passed, and OVs therapy has come into a new era, achieving significant success in treating a broad range of tumor types.

### Oncolytic mechanisms

Oncolytic viruses can kill tumor cells through several direct and indirect mechanisms according to the design concepts. OVs tend to recognize surface markers that are abnormally overexpressed on cancer cells and then enter those targeted cells. The recognition pattern depends on the virus types and modification strategies. CD46 is often upregulated on tumor cells from leukemia, gastrointestinal cancers, gynecological cancers, and lung cancer,^304^ which can be recognized by measles viruses and some subtypes of adenoviruses.^305^ The most commonly used vector, herpes simplex viruses, utilize the herpesvirus entry mediator (HVEM) and nectin-1 to enter tumor cells.^306,307^

Normal cells will induce the interferon pathway to limit viral replication after infection, while tumor cells are often deficient in this defensive mechanism.^308,309^ Therefore, after entering tumor cells, OVs begin to rapidly replicate, finally leading to tumor cell lysis and releasing new viruses to infect surrounding tumor cells.^310^ Accompanying cell lysis, damage-associated molecular patterns (DAMPs) and pathogen-associated molecular patterns (PAMPs), along with tumor antigens, are released into the tumor microenvironment (TME) and are subsequently sensed by antigen-presenting cells (APCs), including dendritic cells (DCs) and macrophages.^311–313^ Once sensing these signals, APCs will stimulate T cells to induce adaptive immunity and tumor-specific cytotoxicity, thus triggering a systematic anti-tumor immune response.^314^

Besides the common mechanisms, OVs can induce oncolytic effects through different modification methods, which will be discussed in the following parts.

### Clinic application

Despite the long history of oncolytic viruses (OVs), most of the products are in clinical trial stages. Studies on OVs targeting other malignancies, including multiple myeloma, pancreatic cancer, lung cancer, and pediatric cancers, are ongoing, with details provided in Table 5.

**Table 5:** Summary of OVs under clinical investigation for the treatment of cancer

Name	Vector	Insertion gene	Deletion gene	Phase	malignancies	Ref
T-VEC (Talimogene Laherparepvec)	HSV-1	human GM-CSF	ICP34.5, ICP47	I/II/III	melanoma, breast cancer, sarcoma, head and neck, colorectal cancer	^315,317–319^
G47Δ	HSV-1		α47	I/II	glioma	^20,324^
CAN-3110	HSV-1			I	glioma	^471^
OrienX010	HSV-1	human GM-CSF		I	melanoma	^472^
OH2	HSV-2	human GM-CSF	ICP34.5, ICP47	I/II	esophageal cancer, colorectal cancer, gastric cancer, melanoma, head and neck cancer, cholangiocarcinoma, ovarian cancer, breast cancer	^336^
DNX-2401 (tasadenoturev)	adenovirus	RGD motif	E1A	I/II	glioma	^321,322,473^
CRAd-S-pk7	adenovirus	human survivin promoter		I	glioma	^338^
VCN-01	adenovirus	E2F1 promoter	E1A	I	retinoblastoma, pancreatic cancer	^334,474^
Telomelysin (OBP-301)	adenovirus	human TERT promoter, IRES		I	liver cancer, esophageal cancer, head and neck cancer, lung cancer, neuroendocrine cancer, melanoma, leiomyosarcoma, sarcoma, basal cell carcinoma, salivary gland tumor, breast cancer	^475–477^
ICOVIR-5	adenovirus	E2F1 promoter	E1A	I	melanoma, medulloblastoma, astrocytoma, neuroblastoma, thyroid cancer, thymoma, lung cancer, colorectal cancer, sarcoma	^339^
enadenotucirev	adenovirus			I	ovarian cancer, colorectal cancer, head and cancer, urothelial cancer, renal cancer, lung cancer	^478–480^
ONCOS-102	adenovirus	human GM-CSF	E1A	I/II	pleural mesothelioma, ovarian cancer, colorectal cancer, liver cancer, endometrial cancer, sarcoma, breast cancer	^330^
MV-NIS	measles virus	human NIS		I	multiple myeloma	^481,482^
MV-CEA	measles virus	human CEA		I/II	glioma, ovarian cancer	^483,484^
V937 (CVA21)	coxsackievirus			I/II	melanoma, lung cancer, urothelial cancer, prostate cancer	^325,485,486^
ParvOryx	parvovirus			I/II	pancreatic cancer, glioma	^487–489^
PVSRIPO (Lerapolturev)	poliovirus	IRES		I	melanoma, glioma	^490–492^
REOLYSIN (Pelareorep)	reovirus			I/II	esophageal cancer, prostate cancer, melanoma, pancreatic cancer, breast cancer, stomach cancer, lung cancer, liver cancer, mesothelioma, ovarian cancer, tubal cancer, peritoneal cancer, multiple myeloma	^326,493–497^
NTX-010 (SVV-001)	Seneca Valley virus			I/II	small cell lung cancer, carcinoid tumor, adrenal cortical carcinoma, embryonal rhabdomyosarcoma, alveolar rhabdomyosarcoma, Wilms’ tumor, ganglioneuroblastoma, neuroblastoma	^329,498^
JX-594 (Pexa-Vec)	vaccinia virus	human GM-CSF	thymidine kinase (TK)	I/II	sarcoma, liver cancer, melanoma, colorectal cancer, pancreatic cancer, bladder cancer	^331,335,499,500^

Data obtained from clinicaltrial.gov

Talimogene laherparepvec (T-VEC) is the first US FDA-approved OV for patients with recurrent unresectable melanoma, improving the median overall survival to 23.3 months.^315^ It is derived from herpes simplex virus type 1 (HSV-1) and features an insertion of the human granulocyte macrophage colony-stimulating factor (GM-CSF) gene. This insertion enables local expression of GM-CSF at the tumor site, recruiting antigen-presenting cells (APCs) such as dendritic cells (DCs) and inducing downstream T-cell activation.^316^ In addition to melanoma, T-VEC’s effects on breast cancer, sarcoma, and head and neck squamous cell carcinoma are being evaluated in clinical trials, with initial results reported.^317–319^

DNX-2401 is an adenovirus-based OV with a deletion in the E1A gene and an insertion of the RGD sequence, which confers glioma cell selectivity via integrins αvβ3 and αvβ5.^320^ A phase I study confirmed the efficacy of DNX-2401 in patients with recurrent malignant glioma, noting increased CD8 T-cell infiltration in tumors. Among the participants, 3 out of 20 achieved complete responses and experienced a progress-free survival (PFS) of over 3 years.^321^ Additionally, a recent study focusing on the combination of DNX-2401 with an immune checkpoint inhibitor has shown initial results indicating a survival benefit in select patients.^322^

Another OV targeting glioma, G47Δ, is a third-generation engineered HSV-1. It is constructed by deleting the α47 gene and overlapping the US11 promoter from its parental vector, thereby enhancing anti-tumor immunity and reducing replication in normal cells.^323^ Recent phase I/II studies have shown that G47Δ demonstrates good safety and provides survival benefits for patients with advanced glioma.^20,324^

### The advantage, disadvantage, and challenges for OVs

OVs offer numerous advantages. Firstly, they can selectively replicate within tumor cells while also activating the immune system, enabling them not only to directly kill cancer cells but also to serve as vectors for delivering therapeutic genes. Secondly, due to their inherent defects, tumor cells are more susceptible to viral infections, while normal cells are relatively safe as they possess mechanisms to clear viruses. Moreover, the small size of viral genomes and mature genetic engineering techniques make the efficient and cost-effective modification of oncolytic viruses possible, facilitating the specific targeting of cancer cells. Lastly, the multifaceted oncolytic effects of these viruses, include increasing tumor antigen exposure, modulating the tumor microenvironment, and activating the immune system. As the development of ICIs in tumor therapy, the combination of OVs with ICIs is gaining more attention.^325–328^ It is practical to find molecular markers to predict the population who would benefit from combination therapy through multi-omics technology.

When it comes to the drawbacks of oncolytic virus therapy, there are several key issues. First, there is the issue of targeting specificity. Although oncolytic viruses are selective, their specificity in targeting tumors needs improvement to ensure effective infection of the intended cancer cells. Second, there is the issue of immune response. Patients may develop an immune reaction to the oncolytic viruses, potentially limiting the treatment’s efficacy and the possibility of its repeated use. Third, determining the appropriate viral dosage for treatment is challenging. Additionally, controlling the effective propagation of the virus within target cells presents difficulties.

Though OVs have shown efficacy in a wide range of malignancies, there remains many challenges to be faced. One is the treatment-related adverse events including some severe reactions such as lymphopenia, and thrombocytopenia,^20,329^, and combination with other therapy may increase the risk.^330^ It is currently uncertain how OVs caused those adverse events, perhaps related to virus load and tumor necrosis.^331^ Several measures have been taken to reduce the virulence and risks of adverse events. The most utilized method is insertion or deletion of some specific genes in OVs to limit its replication and infection to normal tissues. For example, retinoblastoma, a common pediatric cancer, often results from retinoblastoma 1 gene (RB1) dysfunction, causing an increase in the abundance of E2F.^332,333^ According to this genetic change, VCN-01 was constructed by inserting a E2F-responsive element, E2F1 promoter, to enhance the expression of E1A in E2F-sufficient cells while low replication capability in normal cells.^334^ Another gene-modified OVs, JX-594, was deficient in thymidine kinase (TK) gene and showed a selectivity to tumors with elevated cellular TK abundance.^335^ Thus, genetic modifications to control the expression of virulence-related or add a switch for replication will be the solution to adverse events.

Another issue that needs to be improved is the administration or delivery way of OVs, since most of the OVs are administrated through intratumoral or intracavitary injection to achieve local concentration, which is easy to perform in some superficial or abdominal cancers.^317,336,337^ However, malignancies such as glioma are hard to reach due to their anatomical location, which sets obstacles for OVs administration. To address this issue, some new delivery systems were applied. Adenovirus-based CRAd-S-pk7 was loaded by neural stem cell line to enhance its ability to target glioma and migration across the blood-brain barrier, though the injection of viruses was taken after surgical resection.^338^ Another study loaded the virus, Icovir-5, with autologous, bone marrow-derived mesenchymal stem cells (MSCs) and administrated it through intravenous injection upon the tumor-homing effect of MSCs.^339^ From this perspective, human-derived cells with specific tissue tropism could act as an ideal carrier to take OVs to tumor site.

## Immunologic adjuvant and innate immunity activator

Although current cancer immunotherapies primarily focus on enhancing the adaptive immune response, such as immune checkpoint inhibitors to promote T-cell cytotoxicity,^340^ methods aimed at activating the innate immunity within tumors are gaining more attention. In 1890, the American surgeon William Coley accidentally discovered that a patient with both sarcoma and erysipelas experienced spontaneous tumor regression. This observation encouraged him to use the mixed toxins from Streptococcus erysipelas and Bacillus prodigiosus, known as Coley’s toxins, to treat patients with sarcoma.^341^ Although some patients experienced regression after receiving Coley’s toxins, skepticism about Coley and his toxins has always persisted.^342^ Now, it has become clear that the bacterial components may activate the innate immunity and induce an anti-tumor effect.^204^ Therefore, therapies such as Coley’s toxins and immunologic adjuvants, which aim to stimulate the innate immune system, could represent a novel approach to treating cancer.^343^

### Bacillus Calmette-Guérin (BCG)

Despite the fact that intravesical administration of BCG in bladder cancer patients has long been the standard care, backed by solid evidence from clinical practice,^344^ the underlying mechanisms are not yet fully understood. BCG administration can induce the secretion of proinflammatory cytokines from tumor cells and promote immune cell infiltration.^345^ It is reported that NK cells can be activated by BCG, potentially playing a significant role in the tumoricidal process.^346^ In addition to stimulating the innate immune response, BCG can also elicit an adaptive immune response against bladder cancer. This may be a consequence of increased antigen-presenting efficiency following the activation of innate immune cells.^347^ In summary, further efforts should be dedicated to elucidating the immunotherapeutic effects of BCG, which could provide valuable insights for developing novel treatment strategies for other cancer types.

### Toll-like receptors (TLRs) agonists

The family of Toll-like receptors (TLRs) comprises several members that are key regulators of innate immunity and initially garnered attention for their role in infections.^348^ The activation of various TLRs in antigen-presenting cells, such as macrophages and dendritic cells, can induce the secretion of proinflammatory cytokines and subsequently stimulate adaptive immunity in T cells.^349^

The TLR3 agonist, poly-ICLC, is frequently used as an adjuvant with cancer vaccines and has demonstrated good safety and efficacy.^350,351^ Similarly, lipopolysaccharide, a major component of Gram-negative bacteria, is the natural ligand for TLR4 and can also be employed as an adjuvant in cancer vaccines.^352^

In addition to being combined with cancer vaccines, TLR agonists can be administered as monotherapy or neoadjuvant therapy for certain malignancies. A phase I study investigated the efficacy and safety of G100, a synthetic TLR4 agonist, in patients with Merkel Cell Carcinoma.^353^ Following intratumoral injection, G100 significantly increased the infiltration of T cells, activated the immune response, and led to sustained tumor regression in patients. Motolimod, a TLR8 agonist, when combined with chemotherapy and cetuximab, achieved improved outcomes compared to placebo in patients with HPV-positive head and neck squamous cell carcinoma.^354^

### The advantage, disadvantage and challenge of Immunologic adjuvant and innate immunity activator

Adjuvants and innate immunity activators serve as essential tools in enhancing vaccine efficacy and activating the immune system. Adjuvants enhance the protective power of vaccines by strengthening immune responses in a non-specific manner. They reduce the necessary amount of antigen and meet the immune needs of diverse populations. However, their use is accompanied by safety concerns, including local or systemic adverse reactions, as well as concerns about long-term safety and potential side effects. Innate immunity activators rapidly respond to pathogen infections, particularly promising in antiviral and cancer treatments. Yet, these activators may exhibit a lack of specificity, sometimes leading to excessive inflammatory responses, and resistance issues may arise with long-term use. Consequently, despite the significant potential of adjuvants and innate immunity activators in the medical field, careful consideration is necessary in their development and application to ensure safety and efficacy.

While we have gained some understanding of immunologic adjuvants and innate immunity activators, the complex interactions between innate and adaptive immunity within the tumor microenvironment have not yet been fully deciphered. The challenge of fully elucidating the mechanisms of Coley’s toxins through multidisciplinary approaches remains significant.

## Proton therapy and carbon ion therapy

Proton therapy and carbon ion therapy are types of charged particle radiotherapy characterized by the Bragg peak, which offers advantages over conventional photon radiotherapy.^355^ This means that proton and carbon ion therapy deliver a lower radiation dose to surrounding tissues and reduce adverse effects.^356,357^ In 1990, the first hospital dedicated to proton therapy opened in Loma Linda,^358^ and the technology has since matured for cancer treatment. Due to higher costs and the technical demands of heavy ion accelerators, carbon ion therapy is less commonly applied in clinical practice, despite its potential advantages over proton therapy.^359^

### Head and neck cancer

Radiation therapy is an indispensable treatment modality for head and neck cancer. While proton therapy has been shown to have lower toxicity to surrounding tissues compared to photon therapy,^360^ its efficacy superiority remains controversial.^361^ A more recent retrospective study evaluated the survival benefits of proton therapy in patients with recurrent head and neck squamous cell carcinoma.^362^ Those who underwent salvage surgery followed by fractionated proton therapy experienced significantly improved local control rates and survival compared to patients who received salvage surgery with photon re-irradiation in previous studies. Two independent centers reported that carbon ion therapy demonstrated certain efficacy and safety in treating locally advanced head and neck cancer, particularly in cases of malignant melanoma and adenoid cystic carcinoma.^363,364^

### Non-small cell lung cancer (NSCLC)

Passive scattering proton therapy (PSPT) has shown advantages in safety, with a decreased rate of adverse events in unresectable NSCLC compared to historical data from patients receiving photon radiation therapy.^365^ A comparative study involving locally advanced NSCLC indicated that intensity-modulated proton therapy (IMPT) delivered lower radiation doses to surrounding organs, including the heart, lung, and esophagus, and resulted in a lower rate of adverse events compared to PSPT, although no significant difference in survival was observed.^366^ However, a recent study comparing the toxicity of intensity-modulated photon radiotherapy (IMRT) with PSPT concluded that there was no difference in radiation pneumonitis or local failure observed between the two groups.^367^ Therefore, further explorations and adjustments are necessary to determine the efficacy and safety of proton therapy.

Regarding carbon ion therapy, two dose escalation studies conducted by the National Institute of Radiological Sciences in Japan have investigated its efficacy and safety in patients with stage I NSCLC.^368,369^ These preliminary results suggest that single-fraction carbon ion therapy is effective and safe, even in elderly patients, although further studies for various stages of patients are needed.

### Prostate cancer

Due to the anatomic position of the prostate, radiation-related gastrointestinal and urinary injuries, erectile dysfunction, and hip fractures are distressing adverse events that cause secondary harm to patients. Previous research has shown that the benefits of proton therapy compared to photon radiation in prostate patients are controversial. A retrospective study compared toxicity and disease control rates in nonmetastatic prostate cancer and found that the proton therapy group had a higher rate of gastrointestinal morbidity than the IMRT group, although no difference in the rate of additional therapies between the two groups was observed.^370^ Another prospective study compared target coverage and doses to organs at risk between proton therapy and IMRT in patients with low-intermediate prostate cancer, also yielding a negative result.^371^ Data on carbon ion therapy for prostate cancer is limited, as only research groups from Japan and Germany have conducted large-scale cohort studies. According to German data, carbon ion therapy showed lower effectiveness and worse survival compared to proton therapy in patients with primary prostate cancer. The five-year overall and progression-free survival rates in the carbon ion group were 91% and 50%, respectively, compared to 98% and 85% in the proton therapy group.^372^ However, data from Japan demonstrated better efficacy of carbon ion therapy, with five-year overall survival rates of 100%, 99%, and 96% for low-risk, intermediate-risk, and high-risk patients, respectively, and biochemical recurrence-free survival rates of 92%, 89%, and 92%, respectively.^373^ Given the sample size, the results from the Japanese cohort (*n* = 2157) were more convincing than those from the German cohort (n = 92). However, further research is needed to verify the efficacy and safety of carbon ion therapy in different countries and among diverse populations.

### The advantages, disadvantages, and challenges of proton therapy and carbon-ion therapy

Radiotherapy, particularly proton therapy and carbon-ion therapy, shows potential advantages in the treatment of head and neck cancer and prostate cancer due to its ability to more precisely concentrate radiation doses on tumor tissue while reducing damage to surrounding healthy tissue. Proton therapy has demonstrated a lower rate of adverse effects in terms of safety, but its efficacy compared to conventional photon therapy remains a matter of debate. Carbon-ion therapy has shown varying results in terms of efficacy and survival rates across studies, indicating the need for further research to confirm its benefits. The widespread application of these two treatment technologies is limited by cost, technical requirements, and availability, but their potential to improve treatment outcomes and reduce side effects continues to drive the medical community to explore and refine these therapeutic methods.

## Photothermal and photodynamic therapy

The basic principle of photothermal therapy (PTT) and photodynamic therapy (PDT) involves using photosensitized materials in conjunction with light radiation to target diseases and achieve therapeutic purposes.^374^ Upon reaching the tumor lesion and being irradiated, photothermal and photodynamic agents convert light energy into heat or chemical energy to destroy tumor cells. PTT is practical in regulating temperature changes by adjusting the light intensity to control cell death,^375^ whereas the key mechanism of PDT is inducing reactive oxygen species (ROS),^376^ which is followed by the release of inflammatory mediators and the initiation of an immune response.^377,378^

### Current clinical application

Despite the potential therapeutic value of PTT, clinical studies are still limited. A pilot study explored the safety and efficacy of gold-silica nanoshells (GSNs) in low-intermediate-grade prostate cancer patients.^379^ GSNs exhibit maximal absorption in the near-infrared light spectrum and convert light energy into heat. In the 15 enrolled patients, after intravenous administration, GSNs accumulated in the prostate lesions due to the abnormal tumor vasculature, followed by subsequent near-infrared laser illumination. After 12 months of follow-up, prostate biopsies showed that 13 of 15 patients were cancer-free at the ablation zones.

Compared to PTT, PDT has been more extensively studied in clinical trials. A phase I study investigated the safety and efficacy of the photosensitizer Visudyne for treating vertebral metastases,^380^ finding significant pain reduction but no improvement in overall survival. Hypericin is a natural compound with phototoxicity to malignant cells.^381^ Based on this feature, hypericin is considered as a PDT agent. The FLASH study is a multicenter, placebo-controlled, double-blinded phase III trial that included patients with early-stage mycosis fungoides and cutaneous T-cell lymphoma (MF/CTCL) to examine the therapeutic effect of synthetic hypericin via PDT.^382^ The results indicated a higher index lesion response rate (ILRR) in the hypericin group compared to the placebo group, and after three cycles of administration, the ILRR increased to 49%.

### The advantages, disadvantages, and challenges of PTT and PDT

PTT and PDT are two optical treatment methods used for diseases such as cancer. PTT converts light energy into heat to destroy cancer cells in a targeted manner, characterized by minimally invasive nature, the capability for repeated applications, and the absence of surgical intervention. However, it requires precise temperature control to prevent thermal damage to surrounding healthy tissues. PDT relies on the selective interaction of photosensitizers with light to eliminate tumor cells, providing high selectivity and the possibility of triggering an immune response. Nevertheless, patients undergoing PDT are susceptible to photosensitivity post-treatment, and the procedure tends to be costly. Both techniques confront restrictions regarding the depth of light penetration, which can impact their efficacy against larger or more deeply located tumors. Despite these hurdles, PTT and PDT continue to hold significant positions in cancer therapy research due to their distinct therapeutic mechanisms and promising clinical utility.

## Anti-angiogenesis therapy

Inducing or accessing vasculature through angiogenesis is one of the hallmarks of cancer.^271^ Tumor angiogenesis exhibits unique characteristics, including a disordered structure, high permeability, and a lack of pericytes and smooth muscle cells, which differ significantly from angiogenesis under normal physiological conditions, such as embryonic development.^383^ The process of tumor angiogenesis is complex, regulated by both malignant and non-malignant cells through various pro-angiogenic and anti-angiogenic molecules and pathways in the tumor microenvironment,^384^ thus providing potential therapeutic targets.

### Molecular/pathway/cellular network in tumor angiogenesis

The vascular endothelial growth factor (VEGF) family is the group of molecules that has garnered the most research interest in tumor angiogenesis. The VEGF family, encoded by the human genome, consists of five members,^385^ among which VEGF-A (previously known as VEGF) is the most crucial mediator for activating endothelial cell reprogramming, proliferation, and migration by binding to VEGF receptor 2 (VEGFR2) during tumor angiogenesis.^386^ Upon binding, VEGFR2 becomes dimerized^387^ and activates downstream pathways, including RAS, ERK-MAPK, and PI3K-AKT signaling.^388–390^ In addition to its interactions with endothelial cells, VEGF-A can also influence immune cells through various mechanisms, including impeding dendritic cell maturation,^391^ increasing the infiltration of regulatory T cells,^392^ and inducing cytotoxic T cell exhaustion.^393^

The epidermal growth factor (EGF) family is another crucial component that contributes to tumor angiogenesis through both direct and indirect effects. For example, EGF promotes endothelial cell proliferation by binding to the EGF receptor (EGFR) and increases the secretion of VEGF by human pulmonary smooth muscle cells.^394^ In addition to the EGF family, the family of fibroblast growth factors (FGFs) and their receptors may play a significant role in tumor angiogenesis, as their overexpression has been observed across a wide range of malignancies with prognostic significance^395^; however, the underlying mechanisms are not yet fully understood.

### Translational value in clinical practice and future challenges

Targeting the VEGF-VEGFR signaling pathway is the primary strategy for anti-angiogenic tumor therapy. Bevacizumab was the first FDA-approved monoclonal antibody targeting VEGF-A for colorectal cancer (CRC) therapy and has shown significant improvements in overall and progression-free survival in patients with metastatic CRC.^396^ With advancements in basic research and clinical trials, bevacizumab is now used for a broader range of solid malignancies, including ovarian cancer,^397^ glioma,^398^ hepatocellular carcinoma (HCC),^22^ cervical cancer,^11^ and renal cell carcinoma.^399^

Another monoclonal antibody targeting VEGF-VEGFR, ramucirumab, exerts its effect by blocking VEGFR2. It was initially approved for the treatment of gastric and gastroesophageal junction adenocarcinoma, showing a survival benefit,^400^ and subsequently for NSCLC,^401^ CRC,^402^ and HCC.^403^

In addition to monoclonal antibody drugs, tyrosine kinase inhibitors (TKIs) have also attracted research interest due to the extensive involvement of tyrosine kinases in the downstream pathways of VEGFR, such as Sorafenib,^404–406^ sunitinib, lenvatinib, and apatinib.^407–409^ Also, there are several TKIs directly targeting VEGFR family. Nintedanib is a type of VEGFR-TKI that blocks certain signals inside cells, preventing the growth and spread of fibroblasts. In a phase III trial, it was used with docetaxel to treat advanced NSCLC and significantly improved PFS.^410^ Apatinib, another VEGFR-2 inhibitor, has shown a 76.7% disease control rate in treating NSCLC patients who did not respond to initial or follow-up treatments.^411^ Fruquintinib, a newer small molecule inhibitor, has also demonstrated promising results, with a 71% increase in progression-free survival compared to a control group in advanced non-squamous NSCLC treatment.^412^

### The advantages, disadvantages and challenges of anti-angiogenic drugs

Anti-angiogenic drugs effectively inhibit the formation of new blood vessels in tumors, thereby restricting the supply of nutrients and oxygen to cancer cells and slowing down tumor growth. These drugs have a strong targeting ability, with minimal impact on normal cells. When used in combination with other cancer treatments such as chemotherapy and targeted therapies, they can enhance therapeutic effects and delay the development of resistance. However, in some cases, they cause cardiovascular adverse reactions, such as hypertension and heart failure, which presents a limitation in the use of these drugs. Furthermore, although the combination treatment with other drugs is effective, the high cost could be a significant challenge for patients.

Primary and acquired drug resistance poses a significant challenge for anti-angiogenic therapy due to various mechanisms. Liver kinase B1 (LKB1), encoded by STK11, is a key factor in responding to stress conditions like hypoxia.^413^ A retrospective study found that the loss or deficiency of LKB1 in NSCLC leads to primary resistance to bevacizumab, possibly due to metabolic reprogramming in tumor cells.^414^ Tina et al. used a bevacizumab-resistant mouse xenograft model of human lung cancer and found that upregulated EGFR in stroma led to acquired resistance.^415^ To overcome drug resistance, basic studies are needed to explore the unrevealed compensatory adjustments and other changes in the tumor microenvironment after administration of anti-angiogenesis therapy, and to identify targetable molecules for combination therapy.

## Nanomedicine

With the approval and market introduction of the first nanomedicine (Doxil) by the FDA in 1995,^416^ the application of nanomedicines in cancer treatment began to gain attention. Nanomedicines are an emerging drug delivery system that leverages nanotechnology to enhance the bioavailability, stability, targeting, and therapeutic efficiency of drugs. These medicines typically refer to drug carriers or drug molecules at the nanoscale (1 to 100 nanometers), which can include traditional small-molecule drugs, peptides, proteins, nucleic acids, or other bioactive molecules.

The application of nanomedicines in cancer therapy encompasses a variety of nanocarriers and nanotechnologies. Currently, common nanomedicines used for tumors include lipid-based nanomedicines, inorganic nanomedicines, and polymer-based nanomedicines. Additionally, viral vectors and ADCs (Antibody-Drug Conjugates) can also be considered forms of nanomedicines, which have been discussed earlier.

### Lipid Nanomedicines

Liposomes are vesicles formed by the self-assembly of lipids into one or more concentric bilayers (unilamellar or multilamellar) with an enclosed aqueous compartment, ranging in size from 30 nm to the micrometer scale.^417^ Leveraging the enhanced permeability and retention (Enhanced Permeability and Retention, EPR) effect, liposome carriers offer the advantages of protecting encapsulated drugs from degradation, extending their half-life, and controlling drug release. By actively or passively delivering drugs to tumor tissues, liposomes contribute to reducing systemic side effects and enhancing cancer treatment. Currently, liposome nanoparticle formulations are among the most widely used nanomedicines. Encapsulating chemotherapy drugs is a common application of liposome nanomedicines, such as Doxorubicin and Paclitaxel. Doxil (liposome-encapsulated Doxorubicin), Myocet (liposome-encapsulated Doxorubicin HCl), and Abraxane (liposome-encapsulated Paclitaxel) have been FDA-approved for treating certain types of cancer, including breast cancer and non-small cell lung cancer. The LNPs mentioned earlier are also a type of lipid nanocarrier.

Solid Lipid Nanoparticles (SLNs) are drug carriers with a solid lipid core and surfactant coating, enhancing stability and drug release control. They can encapsulate both hydrophobic and hydrophilic drugs, offering advantages in cancer treatment. For instance, curcumin-loaded SLNs show potent cytotoxicity against SKBR3 breast cancer cells compared to curcumin alone,^418^ while RGD-modified doxorubicin SLNs enhance tumor targeting and therapeutic efficacy.^419^

Exosomal nanomedicines, often referred to as natural liposomes, are emerging as a rising star in drug delivery systems. Exosomes are small vesicles released by cells into the extracellular environment, playing a crucial role in cell-to-cell communication and capable of carrying proteins, RNA, DNA, and other biomolecules. Due to their natural characters and biological functions, exosomes have become a promising drug delivery platform. For instance, Rayamajhi et al. revealed that exosomes secreted by macrophages could be used for the delivery of Doxorubicin (a clinical drug) for breast cancer treatment.^420^ In the future, liposome nanomedicines are expected to see more clinical translation.

### Inorganic nanomedicines

Inorganic nanomedicines, which include metal nanoparticles (such as gold, silver, and iron oxides), quantum dots, carbon-based materials (like carbon nanotubes and graphene), and other particles (mesoporous silica, magnetic nanoparticles, and nanoscale calcium materials), are utilized for cancer diagnosis and treatment. As mentioned earlier, gold nanoparticles serve as a medium for photothermal therapy, killing tumor cells through localized heating. Iron oxide nanoparticles (such as Fe3O4 and γ-Fe2O3) can be used as contrast agents for magnetic resonance imaging (MRI) to enhance tumor visualization.^421^ Another kind of nanomedicine uses Titanium dioxide ^1^(TiO2) and zinc oxide (ZnO) to generate reactive oxygen species (ROS),^422^ which can kill tumor cells under light exposure. Additionally, certain nanomedicines, such as those containing Fe3 + , SiO2, Pt, and Li, can induce various cell death pathways, including ferroptosis, pyroptosis, autophagy, and necroptosis.^423^ Certain metal-organic frameworks (MOFs) are capable of encapsulating and releasing therapeutic gases, such as nitric oxide (NO), which has antitumor and anti-angiogenic effects. Silica nanoparticles have been shown to activate the immune system and enhance antitumor immune responses. They can activate immune cells through pattern recognition receptors (such as Toll-like receptors, TLRs), thereby promoting the clearance of tumor cells. Recent reports also suggest that inorganic nanomaterials can induce macrophage polarization. Researchers have developed a hollow mesoporous Prussian blue (HMPB) nanosystem disguised by a macrophage membrane.^424^ The released iron ions promoted the differentiation of M1-like tumor-associated macrophages (TAMs) through the interferon regulatory factor 5 (IRF5) pathway, stimulated cytotoxic T cells, and generated an effective antitumor effect.^425^ The applications and therapeutic strategies involving inorganic nanomedicines are highly innovative and cover a broad range of approaches.

### Polymeric Nanomedicines Polymeric nanoparticles

Polymeric Nanomedicines Polymeric nanoparticles, such as poly(lactic-co-glycolic acid) (PLGA) nanoparticles, are widely used in cancer treatment. These nanoparticles can encapsulate drugs, control their release, and enhance drug targeting. PLGA-packed drugs include both chemotherapy agents and immunosuppressants. Dasharath Chaudhari developed paclitaxel (PTX)-loaded adenosine (ADN)-conjugated PLGA nanoparticles to combat triple-negative breast cancer (TNBC), which were found to be biocompatible and exhibited improved anti-TNBC activity. Another experiment demonstrated that in a subcutaneous model of MC38 colon cancer,^426^ PLGA nanoparticle therapy was as effective as treatment with soluble anti-PD-L1 monoclonal antibody (mAb), resulting in significantly reduced tumor growth.^427^ In other studies, Chi-Son Chang and colleagues reported that fenbendazole-incorporated PLGA nanoparticles exerted significant anticancer effects in epithelial ovarian cancer cells and xenograft models, including patient-derived xenografts (PDX).^428^ PLGA has also been involved in preclinical studies for combination therapy. One study justifies additional preclinical safety trials and the clinical evaluation of 177Lu-PLGA(RGF)-CXCR4L as a potential combined treatment for colorectal cancer.^429^ Guen Tae Kim showed that the antitumor effect of the chemotherapy AC regimen (Adriamycin and cyclophosphamide) was increased by cotreatment with 1-palmitoyl-2-linoleoyl-3-acetyl-rac-glycerol (PLAG) in the MDA-MB-231 TNBC xenograft mouse model.^430^ Among other polymers, dendrimers, as a special class of polymers, participate in drug transport. G5 PAMAM dendrimers have demonstrated successful folate-receptor-targeted delivery of doxorubicin.^431^ Another study reported that dendrimer-functionalized nanoparticles delivering the p53 tumor suppressor gene to the tumor site resulted in an improved antiproliferative effect compared to the free naked gene.^432^

### Advantages, disadvantages, and challenges

Nanomedicines offer a multitude of benefits over conventional free-form drugs, providing a more effective approach to drug delivery. By enhancing drug solubility, they safeguard medications from early degradation, which in turn boosts stability and bioavailability. They also minimize off-target interactions within the body, extending the drug’s circulation time and improving therapeutic outcomes. Additionally, nanomedicines increase the concentration of drugs at the site of pathology while simultaneously reducing their presence in healthy tissues, thereby mitigating toxic side effects. The controlled release of drugs and their targeted distribution to specific tissues further optimize pharmacokinetic profiles. Moreover, these advanced systems facilitate the penetration of formidable biological barriers, such as the blood-brain barrier, surmounting obstacles that hinder the delivery of therapeutics.

Although inorganic nanomaterials show great potential in various fields, they also have significant drawbacks. Firstly, these materials may have potential biological toxicity. Due to their high surface area, they may interact unpredictably with biomolecules, leading to cell or tissue damage. Secondly, stability issues in biological systems, such as easy aggregation and sedimentation, or coverage by biomolecules, may reduce their therapeutic effectiveness. Additionally, the distribution, metabolism, and clearance mechanisms of these nanomaterials in the body are not fully understood, affecting the assessment of their safety and efficacy. Lastly, the long-term health and environmental risks of inorganic nanomaterials remain unclear, increasing concerns about their widespread application. Therefore, despite the innovative applications that inorganic nanomaterials may bring, their potential risks must be thoroughly researched and strictly managed before they are used clinically and environmentally.

Currently, the translation of nanomedicines has not progressed as rapidly as the numerous positive preclinical results would have suggested. Key issues related to the clinical development of nanoparticle nanomedicines include biological challenges, biocompatibility and safety, large-scale manufacturing, government regulations, intellectual property (IP), and overall cost-effectiveness compared to current therapies. The cost, in particular, has limited the clinical transfer of this approach.

## The combination therapy against tumor

Combination therapy in the field of oncology has achieved significant advancements, becoming a key strategy for enhancing treatment efficacy, combating drug resistance, and extending patient survival. Notably, the combination of immune checkpoint inhibitors has demonstrated synergistic effects across various types of cancer, improving therapeutic outcomes. The latest data from the IMbrave150 study show that patients with Hepatocellular Carcinoma (HCC) who received the recommended front-line combination therapy of Atezolizumab and Bevacizumab (T + A) had a mOS of 19.2 months compared to 13.4 months with sorafenib. In the Chinese subgroup of patients, the mOS was extended to two years compared to 11.4 months with sorafenib. Moreover, the ORR for this treatment reached 30%.^400^ Additionally, recent findings suggest that “T+A” regimen paired with TACE (Transarterial Chemoembolization) or HAIC (Hepatic Artery Infusion Chemotherapy) may be a significant research direction for large hepatocellular carcinoma (HCC). Hence, the scientifically sound combination of treatments in clinical practice will benefit more cancer patients.

The year 2023 was a fruitful year for research in combination therapies. In March, Novartis announced that the FDA approved the combination therapy of Tafinlar (dabrafenib) and Mekinist (trametinib), making Tafinlar + Mekinist the first approved targeted combination therapy for the treatment of pediatric patients with low-grade gliomas carrying the BRAF V600E mutation (Supplementary Table 3).^433^ In May, Professor Ruihua Xu’s team’s SPOTLIGHT study on advanced gastric cancer showed that the combination of zolbetuximab and mFOLFOX6 reduced the risk of disease progression or death by 24.9% (*P* = 0.0066) compared to the placebo + mFOLFOX6 group, with median progression-free survival (mPFS) of 10.61 months and 8.67 months, respectively.^434^ In June of the same year, the clinical trial of talazoparib and enzalutamide for metastatic castration-resistant prostate cancer (mCRPC) in adults was approved for the first time.^435^ This combination therapy can reduce the progression and mortality rates by 55% in mCRPC patients with HRR mutations. In November, the JUPITER-02 study, the world’s first clinical study also led by Professor Ruihua Xu’s team comparing first-line immunotherapy combined with chemotherapy to chemotherapy alone in nasopharyngeal carcinoma (NPC), confirmed that the treatment with tislelizumab combined with chemotherapy significantly improved overall survival (OS) for the first-line treatment of recurrent or metastatic NPC, with a 3-year OS rate of 64.5%, and a 37% reduction in the risk of death.^436^ This study set a record for OS benefits in patients with advanced NPC. Additionally, a clinical study reported in the New England Journal of Medicine revealed that first-line treatment with osimertinib-chemotherapy led to significantly longer PFS than osimertinib monotherapy among patients with EGFR-mutated advanced non-small cell lung cancer (NSCLC).^437^ These studies mark significant advancements in combination therapies for cancer treatment.

### Combined therapy with immune checkpoint inhibitors

From 2006 to March 20, 2024, the US Food and Drug Administration has approved 90 combination therapies for cancer (Supplementary Table 3). The use of immune checkpoint inhibitors (ICIs) in conjunction with other treatment modalities is noteworthy. The combination of Nivolumab and Ipilimumab has appeared 8 times, covering various cancers including RCC, melanoma, mesothelioma, and NSCLC et al. Combinations with chemotherapy have emerged 16 times, including Pembrolizumab + Chemotherapy, Atezolizumab + Nab-Paclitaxel, Atezolizumab + Carboplatin + Etoposide, and Nivolumab + Chemotherapy. These combination regimens have become primary first-line options for a range of cancers in clinical practice.

In the latest guidelines from the American Society of Clinical Oncology (ASCO) and the National Comprehensive Cancer Network (NCCN), the integration of immunotherapy with other treatment approaches has become an essential component of first-line therapy for various cancers. Considering the findings from the 2021 KEYNOTE-826 study, the administration of Pembrolizumab combined with TP (paclitaxel and cisplatin or paclitaxel and carboplatin) along with optional Bevacizumab has markedly enhanced the OS and PFS for patients battling advanced cervical cancer.^438^ Following these outcomes, the FDA formally granted approval for this combined treatment approach as the new standard first-line therapy for advanced cervical cancer. The 2023 ASCO meeting presented the 39.1-month follow-up data from the KEYNOTE-826 trial, which indicated that incorporating Pembrolizumab into chemotherapy with or without Bevacizumab significantly lowered the mortality risk by 40% in individuals with a PD-L1 CPS (Combined Positive Score) of 1 or greater, by 37% across the entire studied population, and by 42% in those with a CPS of 10 or higher, while maintaining an acceptable safety profile. For non-small cell lung cancer (NSCLC), Pembrolizumab is recommended for patients with high PD-L1 expression, while for those with low or unknown PD-L1 expression levels, combinations of Nivolumab with Ipilimumab and chemotherapy, or Pemetrexed with platinum-based chemotherapy are recommended. The treatment recommendation for advanced melanoma includes the combination of Nivolumab and Ipilimumab. Based on the clinical trials results from CheckMate 214,^439^ KEYNOTE-426,^440^ JAVELIN 101,^441^ CheckMate 9ER,^442^ first-line treatment recommendations for renal cell carcinoma (RCC) include Nivolumab+Ipilimumab, Pembrolizumab+Axitinib, Avelumabas + +Axitinib, well as the combination of Nivolumab+Cabozantinib. based on the results from the KEYNOTE-B61^443^ study, the combination of Lenvatinib and Pembrolizumab or Everolimus has shown durable anti-tumor activity in patients with previously untreated non-clear cell RCC (including papillary and chromophobe renal cell carcinoma). The objective response rate (ORR) reached 49%, with 74.6% of patients experiencing responses lasting at least 12 months. The 12-month PFS rate was 63%, and the 12-month overall survival (OS) rate was 82%, indicating promising survival outcomes. These results further support the use of Pembrolizumab and Lenvatinib as a first-line treatment strategy for patients with advanced non-clear cell RCC. For patients with head and neck squamous cell carcinoma (HNSCC), Pembrolizumab alone or in combination with chemotherapy is an option. The first-line treatment recommendation for HCC is the combination of Atezolizumab with Bevacizumab. Pembrolizumab is the first-line treatment choice for colorectal cancer (CRC) with high microsatellite instability (MSI-H) or mismatch repair deficiency (dMMR). For triple-negative breast cancer (TNBC), the first-line treatment recommendation includes the combination of Ipilimumab with chemotherapy.

Combined drug therapy in clinical trials and preclinical basic research for cancer treatment most frequently involves co-administration with immunosuppressants. Inhibitors targeting PD-1 (such as Nivolumab and Pembrolizumab) and inhibitors targeting CTLA-4 (such as Ipilimumab) are star drugs in immunotherapy, and they have been widely studied in the treatment of various solid tumors and hematological cancers. In a phase II/III clinical trial (RELATIVITY-047) examining combination therapy for unresectable or untreated metastatic melanoma, satisfactory improvements are seen with Relatlimab (anti-LAG-3) and Nivolumab (anti-PD-1). With this combination, the 1-year PFS rate increased from 36.0% to 47.7% and the median PFS improved dramatically from 4.6 months to 10.1 months.^444^ However, further follow-up is required to assess the ultimate therapeutic efficacy.^444^ In preclinical research, combination therapy involving various treatment modalities has been explored. For instance, Guangji Zhang et al. conducted a study combining Nivolumab and GD2 CAR-T treatment in Glioblastoma. This combination exhibited the highest efficacy, leading to an extension of survival in mice with tumor burden for up to 60 days.^445^ Another study by Lanqi Gong et al. demonstrated that CD70 blockade, when combined with anti-PD-1 treatment, synergistically reinvigorated T-cell immunity against nasopharyngeal carcinoma. The evaluation of anti-CD70 monotherapy in combination with anti-PD-1 therapy using xenograft-derived organoids and humanized mice showed improved tumor-killing efficacy.^446^ Additionally, a recent study reported that PD-1 + IL-2 combination therapy significantly alters the differentiation program of PD-1 + TCF-1+ stem-like CD8 + T cells, which merits consideration as a potential regimen for cancer treatment.^447^ Also, clinical trials of combination ICIs have achieved significant results, and more clinical trials are underway. We have listed some representative combination cancer treatment clinical trials in Table 6. The clinical trials with results showed that patients received more benefits from the combination treatment. Meanwhile, new types of cancer therapy such as oncolytic virus have already started their clinical trials with ICIs.

**Table 6:** Summarized clinical trials of ICIs combinations with other treatments in cancers

Combination	NCT Number	Phase	Conditions	Regime	mOS (m)	mPFS (m)	ORR (%)
PD-1+cell therapy	NCT02843204	I/II	Malignant Solid Tumour	Pembrolizumab+allogeneic NK cells vs. pembrolizumab	15.5 vs. 13.3	6.5 vs. 4.3	36.4 vs. 18.5
PD-1+chemotherapy	NCT03189719	III	advanced esophageal cancer and Siewert type 1 gastro-esophageal junction cancer	pembrolizumab+chemotherapy(5-fluorouracil and cisplatin) vs. PLACEBO+chemotherapy	12.4 vs. 9.8	6.3 vs. 5.8	45 vs. 29.3
PD-1+small molecular inhibitor	NCT02133742	IB	Renal Cell Carcinoma	Axitinib 5 mg + Pembrolizumab (MK-3475) 2 mg	NA	20.9[1]	NA
CTLA-4+monoclonal antibody	NCT03241173	I/II	Advanced malignancies	INCAGN01949 350 mg + Ipilimumab 1 mg/kg	NA	4.6	0
PD-1+small molecular inhibitor*2	NCT02130466	I/II	Melanoma/Solid Tumors	Pembrolizumab+Dabrafenib+Trametinib vs. Placebo+Dabrafenib+Trametinib	46.3[2] vs. 26.3	17.0 vs. 9.9	NA
PD-1 + mRNA vaccine	NCT02529072	I	Malignant Glioma/Astrocytoma/Glioblastoma	nivolumab vs. nivolumab+DC vaccine	8 vs. 15.3[2]	4.3 vs. 6.3	NA
PD-1+Oncolytic Virus + CTLA-4	NCT03206073	I/II	Colorectal Cancer et al.	1/Arm A1 Pexa-Vec 3 x 10E^8 Plaque-forming Unit (Pfu) + Durvalumab 1500 mg vs. 2/Arm A2 Pexa-Vec 1 x 10E^9 Pfu +Durvalumab 1500 mg vs. 3/Arm B1 Pexa-Vec 3 x 10E^8 Pfu + Durvalumab 1500 mg +Tremelimumab 300 mg vs. 4/Arm B2 Pexa-Vec 1 x 10E^9 Pfu + Durvalumab 1500 mg +Tremelimumab 300 mg	3 vs. 2.3 vs. 2.25 vs. 1.65	0 vs. 8.3 vs. 0 vs. 7.1	NA
PD-1 +	NCT03765190	I/II	Neoplasm Metastasis	proton radiation therapy+PD-1 antibody	NA	NA	NA
PD-1+Photodynamic Therapy	NCT05386056	II	Esophageal Squamous Cell Carcinoma	Pembrolizumab+Photodynamic Therapy	NA	NA	NA

[1] Upper limit of 95% CI was not reached since a large proportion of participants in the analysis set had their PFS data censored and there were insufficient number of participants with events to calculate the full 95% CI

[2] The upper limit is not estimable due an insufficient number of patients and events. *TKI* Tyrosine kinase inhibitors. Data obtained fromclinicaltrial.gov (Accessed on 24th March 2024)

### Combined strategies in small molecular inhibitors

In the FDA-approved combination therapy, the combination of small molecular drugs appeared 22 times, including Neratinib + Capecitabine, Encorafenib + Cetuximab, Tucatinib + Trastuzumab + Capecitabine, Ibrutinib + Rituximab, and Olaparib + Bevacizumab. This indicates that the combined use of small molecule inhibitors is also commonly employed in clinical practice. In the latest clinical treatment guidelines, the combined use of targeted therapy drugs is tailored to specific cancer types and molecular markers, encompassing a variety of different combinations. For instance, in patients with non-small cell lung cancer (NSCLC) harboring EGFR mutations, EGFR inhibitors such as Erlotinib may be combined with anti-angiogenic drugs like Bevacizumab.^448^ For NSCLC with ALK rearrangements, ALK inhibitors like Crizotinib may be used in conjunction with other treatment modalities.^449^ In the treatment of HER2-positive breast cancer, HER2 inhibitors such as Trastuzumab may be combined with Pertuzumab and chemotherapy.^450^ The combination of the BRAF inhibitor Dabrafenib with the MEK inhibitor Trametinib has become the standard treatment for melanoma with BRAF V600E/K mutations. In ovarian and breast cancers with BRCA mutations, PARP inhibitors like Olaparib may be combined with chemotherapy.^451^ CDK4/6 inhibitors such as Palbociclib are used in combination with endocrine therapy for hormone receptor-positive breast cancer.^452^ Additionally, IDH1/IDH2 inhibitors like Ivosidenib are used in combination with Azacitidine for leukemias and other solid tumors carrying the respective mutations.^453^

Due to the wide variety of small molecule inhibitors, its combination with other drugs is very common. While in preclinical research, blocking the oncogene or its signaling pathway by using small molecule inhibitors combined with other treatments is a regular method to test therapeutic efficiency. For example, Liu et al. successfully combined a PI3K inhibitor, the downstream of TROY, with Sorafenib to suppress tumor development in a HCC mouse model.^454^ Jiang et al. demonstrated that the combination of ARV-771 and romidepsin keep tumor grew more slowly than HDAC or BET agent alone in ESCC xenograft.^455^ The combination of small molecular inhibitor and CRISPR system as a drug screening system to find neoantigen is also another application in basic research. Zheng et al. integrated the screening of a small-molecule inhibitor library and a druggable CRISPR library, revealing that GSK-J4 exhibits synthetic lethality with donafenib in liver cancer.^456^

Small molecule inhibitors are frequently incorporated into clinical trials for combination therapies, owing to their lower toxicity profile in humans. One clinical trial showed that the combination of lapatinib plus trastuzumab improved disease-free survival from 1.0 year (lapatinib alone) to 1.9 years, and overall survival from 3.6 to 3.9 years.^457^ A clinical trial from the New England Journal of Medicine, showed that in a cohort of 44 patients with rare Colorectal Cancer carrying the Mutated KRAS G12C, the combination therapy of Adagrasib and Cetuximab decreased the percentage of grade 3 or 4 adverse reactions compared to Adagrasib monotherapy and increased the drug response rate from 19% to 46%.^458^ These findings indicate the strong efficacy of the combination.^459^ These results indicate that the addition of small molecule inhibitors to other treatments can extend patients’ lives.

### Combined use of anti-angiogenic drugs

The combination of anti-angiogenic drugs with other treatment regimens is a common clinical practice for medication use. In the FDA approval combination treatment table, the combination of anti-angiogenic therapies is mentioned 14 times, which include: Bevacizumab + Atezolizumab, Ramucirumab + Erlotinib, Axitinib + Pembrolizumab, Lenvatinib + Pembrolizumab, Neratinib + Capecitabine, Tucatinib + Trastuzumab + Capecitabine, and Enfortumab Vedotin-ejfv + Pembrolizumab et al. This suggests the importance of anti-angiogenic drugs as adjuvant therapy for combined anti-tumor therapy.

In the latest clinical guidelines, the combination of anti-angiogenic drugs with other treatment modalities has become part of the first-line treatment regimens for specific cancer patient subgroups. For instance, in non-small cell lung cancer (NSCLC), the combination of Bevacizumab with chemotherapy is recommended for patients without EGFR or ALK gene mutations.^460^ In patients with metastatic colorectal cancer (CRC), Bevacizumab is also used in conjunction with chemotherapy, especially for those with tumors of KRAS, NRAS, and BRAF wild-type status.^461^ Additionally, for patients with advanced hepatocellular carcinoma (HCC), the combination therapy of Bevacizumab with the immune checkpoint inhibitor Atezolizumab is a first-line option. In the treatment of breast and ovarian cancer, anti-angiogenic drugs may also be combined with chemotherapy or other targeted therapy drugs based on the patient’s hormone receptor status and genetic characteristics. The selection of these combination treatment regimens is based on the analysis of patient biomarkers, genetic testing results, and clinical features to ensure that patients receive the most effective treatment.

The novel mechanism of anti-angiogenesis not only use of anti-VEGF-related products, particularly when combined with other drugs, deserves attention. Kayoko Hosaka et al. discovered how KRAS-mutated epithelial cancers resist anti-angiogenic drugs by using ANG2, overcoming anti-VEGF actions. Combining anti-VEGF and anti-ANG2 treatments showed strong, synergistic effects against these cancers.^462^ Another recent study reported that MFGE8 activates the ERK/AKT pathway to promote angiogenesis in esophageal squamous cell carcinoma (ESCC). Blocking the angiogenesis function of MFGE8 with its neutralizing antibody, combined with cisplatin treatment, could minimize the tumor size in ESCC tumor-burden mice [L]. These preclinical studies provide potential treatment ideas for cancer patients.

Bevacizumab, as a hot drug against angiogenesis, has also been involved in various clinical trials. One clinical trials combined Bevacizumab and Erlotinib in phase II advanced ovarian cancer showed the mPFS reached 25.6 months.^463^ A recent clinical trial published in the New England Journal of Medicine, based on data from 246 patients with Refractory Metastatic Colorectal Cancer, revealed that the median overall survival in the combination therapy group (Trifluridine-Tipiracil plus Bevacizumab) was 10.8 months, which is greater than the 7.5 months in the Trifluridine-Tipiracil monotherapy group, providing a new direction for the treatment of Refractory Metastatic Colorectal Cancer.^464^ Another new drug targeting both VEGFR and EGFR, Rivoceranib, has demonstrated remarkable efficacy in tumor treatment. Clinical trials have indicated that the combination of camrelizumab and rivoceranib significantly improves overall survival and progression-free survival, and provides clinically meaningful benefits for patients with HCC compared to sorafenib alone in a cohort of 543 patients.^414^ Given the promising clinical activity observed, further exploration of the combination with anti-angiogenic drugs is warranted as a potential alternative to available treatments.

### The advantages, disadvantages, and challenges of combination treatment

Even though combination therapies have received multiple FDA approvals and have advantages in overcoming complex diseases and give patients customizable treatment to against tumor. There are still some shortcomings and challenges. First, cancer patients who meet the criteria for combination therapy may need to be further selected to reap benefits while avoiding potential pitfalls. From the combination therapies approved by the FDA and the guidelines, the entry criteria for combination therapy are relatively strict. For instance, the talazoparib + enzalutamide combination therapy approved in November 2023 is specified for the treatment of patients with HRR gene-mutated metastatic castration-resistant prostate cancer. In the pembrolizumab + chemotherapy combination therapy approved on November 25, 2020; it is explicitly stated that TNBC patients must have PD-L1 expression above a certain threshold (CPS ≥ 10) (Supplementary Table 3). Additionally, the combined use of different drugs may not always be compatible, and drug interactions may be affected, with the potential for increased the frequency of adverse events on the patient due to the use of multiple medications. One clinical trial (NCT02660034) reported the occurrence of nausea (63%), fatigue (53%), diarrhea (35%), vomiting (31%), and asymptomatic grade 3–4 hepatic immune-related adverse events (39%) in the cohorts receiving combination treatment with Pamiparib and Tiselizumab.^465^ Therefore, the selection of drugs and the determination of drug dosages are crucial. Further animal studies and clinical trials are necessary. Moreover, there is an urgent need for predictive biomarkers to guide the entire treatment process. Since the situation with combination therapies differs from monotherapy, in addition to regular imaging tests, some blood test indicators also need to be clearly redefined to accurately describe the patient’s treatment response. For patients, combination therapies inevitably increase drug costs. In the future, how to reduce the cost of combination therapies will also be an important issue for drug combination strategies.

## Conclusions and prospectives

The aim of oncology drug research and development is to provide more effective and targeted treatment methods for suppressing tumor growth and metastasis. In this review, we summarize the progress and application of various oncology drugs, which encompass a range of types and mechanisms. The advantages, disadvantages, challenges, and representative drugs or clinical trials related to cancer treatments discussed herein are compiled in the table below, offering a comprehensive overview and comparison of different strategies (Table 7).

**Table 7:** Comprehensive overview of cancer therapy strategies: definitions, advantages, challenges, and comparative analysis with application examples

Treatment options	Description	Advantages	Disadvantages	Challenges	Cancer Applications Examples
Small molecular inhibitor	Low molecular weight compounds that target specific cellular pathways or enzymes.	Simpler and less expensive synthesis and preparation processes	Limited inhibitory effects on membrane proteins and secretory proteins	Challenges: Undruggable proteins Solution: Covalent modulation, Allosteric inhibition, PROTACs, MGDs	Sotorasib: the first small molecular inhibitor targeting specific KRAS gene mutations.
Peptide drug	Short chains of amino acids that mimic natural peptides or proteins, with therapeutic effects.	High efficiency at low concentrations Strong specificity Good safety profile Easy synthesis Low cost	Poor stability Difficult oral administration Poor membrane permeability	Enhancing stability Facilitating oral delivery Improving membrane permeability	Lutathera: for Gastrointestinal pancreatic neuroendocrine tumor
Monoclonal Antibody Therapy	Monoclonal antibodies (mAbs) are produced by B cells and specifically target antigens.	High specificity for cancer cells Reduced toxicity to healthy cells Fast response and long-term immune activity	The same with the challenges.	High production costs Antibody-related side effects Low penetration efficiency Long half-life with risks of adverse effects	Nivolumab: Anti PD-1 agents
Antibody drug conjugates (ADCs)	Combination of a monoclonal antibody and a cytotoxic drug, targeting with precision.	Specific antigen-antibody targeting reduces systemic toxicity. Greater design flexibility compared to conventional antibodies. Customizable for various cancers through antigen and antibody selection. Precise control of Drug-to-Antibody Ratio (DAR) for optimized efficacy and safety.	The same with the challenges.	Low internalization and efficiency. Target-off toxicity. Balancing therapeutic activity with payload toxicity. Complex metabolism and lack of uniformity in metabolic properties. Toxicities, including on-target/off-tumor and off-target/off-tumor effects. Unclear mechanisms of ADC resistance. Production and quality control difficulties.	Akalux: for head and neck cancer
Cell therapy(CAR-T as an example)	A type of immunotherapy where a patient’s T cells are genetically modified to recognize and kill cancer cells.	Targeted therapy Durability of response Enhanced potency Adaptive immune response Evolution of CAR-generations	Complex manufacturing process Toxicity and safety concerns Limited proliferation in early generations Need for clinical validation solid tumor challenges Allogeneic concerns	Challenge: Antigenic drift; System cytokine toxicities; Lack of effective targets for solid tumors; Tumor microenviroment suppression; Tumor barrier; GVHD Solution:Tandem CAR-T; Inhibit IL-6; New tumor-specific antigens finding; co-treatment; utilize delivery routes; CD52 knockout	CD19 + CAR-T
Gene therapy(CRISPR as an example)	CRISPR/Cas9 is a revolutionary gene-editing technology that allows researchers to make precise and efficient edits in the genome of cells.	Precise genome editing. Efficient and versatile tool. Potential for treating genetic disorders and cancer. Enhances T cell therapies. Aids in drug development.	Off-target effects. Immune reactions. Long-term efficacy. Delivery system selection	simplicity of design studies of longer safety in body	NCT03545815: evaluates the feasibility and safety of CRISPR-Cas9 mediated PD-1 and TCR gene-knocked out chimeric antigen receptor (CAR) T cells in phase 1 patients with mesothelin-positive multiple solid tumors
Neoantigen and cancer vaccine	Vaccines designed to stimulate the immune system to recognize and attack cancer-specific neoantigens.	Effective at preventing tumor occurrence. Potential to stimulate long-term immune memory for lasting protection against cancer recurrence.	Efficacy is inconsistent due to individual antigen variability and immune microenvironment interference.	Enhancing vaccine immunogenicity, as some patients show no response to vaccines. Improving synthetic peptide design to increase MHC binding efficiency.	VGX-3100: a double plasmid vaccine encoding HPV protein E6 and E7.
Oncolytic virus	Viruses that are genetically modified to infect, kill, and break down cancer cells directly.	Target cancer cells selectively and activate immunity. Tumor susceptibility due to genetic defects. Easy genome modification for targeting. Synergy with ICIs.	limitation of targeting specificity issue of immune response	Improve targeting specificity. Manage immune reactions. Optimize dosage. Address adverse events.	T-VEC (Talimogene Laherparepvec): for melanoma, breast cancer, sarcoma, head and neck, colorectal cancer glioma
Immunologic adjuvant and innate immunity activator	Substances that enhance the body’s immune response, particularly activating the innate immune system.	Enhance vaccine effectiveness. Reduce antigen quantities needed. Prompt immune response to infections. Versatile for various populations and diseases.	Potential safety and side effects. Non-specific immune activation leading to inflammation.	Risk of resistance with prolonged use. Unclear mechanisms in tumor environments. Difficulties in fully understanding adjuvant mechanisms.	Toll-like receptors (TLRs) agonists
Proton therapy and carbon ion therapy	High-precision radiotherapy techniques using protons or carbon ions to target tumors with minimal damage to surrounding tissue.	Precise radiation concentration on tumors. Reduced damage to surrounding healthy tissue. Lower adverse effects for proton therapy.	High costs and technical requirements. Limited availability.	Debate over efficacy compared to photon therapy. Varied efficacy and survival results for carbon-ion therapy.	
Photothermal and photodynamic therapy	Light-based therapies where heat (photothermal) or reactive oxygen species (photodynamic) are used to destroy cancer cells.	PTT: Minimally invasive, repeatable, no surgery needed. PDT: High selectivity, potential immune response triggered.	Both: Limited by light penetration depth affecting large or deep tumors.	PTT: Requires strict temperature control to avoid damage. PDT: Post-treatment photosensitivity, high costs.	GSNs
Anti-angiogenesis therapy	Treatments that inhibit the formation of new blood vessels in tumors, cutting off their nutrient supply.	Inhibit tumor growth by restricting nutrient and oxygen supply. Strong targeting with minimal impact on normal cells. Enhance effects of other cancer treatments and delay resistance.	High costs with combination therapies.	Cardiovascular adverse reactions (e.g., hypertension, heart failure). Primary and acquired drug resistance due to various mechanisms.	Nintedanib: a type of VEGFR-TKI
Nanomedicines	Treatments that inhibit the formation of new blood vessels in tumors, cutting off their nutrient supply.	Enhanced solubility and bioavailability Minimized off-target effects Targeted drug delivery Extended circulation time Controlled release at disease site Penetration of biological barriers	Potential toxicity Stability and aggregation issues Unclear body mechanisms Unknown long-term risks	Biological complexity Safety and biocompatibility concerns Manufacturing scalability Regulatory compliance Intellectual property management Cost-effectiveness compared to existing therapies	Iron oxide nanoparticles (such as Fe3O4 and γ-Fe2O3)
Combination therapy	The use of two or more therapeutic approaches simultaneously to treat complex diseases more effectively.	Personalized treatment strategies Potential for increased efficacy against complex diseases FDA-approved for various cancer types Targeted treatment for specific genetic mutations (e.g., HRR gene-mutated prostate cancer)	Strict patient selection criteria Incompatibility and drug interactions with multiple medications Increased frequency of adverse events (e.g., nausea, fatigue, diarrhea, vomiting, hepatic immune-related adverse events)	Critical need for precise drug selection and dosage determination Requirement for additional animal studies and clinical trials Urgent need for predictive biomarkers to guide treatment Redefinition of blood test indicators for accurate treatment response assessment Increased drug costs for patients	Atezolizumab with Bevacizumab

As more foundational research delves into the functions and mechanisms of tumors, the future of cancer treatment looks promising. In addition to the ongoing development and expansion of treatments such as ICIs, gene therapy, CAR-T, cancer vaccines, and oncolytic viruses (OVs), and the shift from traditional frontline care to combination therapies, there are several noteworthy aspects to consider for the future

### Firstly, AI technology will penetrate every aspect of cancer therapy

Current research is primarily focused on integrating AI with healthcare to facilitate automated diagnosis in tissue pathology. In June 2023, a study published in JAMA, assessed the diagnostic performance of GPT-4 with complex medical records. Findings indicated that GPT-4 identified the correct diagnosis as the primary diagnosis in nearly 40% of instances and offered accurate potential diagnoses in two-thirds of the cases.^466^ Microsoft’s recently introduced biomedical model, LLaVA-Med, has the ability to deduce patients’ pathological conditions from imaging such as CT and X-ray. In the realm of cervical cancer screening, Huawei, partnering with KingMed Diagnostic, has developed an AI-assisted model that achieves a high specificity rate exceeding 60%, with an interpretation accuracy for negative slides surpassing 99% and a detection rate for positive abnormalities above 99.9%. These advancements mark merely the beginning. The application of AI in the realm of small molecule inhibitors has been discussed, highlighting its role in enhancing drug design, refining manufacturing processes, and forecasting and assessing outcomes. The convergence of oncology big data with deep learning AI offers a distinctive platform, and the field anticipates innovative findings over the coming decade.

The revelations of the biological principles behind cancer heterogeneity based on multi-omics research are guiding new directions in tumor treatment. Spatial omics, TOF mass spectrometry, and CODEX (CO-Detection by IndEXing) are among the omics studies currently underway in preclinical research. These multi-omics technologies combine molecular characterization with spatial resolution, enabling the dissection of tumor molecular structures and the spatial organization of tumor microenvironments. slide-DNA-seq has been applied to tumor samples, allowing for the spatial resolution of genomic data within intact tissue samples. This method has the potential to identify, locate, and characterize individual clones within the three-dimensional tumor structure, thus holding significant promise for elucidating intra-tumor heterogeneity and the evolution of tumor clones during treatment. Spatial proteomics data is also being used to guide clinical trials. Single-cell sequencing, and spatial transcriptomics have been utilized in clinical trials (NCT0604938) to compare the differences in tumor microenvironment components before and after treatment with CD20 CAR-T therapy for B-cell Non-Hodgkin’s Lymphoma. The use of multi-omics data to predict the therapeutic effects of drugs based on tumor molecular data can further drive targeted cancer therapy.

### New drugs based on the cancer metabolic adaptation theory

Tumors are not only genetic diseases but also metabolic disorders, as evidenced by the fact that even with ample oxygen supply, tumor cells prefer to undergo glycolysis, promoting lactic acid secretion, known as the “Warburg effect“.^467^ In 1948, Sidney Farber described how the anti-folate drug aminopterin inhibits dihydrofolate reductase, thereby blocking the synthesis of nucleic acids and proteins in acute leukemia in children. Aminopterin became the progenitor of a large class of chemotherapeutic drugs. Since that time, research on drugs targeting tumor metabolism has never ceased. Fatty acid metabolism plays a significant role in the growth and metastasis of tumor cells. Fatty acids are involved not only in the structural synthesis and signal transduction of phospholipids on the surface of cancer cell membranes but also maintain energy requirements through β-oxidation and utilize NADPH to maintain redox balance. Lu Zhimin’s team published research indicating that the gluconeogenic enzyme phosphoenolpyruvate carboxykinase 1 (PCK1) has protein kinase activity and phosphorylates protein substrates using GTP as the phosphate donor, continuously stimulating tumor lipid synthesis to promote tumor progression.^468^ These data further attracted researchers’ attention to accelerating the development of metabolic-related drugs. However, drugs that block some metabolic pathways can affect the uptake of normal cells, making the development of lipid metabolism drugs exceptionally challenging. For example, clinical development of hexokinase (HK) inhibitors such as 2-deoxyglucose has been halted due to high toxicity or low efficacy.^469^ To date, only a limited number of drugs targeting cancer metabolism have been successfully applied clinically (FDA-approved enasidenib and Ivosidenib for acute myeloid leukemia), but this situation may change as our understanding of tumors deepens in the future.

### New drugs based on the impact of gut microbiota on tumor progression

The digestive tract is a habitat for bacteria, fungi, and viruses. The occurrence and development of many cancers, as well as the efficacy of their immunotherapy, have been reported to be associated with the gut microbiota. Known intestinal carcinogenic bacteria include Salmonella typhi, which is related to gallbladder cancer, and Helicobacter spp., which is associated with primary liver cancer and gastric cancer. Among them, Helicobacter has been identified as a Group 1 carcinogen by the World Health Organization. In January 2024, Jun Yu’s team reported that, in addition to Helicobacter pylori, a non-H. pylori-driven bacterium, S. anginosus,^470^ promotes the occurrence of gastric tumors, which demonstrates the important role of carcinogenic bacteria. Furthermore, in the research on gut microbiota and immunotherapy, two pioneering studies by the teams of Laurence Zitvogel and Giorgio Trinchieri demonstrated that complete gut microbiota is crucial for activating the innate and adaptive immune systems’ effectiveness against three types of cancer therapies. Zitvogel, Gajewski, and Jennifer Wargo published three parallel studies showing that gut commensal bacteria determine the efficacy of anti-PD-1 ICIs in patients with melanoma and epithelial tumors. These results further revealed the influence of carcinogenic bacteria on the composition of primary resistance to immune therapy. In summary, recent research has shown that gut microbiota is closely related to the occurrence and progression of tumors, and studies on gut microbiota are essential for the prevention and treatment of cancer.

Overall, the future of cancer treatment will be diversified and integrated, combining traditional therapeutic methods with emerging technologies to offer patients a wider range of treatment choices. As our understanding of the complexity of cancer increases, we will be better equipped to tackle this challenge and ultimately achieve the control and cure of cancer.

## Supplementary information

Supplementary_Materials
