# Supplementary material for: Exploring treatment options in cancer: tumor treatment strategies
Source: Signal Transduct Target Ther. 2024 Jul 17;9:175. doi: 10.1038/s41392-024-01856-7 (PMC11252281; doi:10.1038/s41392-024-01856-7)
Supplement: Supplementary file 1 — Supplementary_Materials [file 41392_2024_1856_MOESM1_ESM.docx]

Supplementary Materials for

Exploring treatment options in cancer: tumor treatment strategies

Beilei Liu, Hongyu Zhou, Licheng Tan, Siu Kin To Hugo, Xin-Yuan Guan

Correspondence to: xyguan@hku.hk

**This PDF file includes:**

Tables S1 to S3

Table S1. Approved monoclonal antibodies (mAbs) for oncology treatment by Year 2024

| **Antibody Name** | **Trade Name** | **Drug Company** | **Target,** | **Medical indications** | **Year of approval** | **Clinical Efficacy** |
| --- | --- | --- | --- | --- | --- | --- |
| Trastuzumab | Herceptin | Roche | HER2; Humanized IgG1 | HER2+ breast cancer | 1998 (US); 2000 (EU) | 3-year DFS: 89.5% of mono vs. 93.8% mono+chemo; 3-year RFS: 92.4% of mono vs 95.3% mono+chemo |
| Pertuzumab | Perjeta | Roche | HER2; Humanized IgG1 | HER2+ breast cancer | 2012 (US); 2013 (EU) | ORR: 3.4% vs 17.6%; CBR:17.6% vs 41.2% PFS:7.1 weeks of mono vs 17.4 mono+trastuzumab; mean overall sruvaival: 56.5 mons for pertuzumab+Trastuzumab+chemo vs 40.8 months for Trastuzumab+chemo |
| Margetuximab | MARGENZA | MacroGenics | HER2; Chimeric IgG1 | Metastatic HER2+ breast cancer | 2020 (US); NA (EU) | Median PFS: 5.8mons of Margetuximab+chemo vs 4.9mons of trastuzumab+chemo; objective response rate: 26% vs 14% |
| Cetuximab | Erbitux | Eli Lilly | EGFR; Chimeric IgG1 | Colorectal cancer and Head and Neck Cancer | 2004 (US); 2004 (EU) | Median OS: 31mons for Cetuximab plus FOLFIRI vs 26 months for bevacizumab in KRAS WT mCRC; CRYSTAL study:OS: 23.5 cetuximab+FOLFIRI vs 20.0 months FOLFIRI alone, PFS: 9.9 v 8.4 months, response rate 57.3% v 39.7%. Head and Neck cancer: 24.4 months cetuximab+radio vs 14.9 months radio alone, os: overall survival: 49.0 months vs 29.3 months |
| Panitumumab | Vectibix | Amgen | EGFR; Human IgG2 | Colorectal cancer | 2006 (US); 2007 (EU) | Median OS: 10·4 months with panitumumab vs 10·0 months with cetuximab for KRAS WT mCRC; PARADIGM study: panitumumab +FOLFOX 37.9 months vs bevacizumab + +FOLFOX 34.3 months |
| Necitumumab | Portrazza | Eli Lilly | EGFR; Human IgG1 | Non-small cell lung cancer | 2015(US); 2016(EU) | SQUIRE trial: Median OS: 11.5 months (necitumumab+chemo) vs 9.9 months (chemo alone), Median PFS was 5.7 vs 5.5 months, the Objective response rate was 31% vs 29%. |
| Amivantamab | Rybrevant | Genmab, Janssen | EGFR, cMET; Human bispecific IgG1 | NSCLC w/ EGFR exon 20 insertion mutations | 2021 (US); 2021(EU) | CHRYSALIS: ORR was 40%, median PFS was 8.3 months; MARIPOSA-2 study: PFS: Amivantamab+chemo vs amivantamab–lazertinib–chemotherapy versus chemotherapy: 6.3 and 8.3 versus 4.2 months; ORR: 64% and 63% versus 36%. |
| Ramucirumab | Cyramza | Dyax, Eli Lilly | VEGFR2; Human IgG1 | Metastatic gastric cancer, gastrooesophageal junction adenocarcinoma, colorectal cancer, hepatocellular carcinoma, NSCLC | 2014 (US); 2014(EU) | RAINBOW study for gastric cancer: median OS: 9·6 months (Ramucirumab+chemo) vs 7.4months (placebo+chemo); REGARD study:5·2 months (Ramucirumab+chemo) vs 3.8mon (placebo+chemo) |
| Bevacizumab | Avastin | Roche | VEGF; Humanized IgG1 | Colorectal cancer, non-small cell lung cancer, HER2− breast cancer | 2004 (US); 2005(EU) | Second-line Study E3200 study for mCRC: 13.0 months (Avastin + FOLFOX4) vs 10.8 months (FOLFOX4 alone); second-line TML study: 11.2 months (Avastin + FOLFOX4) vs 9.8 months (FOLFOX4 alone) |
| Rituximab | MabThera/Rituxan | Roche | CD20; Chimeric IgG1 | follicular lymphoma, diffuse large B-cell lymphoma and chronic lymphocytic leukaemia | 1997 (US); 1998(EU) | CCL8 trial: PFS: 37.7 months; REACH: PFS: 20.6months, OS:52months |
| Obinutuzumab | Gazyva/Gazyvaro | Roche | CD20; Humanized IgG1; Glycoengineered | Chronic lymphocytic leukemia,follicular lymphoma | 2013 (US); 2014(EU) | GALLIUM trial: 7-year PFS rates of 63.4% (obinutuzumab+chemo) vs 55.7% (rituximab+chemo), OS: 88.5% vs 87.2%. |
| Ofatumumab | Arzerra | Genmab, Novartis | CD20; Human IgG1 | Chronic lymphocytic leukemia | 2009 (US); 2010(EU) | Discontinued |
| Ibritumomab tiuxetan | Zevalin | Biogen, Acrotech | CD20; Murine IgG1 | Non-Hodgkin lymphoma | 2002 (US); 2004 (EU) | ORR:80% (ibritumomab tiuxetan) vs 56% (rituximab), PFS: 11.2months versus 10.1 months |
| Tositumomab-I131 | Bexxar | Corixa | CD20; Murine IgG2a | Non-Hodgkin lymphoma | 2003 (US); NA(EU) | Discontinued |
| Mosunetuzumab | Lunsumio | Roche | CD20, CD3; Humanized bispecific IgG1 | Follicular lymphoma | 2022(US); 2022(EU) | ORR:80%, CRR:60% |
| Moxetumomab pasudotox | Lumoxiti | Innate Pharma, AstraZeneca | CD22; Murine IgG1 dsFv immunotoxin | Hairy cell leukemia | 2018 (US); 2021(EU) | NCT01829711: durable CR rate:36%, CR with HR ≥ 360 days: 33%, and overall CR : 41%. |
| Daratumumab | Darzalex | Genmab, Janssen | CD38; Human IgG1 | Multiple myeloma | 2015(US); 2016(EU) | PERSEUS trial: PFS at 4 years: 84% (Daratumumab+chemo) vs 68% (Chemo), CR: 88% vs 70% |
| Isatuximab | Sarclissa | ImmunoGen, Sanofi | CD38; Chimeric IgG1 | Multiple myeloma | 2020(US); 2020(EU) | GMMG-HD7 trial: minimal residual disease rate: 50.1% (Isatuximab+chemo) versus 35.6% (chemo) |
| Alemtuzumab | MabCampath/Campath-1H/Lemtrada | Genzyme (Sanofi) | CD52; Humanized IgG1 | Chronic myeloid leukemia | 2001(US); 2001(EU) | Median PFS:4.7-19.6months, median OS:12-35.8months, overal response rate: 31-54%, complete response rate: 2-35%. |
| Denosumab | Xgeva/Prolia | Amgen | RANKL; human IgG2 | multiple myeloma or bone metastases from solid tumors | 2010(US); 2010(EU) | ABCSG-18 trial: 5-year DFS:89.2%, 8-year DFS:80.6% |
| Ipilimumab | Yervoy | Medarex | CTLA-4; Human IgG1 | Melanoma, renal cell carcinoma, colorectal cancer | 2011(US); 2011(EU) | MDX010-20 trial: median OS: 10months. CA 184-024 trial: median OS: 11.2 (Ipilimumab+chemo) vs. 9.1 months (chemo alone); EORTC 1871 trial: PFS : 26.2 months |
| Tremelimumab | Imjudo | MedImmune (AstraZeneca) | CTLA-4; Human IgG2A | liver cancer | 2022(US); 2023(EU) | HIMALAYA trial: OS: 16.43 months (tremelimumab+ durvalumab) vs.16.56 months (durvalumab) vs.13.8 months (sorafenib) |
| Nivolumab | Opdivo | Medarex | PD1; Human IgG4 | Melanoma, non-small cell lung cancer, renal cell carcinoma | 2014(US); 2015(EU) | Melanoma RELATIVITY-047: median PFS:4.6months (nivolumab) vs 10.1 months (relatlimab–nivolumab), 1-year PFS: 47.7% vs 36.0%. Lung cancer CheckMate 816: median event-free survival: 31.6 months (nivolumab+chemo) vs 20.8 months (chemo alone). RCC NORA (NCT02940639): median OS was 24 months and median PFS was 5.3 months. |
| Pembrolizumab | Keytruda | Merck | PD1; Humanized IgG4 | Melanoma | 2014(US); 2015(EU) | NCT02362594: 5-year rate of RFS: 55.4%, 5-year rate of distant metastasis-free survival:60.6%. KEYNOTE-716: Median RFS was 37·2 months |
| Cemiplimab | Libtayo | Regeneron, Sanofi | PD-1; Human IgG4 | Cutaneous squamous cell carcinoma, basal cell carcinoma, non-small cell lung cancer | 2018(US); 2019(EU) | NCT04154943: 51% pathological CR, 13% pathological major response. EMPOWER-Lung 3 (NCT03409614): OS: 21.9 months (Cemiplimab+chemo) vs 13.0 months (chemo) |
| Dostarlimab | Jemperli | GSK | PD-1; Humanized IgG4 | Endometrial cancer | 2021(US); 2021(EU) | RUBY/ENGOT-EN6/GOG3031/NSGO: overall 2-year PFS: 36.1% (Dosatarlimab+chemo) vs 18.1% (chemo alone), overall 2-year OS: 71.3% vs 56.0% |
| Tislelizumab | TEVIMBRA | BeiGene & Novartis | PD-1; Humanized IgG4 | Esophageal squamous cell carcinoma | Pending (US); 2023(EU) | RATIONALE-306: median OS: 17·2 months (Tislelizumab+chemo) vs 10·6 months (chemo alone) |
| Toripalimab | LOQTORZI, Tuoyi | Coherus, Junshi | PD-1; Humanized IgG4 | Nasopharyneal carcinoma, esophageal squamous cell carcinoma | 2023 (US); pending (EU) | NCT03581786: median PFS of 11.7 months (Toripalimab+chemo) versus 8.0 months (chemo alone); JUPITER-06: median PFS of 5.7(Toripalimab+chemo) vs 5.5 months (chemo alone), 1-year PFS rates: 27.8% vs 6.1% |
| Retifanlimab | Zynyz | Incyte | PD-1; Humanized IgG4 | Merkel cell carcinoma | 2023 (US); pending (EU) | POD1UM-201: ORR: 46.2%, DCR: 53.8% |
| Atezolizumab | Tecentriq | Roche | PD-L1; Humanized IgG1 | Bladder cancer, non-small cell lung cancer, triple-neg. breast cancer | 2017 (US); 2016 (EU) | POPLAR: median OS = 12.6 mo (Atezolizumab) versus 9.7 mo (chemo), 4-year OS rates: 14.8% vs 8.1%; OAK: median OS = 13.3 (Atezolizumab) versus 9.8 mo (chemo), 4-year OS rates:15.5% vs 8.7% |
| Avelumab | Bavencio | EMD Serono, Pfizer | PD-L1; Human IgG1 | Merkel-cell carcinoma, urothelial carcinoma, renal cell carcinoma | 2017 (US); 2017 (EU) | JAVELIN Bladder 100 (NCT02603432): median OS: 21.4months |
| Durvalumab | Imfinzi | MedImmune | PD-L1; Human IgG1 | non-small cell lung cancer and SCLC | 2017 (US); 2018 (EU) | PACIFIC NCT02125461: median OS: 47.5 (durvalumab) v 29.1 months (plancebo), median PFS: 16.9 v 5.6 months, estimated 5-year OS rates:42.9% vs 33.4%, 5-year PFS rates: 33.1% versus 19.0% |
| Dinutuximab | Qarziba/Unituxin | United Therapeutics | GD2; Chimeric IgG1 | Neuroblastoma | 2015 (US); 2017 (EU) | NCT0274342: ORR was 26% and best response rate 37%, 3-year PFS rate and OS rate were 31% and 66% |
| Naxitamab | Danyelza | Y-mAbs | GD2; Humanized IgG1 | High-risk neuroblastoma | 2020 (US); NA (EU) | NCT03363373: ORR of 68% |
| Elotuzumab | Empliciti | Abbvie, BMS | SLAMF7; Humanized IgG1 | Multiple myeloma | 2015(US); 2016(EU) | ELOQUENT-2 (NCT01239797): median OS= 48.3 (elotuzumab plus lenalidomide/dexamethasone (ERd)) vs 39.6 months (lenalidomide/dexamethasone (Rd)) |
| Catumaxomab | Removab | Fresenius, Trion Pharma | EPCAM/CD3; Rat/mouse bispecific mAb | Malignant ascites | NA (US); 2009 review (EU) | Discontinued |
| Tafasitamab | Monjuvi, Minjuvi | MorphoSys, Incyte | CD19; Humanized IgG1 | Diffuse large B-cell lymphoma | 2020(US); 2021(EU) | NCT02399085: median duration of response was 43.9 months, the median OS was 33.5 months and the median PFS was 11.6 months |
| Blinatumomab | Blincyto | Micromet (Amgen) | CD19, CD3; Murine bispecific tandem scFv | Acute lymphoblastic leukemia | 2014(US); 2015 (EU) | TOWER(NCT02013167): median OS: 7.7 months vs 4.0 months (chemo); ALCANTARA (NCT02000427): CR: 36%. MT103-205 (NCT01471782): CR 39%. BLAST (NCT01207388): 78% minimal/residual disease rate. |
| Olaratumab | Lartruvo | Eli Lilly | PDGRFα; Human IgG1 | Soft tissue sarcoma | 2016(US); 2016(EU) | Discontinued |
| Mogamulizumab | Poteligeo | Kyowa Kirin | CCR4; Humanized IgG1 | Cutaneous T cell lymphoma | 2018(US); 2018(EU) | NCT01728805: ORR: 28.0% (Mogamulizumab) versus 4.8% (vorinostat), PFS: 7.7 months vs 3.1 months |
| Tebentafusp | Kimmtrak | Immunocore | gp100, CD3; Bispecific immunoconjugate (TCR-scFv) | Metastatic uveal melanoma | 2022(US); 2022(EU) | Median OS: 21.6 months, ORR: 11%, Median PFS: 3.4 months. |
| Teclistamab | TECVAYLI | Jannsen | BCMA, CD3; Humanized bispecific IgG4 | Multiple myeloma | 2022(US); 2022(EU) | ORR: 63.0%, MRD-negative rate:46%, median PFS: 11.3 months |
| Elranatamab | Elrexfio | Pfizer | BCMA, CD3; Humanized IgG2 | Multiple myeloma | 2023 (US); 2023(EU) | [NCT04649359: ORR of 61.0%; 35.0% ≥CR.](https://www.clinicaltrials.gov/study/NCT04649359) |
| Talquetamab | TALVEY | Janssen | G protein-coupled receptor 5D, CD3; Humanized IgG4 bispecific | Multiple myeloma | 2023 (US); 2023(EU) | NCT04586426: ORR was 84%. |
| Epcoritamab | EPKINLY | AbbVie, Genmab | CD20, CD3; Bispecific humanized IgG1 | Diffuse large B cell lymphoma | 2023 (US); 2023(EU) | NCT03625037: ORR was 63.1% and CRR was 38.9% |
| Glofitamab | Columvi | Roche | CD20, CD3e; Bispecific 2+1 IgG1 CrossMab | Diffuse large B cell lymphoma | 2023 (US); 2023(EU) | NCT03075696: 39% CR, 12-month PFS was 37% |
| Relatlimab | Opdualag (relatlimab + nivolumab combo) | Opdualag, Bristol-Myers Squibb | LAG-3; Human IgG4 | Melanoma | 2022(US); 2022(EU) | Melanoma RELATIVITY-047: median PFS:4.6months (nivolumab) vs 10.1 months (relatlimab–nivolumab), 1-year PFS: 47.7% vs 36.0%. |
| Edrecolomab | Panorex | Unknown | EpCAM; Murine IgG2a | Colorectal cancer | NA (US); 1995* (EU) | Discontinued |
| Notes: ORR: overall response rate; CRR: complete response rate; CR: complete response; PFS: progression-free survival; OS: overall survival; DFS: disease-free survival; RFS: recurrence-free survival; CBR: clinical benefit rate; mCRC: metastatic colonrectal cancer; Disease control rate: DCR; MRD: minimal residual disease | | | | | | |
| Data source: https://www.antibodysociety.org. | |  |  |  |  |  |

|  |  |  |  |  |  |
| --- | --- | --- | --- | --- | --- |

Table S2. Summarized mRNA candidates under clinical investigation for the treatment of cancer

| NCT Number | Study Title | Study Status | Brief Summary | Study Results | Conditions | Interventions | Sponsor | Phases | Study Type | Start Date | Annotation |
| --- | --- | --- | --- | --- | --- | --- | --- | --- | --- | --- | --- |
| NCT06079346 | A Study of OT-101 With mFOLFIRINOX in Patients With Advanced and Unresectable or Metastatic Pancreatic Cancer | NOT_YET_RECRUITING | The goal of this clinical study is to compare the efficacy and safety of OT-101 in combination with mFOLFIRINOX (folinic acid, 5-FU, irinotecan, oxaliplatin) to mFOLFIRINOX alone in patients with advanced and unresectable or metastatic pancreatic cancer. | NO | Pancreatic Ductal Adenocarcinoma | DRUG: OT-101\|DRUG: mFOLFIRINOX | Oncotelic Inc. | PHASE2\|PHASE3 | INTERVENTIONAL | 01/05/2024 | mRNA drug |
| NCT06307431 | A Study of Adjuvant V940 and Pembrolizumab in Renal Cell Carcinoma (V940-004) | NOT_YET_RECRUITING | The primary objective of the study is to compare V940 plus pembrolizumab to placebo plus pembrolizumab with respect to disease-free survival (DFS) as assessed by the investigator. The primary hypothesis is that V940 plus pembrolizumab is superior to placebo plus pembrolizumab with respect to DFS. | NO | Renal Cell Carcinoma | BIOLOGICAL: V940\|BIOLOGICAL: Pembrolizumab\|BIOLOGICAL: Placebo | Merck Sharp & Dohme LLC | PHASE2 | INTERVENTIONAL | 22/04/2024 | mRNA drug |
| NCT06295809 | A Study of (Neo)Adjuvant V940 and Pembrolizumab in Cutaneous Squamous Cell Carcinoma (V940-007) | NOT_YET_RECRUITING | This is a two-part (Phase 2/Phase 3) study of V940, an individualized neoantigen therapy (INT), plus pembrolizumab in participants with locally resectable advanced cutaneous squamous cell carcinoma (LA cSCC). Phase 2 has three arms V940 plus pembrolizumab given as neoadjuvant and adjuvant treatment with standard of care (SOC), standard of care (surgical resection with/without adjuvant radiation therapy (RT) only at investigator's discretion) and pembrolizumab monotherapy given as neoadjuvant and adjuvant treatment with SOC. This phase will assess the safety and efficacy of V940 in combination with pembrolizumab as neoadjuvant and adjuvant therapy in participants with resectable LA cSCC as compared to standard of care SOC only. The primary hypothesis is that V940 plus pembrolizumab with SOC is superior to SOC only with respect to event free survival (EFS) as assessed by the investigator. Phase 3 expansion will be determined by prespecified Go-No-Go decision in which 412 additional participants will be randomized to V940 plus pembrolizumab with SOC and SOC only, without changing the inclusion/exclusion criteria for the additional enrollment or study endpoints. | NO | Carcinoma, Squamous Cell\|Skin Neoplasms | BIOLOGICAL: Pembrolizumab\|BIOLOGICAL: V940\|PROCEDURE: Surgery | Merck Sharp & Dohme LLC | PHASE2\|PHASE3 | INTERVENTIONAL | 18/04/2024 | mRNA drug |
| NCT06305767 | A Study of Pembrolizumab (MK-3475) Plus V940 in Participants With Bladder Cancer Post-Radical Resection (V940-005) | NOT_YET_RECRUITING | The purpose of this study is to assess the safety and efficacy of V940 in combination with pembrolizumab (MK-3475) compared to pembrolizumab alone as an adjuvant treatment for participants with pathologic high-risk muscle-invasive urothelial carcinoma (MIUC) after radical resection. The primary study hypothesis is that V940 in combination with pembrolizumab results in a superior disease-free survival (DFS) as assessed by the investigator compared to pembrolizumab alone in participants with high-risk MIUC after radical resection. | NO | Bladder Cancer | BIOLOGICAL: Pembrolizumab\|BIOLOGICAL: V940\|OTHER: Placebo | Merck Sharp & Dohme LLC | PHASE2 | INTERVENTIONAL | 08/04/2024 | mRNA drug |
| NCT06309485 | Phase 2 Study of WGI-0301 in Combination With Sorafenib for Advanced HCC | NOT_YET_RECRUITING | The purpose of this study is to determine the MTD of WGI-0301 in combination with Sorafenib for advanced Hepatocellular Carcinoma (HCC) and assess its safety and efficacy in adults with advanced unresectable HCC who have previously received PD-1 / PD-L1 immune checkpoint inhibitors. | NO | Advanced Hepatocellular Carcinoma (HCC) | DRUG: WGI-0301 at MTD/RP2D dose IV infusion, QW\|DRUG: WGI-0301 at MTD/RP2D -1 dose IV infusion, QW\|DRUG: Sorafenib 400 mg PO, BID continuously\|DRUG: Sorafenib 400 mg PO, BID | Zhejiang Haichang Biotech Co., Ltd. | PHASE2 | INTERVENTIONAL | 01/04/2024 | mRNA drug |
| NCT06249048 | Study of IT STX-001 in Patients With Advanced Solid Tumors as Monotherapy or in Combination With Pembrolizumab | NOT_YET_RECRUITING | Phase 1/2, Open-label, Multi-center, First-in-human Study of the Safety, Tolerability, Pharmacokinetics, Pharmacodynamics and Anti-tumor Activity of STX-001 Delivered by Intratumoral Injection in Patients with Advanced Solid Tumors as a Monotherapy or in Combination with Pembrolizumab | NO | Advanced Solid Tumor | BIOLOGICAL: STX-001\|BIOLOGICAL: Keytruda | Strand Therapeutics Inc. | PHASE1\|PHASE2 | INTERVENTIONAL | 01/04/2024 | mRNA drug |
| NCT06301321 | The Evaluation and Comparison of BCR-ABL p210 mRNA Transcripts (%IS Unit) Results Between Dr. PCRBCR-ABL1 Major IS Detection Kit (Optolane) and QXDxBCR-ABL %IS Kit (Bio-Rad) in Chronic Myeloid Leukemia Patients | RECRUITING | Today, there are many commercial kits for detecting BCR-ABL fusion transcripts. The QXDxBCR-ABL %IS kit (Bio-Rad, Hercules, CA, USA) is the first ddPCR-based in vitro diagnostics (IVD) product with the US Food and Drug Administration clearance and European Conformity (CE) mark which launched in 2017. Dr. PCRBCR-ABL1 Major IS Detection Kit (Optolane, South Korea) is one of CE-IVD commercial kits based on digital RT-PCR. Both commercial kits are digital PCR-based, also evaluated their correlation, pros and cons in order for users to select a reagents kit that are appropriate for themselves. | NO | Chronic Myelogenous Leukemia | | Siriraj Hospital | | OBSERVATIONAL | 20/03/2024 | mRNA drug |
| NCT06297941 | Study of REM-422 in Patients With AML or Higher Risk MDS | NOT_YET_RECRUITING | The goal of this study is to determine the safety and antitumor effects of REM-422, a MYB mRNA degrader, in people with Higher Risk MDS and relapsed/refractory AML | NO | Myelodysplastic Syndromes\|Higher Risk Myelodysplastic Syndromes\|Acute Myeloid Leukemia\|Acute Myeloid Leukemia Refractory | DRUG: REM-422 | Remix Therapeutics | PHASE1 | INTERVENTIONAL | 15/03/2024 | mRNA drug |
| NCT06273553 | A Study in Subjects With Human Papillomavirus 16 or 18 Associated Cervical Intraepithelial Neoplasia Grade 2 or 3 | NOT_YET_RECRUITING | The purpose of this study is to to evaluate the safety, tolerability, immunogenicity, and efficacy of RG002 Injection in subjects with HPV16/18 associated Cervical Intraepithelial Neoplasia Grade 2 or 3(CIN2/3). | NO | Human Papillomavirus Associated Intraepithelial Neoplasia\|Cervical Intraepithelial Neoplasia Grade 2/3\|Human Papillomavirus Type 16 Infection\|Human Papillomavirus Type 18 Infection | BIOLOGICAL: RG002 injection | RinuaGene Biotechnology Co., Ltd. | PHASE1\|PHASE2 | INTERVENTIONAL | 01/03/2024 | mRNA drug |
| NCT05660408 | Study of RNA-lipid Particle (RNA-LP) Vaccines for Recurrent Pulmonary Osteosarcoma (OSA) | NOT_YET_RECRUITING | The investigators have shown that intravenous administration of tumor mRNA loaded lipid particles (LPs) localizes primarily to lung, transfect antigen presenting cells (APCs) and lead to an activated T cell response for induction of anti-tumor immunity. In contrast to other formulations, RNA-LPs recruit multiple arms of the immune system (i.e. innate/adaptive), and remodel the systemic/intratumoral immune milieu, which remain potent barriers for vaccine, cellular, and checkpoint inhibiting immunotherapies. After only a single RNA-LP vaccine, the bulk of systemic and intratumoral dendritic cells (DCs) in mice display an activated phenotype; these activated DCs (harvested from tumors) expand antigen specific T cell immunity. In immunologically resistant pulmonary osteosacroma murine tumor models (i.e. K7M2), RNA-LPs induce robust anti-tumor efficacy in settings where immune checkpoint inhibitors (i.e. anti-PD-L1 therapy) do not confer therapeutic benefit. We have already demonstrated safety of RNA-LPs in acute/chronic murine toxicity studies, and in client-owned canine trial.  In this study, we will investigate the safety and immunologic activity of RNA-LP vaccine in patients with recurrent pulmonary osteosarcoma. | NO | Pulmonary Osteosarcoma | BIOLOGICAL: RNA-LP vaccine | University of Florida | PHASE1\|PHASE2 | INTERVENTIONAL | 01/03/2024 | mRNA drug |
| NCT05264974 | Novel RNA-nanoparticle Vaccine for the Treatment of Early Melanoma Recurrence Following Adjuvant Anti-PD-1 Antibody Therapy | RECRUITING | The goal of this phase I trial is to evaluate the toxicity and feasibility of a tumor-specific RNA-NP vaccine in patients with stage IIB-IV melanoma who have progressed on anti-PD1 (a-PD1) adjuvant therapy. | NO | Melanoma | BIOLOGICAL: Autologous total tumor mRNA loaded DOTAP liposome vaccine | University of Florida | PHASE1 | INTERVENTIONAL | 01/02/2024 | mRNA drug |
| NCT06141369 | Treatment of Advanced Endocrine Tumor With Iindividualized mRNA Neoantigen Vaccine (mRNA-0523-L001) | RECRUITING | Treatment of advanced endocrine tumors, including adrenal corticocarcnioma (ACC), medullary thyroid carcinoma (MTC), thymic neuroendocrine tumor and pancreatic neuroendocrine tumor is challenging. Previous genomic profiling studies showed they presented a number of somatic mutations. The tumors Individualized mRNA neoantigen vaccine provide a promising solution since a significant portion of these tumors showed high quality of tumor specific neoantigen. The primary objective is to observe and evaluate the safety and tolerability of individualized mRNA neoantigen vaccine (mRNA-0523-L001) for the treatment of advanced endocrine tumors, failure of standard treatment or no standard treatment currently available. The secondary objective is to observe the preliminary efficacy of mRNA-0523-L001 for the treatment of advanced endocrine tumors, failure of standard treatment or no standard treatment currently available, including:  1. Neoantigen-specific CD4+ and CD8+ T lymphocyte responses induced by mRNA-0523-L001; 2. Objective response rate (ORR) and disease control rate (DCR) of tumors; 3. Progression-free survival (PFS). | NO | Adrenal Cortical Carcinoma\|Medullary Thyroid Cancer\|Thymic Neuroendocrine Carcinoma\|Pancreatic Neuroendocrine Tumor | BIOLOGICAL: individualized mRNA neoantigen vaccine (mRNA-0523-L001) | Shanghai Jiao Tong University School of Medicine | NA | INTERVENTIONAL | 13/01/2024 | neoantigen mRNA treatment |
| NCT06195384 | Anti-cancer Neoantigen mRNA Vaccine to Treat Solid Tumors | NOT_YET_RECRUITING | The WES and RAN-seq will be performed to identify and verify neoantigens and appropriate mRNA sequences will be verified, manufactured and protected for vaccine production by multiple in vitro and in vivo studies. Clinical studies will be performed to test anti-cancer function of the mRNA vaccine for immunotherapy of human cancer patients. In this phase I study, the safety, tolerance, and preliminary efficacy of the mRNA vaccine immunotherapy on human cancers will firstly be evaluated. | NO | Solid Tumor, Adult | BIOLOGICAL: Neoantigen mRNA Vaccine | Second Affiliated Hospital of Guangzhou Medical University | PHASE1 | INTERVENTIONAL | 01/01/2024 | neoantigen mRNA treatment |
| NCT06156267 | Study of Personalized Tumour Vaccines and a PD-L1 Blocker in Patients With Surgically Resected Pancreatic Adenocarcino | NOT_YET_RECRUITING | This is a phase 1, open-label study to evaluate the safety and tolerability of neoantigen personalized mRNA tumour vaccine combined with Adebrelimab (a PD-L1 humanized monoclonal antibody) in patients with surgically resected pancreatic adenocarcinoma. | NO | Pancreatic Cancer | DRUG: Adebrelimab\|DRUG: mRNA tumor vaccines | Fudan University | EARLY_PHASE1 | INTERVENTIONAL | 01/01/2024 | neoantigen mRNA treatment |
| NCT06256055 | Phase 1 Study of UCMYM802 Injection in Mesothelin-positive Advanced Malignant Solid Tumors | RECRUITING | This is a first-in-human, single-arm, open-label, dose escalation clinical study to evaluate the safety, tolerability, pharmacokinetic and pharmacodynamic characteristics, immunogenicity and preliminary efficacy of UCMYM802 (Circular mRNA encoding Anti-Mesothelin CAR-T) injection in patients with Mesothelin-positive advanced malignant solid tumors. | NO | Malignant Mesothelioma\|Colorectal Cancer\|Bile Duct Cancer\|Rectal Cancer\|Ovary Cancer\|Pancreatic Cancer\|Breast Cancer Female | BIOLOGICAL: UCMYM802 Injection | UTC Therapeutics Inc. | PHASE1 | INTERVENTIONAL | 01/01/2024 | mRNA drug |
| NCT06118086 | Study of REM-422 in Patients With Recurrent or Metastatic Adenoid Cystic Carcinoma | RECRUITING | The goal of this study is to determine the safety and antitumor effects of REM-422, a MYB mRNA degrader, in people with advanced Adenoid Cystic Carcinoma (ACC) | NO | Adenoid Cystic Carcinoma\|Metastatic Adenoid Cystic Carcinoma\|Recurrent Adenoid Cystic Carcinoma | DRUG: REM-422 | Remix Therapeutics | PHASE1 | INTERVENTIONAL | 20/12/2023 | mRNA drug |
| NCT06077760 | A Study of V940 Plus Pembrolizumab (MK-3475) Versus Placebo Plus Pembrolizumab in Participants With Non-small Cell Lung Cancer (V940-002) | RECRUITING | The goal of this study is to evaluate V940 plus pembrolizumab versus placebo plus pembrolizumab for the adjuvant treatment of completely resected (R0) Stage II, IIIA, IIIB (with nodal involvement \[N2\]) non-small cell lung cancer (NSCLC). The primary hypothesis is that V940 plus pembrolizumab is superior to placebo plus pembrolizumab with respect to disease-free survival (DFS) as assessed by the investigator. | NO | Non-small Cell Lung Cancer | BIOLOGICAL: V940\|BIOLOGICAL: Pembrolizumab\|OTHER: Placebo | Merck Sharp & Dohme LLC | PHASE3 | INTERVENTIONAL | 06/12/2023 | mRNA drug |
| NCT05889195 | Detection and Risk Stratification in Veterans Presenting With Microscopic Hematuria | RECRUITING | It is of current debate whether the use of invasive (referring to a process that requires insertion into the body) standard of care procedures such as a cystoscopy which is a procedure to look inside the bladder using a thin camera called a cystoscope, is appropriate for use in patients with microscopic hematuria or blood in urine invisible to the naked eye. This is because the risk of disease (bladder cancer - urothelial carcinoma) is relatively low in this population group, approximately 3%. Invasive procedures such as a cystoscopy comes with anxiety and pain, in addition to other potential side effects. This has resulted in low admittance in urology clinics for cystoscopy with hematuria (blood in urine) patients. Therefore, there is a need for a more simple, non-invasive test that can accurately detect the presence or absence of disease (urothelial carcinoma) in patients with microscopic hematuria. There is a potential role Cxbladder, a non-invasive, urine based test, can fill this role. | NO | Urothelial Carcinoma | DIAGNOSTIC_TEST: Cxbladder urine test | Pacific Edge Limited | | OBSERVATIONAL | 02/11/2023 | mRNA drug |
| NCT05799612 | Phase I Study of TH1 Dendritic Cell Immunotherapy for the Treatment of Cutaneous Angiosarcoma | NOT_YET_RECRUITING | To find the highest tolerable dose of an mRNA vaccine that can be safely given to patients with cutaneous angiosarcoma | NO | Angiosarcoma | DRUG: Paclitaxel\|BIOLOGICAL: mRNA plus Lysate-loaded Dendritic Cell Vaccine\|DRUG: PEGYLATED-INTERFERON ALPHA-2A\|DRUG: Filgrastim | M.D. Anderson Cancer Center | PHASE1 | INTERVENTIONAL | 30/09/2023 | Dendritic mRNA therapy |
| NCT06088004 | A Study to Evaluate ABO2011 Monotherapy in Advanced Solid Tumors | ENROLLING_BY_INVITATION | his is an open-label, single-arm, dose-escalation, and dose-expansion clinical study to evaluate the safety, tolerability, PK/PD, and preliminary efficacy of ABO2011 monotherapy in patients with advanced solid tumors who have progressed or metastasized after systemic standard of treatment. | NO | Solid Tumor, Adult | DRUG: ABO2011 Injection | Suzhou Abogen Biosciences Co., Ltd. | PHASE1 | INTERVENTIONAL | 28/09/2023 | mRNA drug |
| NCT06026774 | Clinical Study of Personalized mRNA Vaccine Encoding Neoantigen in Subjects With Resected Digestive System Neoplasms | RECRUITING | The purpose of this study is to assess the safety, feasibility, and efficacy of personalized mRNA vaccine iNeo-Vac-R01 with standard adjuvant therapy in subjects with surgically resected digestive system neoplasms. | NO | Digestive System Neoplasms | BIOLOGICAL: iNeo-Vac-R01 in combination with standard adjuvant therapy | Sir Run Run Shaw Hospital | PHASE1 | INTERVENTIONAL | 08/09/2023 | neoantigen mRNA treatment |
| NCT06019702 | Clinical Study of Personalized mRNA Vaccine Encoding Neoantigen Alone in Subjects With Advanced Digestive System Neoplasms | RECRUITING | The purpose of this study is to assess the safety, feasibility, and efficacy of personalized mRNA vaccine iNeo-Vac-R01 alone in subjects with advanced digestive system neoplasms. | NO | Digestive System Neoplasms | BIOLOGICAL: iNeo-Vac-R01 | Sir Run Run Shaw Hospital | PHASE1 | INTERVENTIONAL | 08/09/2023 | neoantigen mRNA treatment |
| NCT06026800 | Clinical Study of Personalized mRNA Vaccine Encoding Neoantigen in Combination With Standard First-line Treatment in Subjects With Advanced Digestive System Neoplasms | RECRUITING | The purpose of this study is to assess the safety, feasibility, and efficacy of personalized mRNA vaccine iNeo-Vac-R01 in combination with first-line treatment in subjects with advanced digestive system neoplasms. | NO | Digestive System Neoplasms | BIOLOGICAL: iNeo-Vac-R01 | Sir Run Run Shaw Hospital | PHASE1 | INTERVENTIONAL | 08/09/2023 | neoantigen mRNA treatment |
| NCT05969041 | Study of MT-302 in Adults With Advanced or Metastatic Epithelial Tumors | RECRUITING | MYE Symphony is a multicenter, open-label, Phase 1 first-in-human study to assess the safety, tolerability, and define the RP2D of MT-302 in participants with advanced epithelial cancer. | NO | Epithelial Tumors, Malignant | DRUG: MT-302 (A) | Myeloid Therapeutics | PHASE1 | INTERVENTIONAL | 02/08/2023 | mRNA drug |
| NCT04741984 | Monocyte Antigen Carrier Cells for Newly Diagnosed GBM | WITHDRAWN | The primary purpose of this study is to determine the maximum tolerated dose (MTD) of MT-201-GBM (pp65CMV antigen monocytes) that will be administered to patients newly diagnosed with a type of brain tumor called glioblastoma (GBM) that has an unmethylated MGMT (O\[6\]-methylguanine-DNA methyltransferase) (MGMT) gene promoter. | NO | Glioblastoma\|Glioma, Malignant | BIOLOGICAL: MT-201-GBM monocyte vaccine | Michael Gunn | PHASE1 | INTERVENTIONAL | 01/08/2023 | mRNA drug |
| NCT05949775 | Clinical Study of mRNA Vaccine in Patients With Advanced Malignant Solid Tumors | NOT_YET_RECRUITING | This study is an open, single arm, dose increasing study to evaluate the safety and efficacy of the combination of mRNA personalized tumor vaccine encoding neoantigen (hereinafter referred to as tumor vaccine) and Sintilimab injection (hereinafter referred to as Sintilimab) in the treatment of advanced malignant solid tumors. | NO | Advanced Malignant Solid Tumors | BIOLOGICAL: Neoantigen mRNA Personalised Cancer vaccine | Stemirna Therapeutics | NA | INTERVENTIONAL | 20/07/2023 | neoantigen mRNA treatment |
| NCT05933577 | A Clinical Study of V940 Plus Pembrolizumab in People With High-Risk Melanoma (V940-001) | RECRUITING | The purpose of this study is to learn if V940 which is an individualized neoantigen therapy (INT; formerly, called messenger ribonucleic acid \[mRNA\]-4157) with pembrolizumab (MK-3475) is safe and prevents cancer from returning in people with high-risk melanoma. Researchers want to know if V940 with pembrolizumab is better than receiving pembrolizumab alone at preventing the cancer from returning. | NO | Melanoma | BIOLOGICAL: V940\|BIOLOGICAL: Pembrolizumab\|OTHER: Placebo | Merck Sharp & Dohme LLC | PHASE3 | INTERVENTIONAL | 19/07/2023 | mRNA drug |
| NCT05981066 | A Clinical Study of mRNA Vaccine (ABOR2014/IPM511) in Patients With Advanced Hepatocellular Carcinoma | RECRUITING | This is an open label, single-site, investigator-initiated trial designed to evaluate the safety, tolerability and preliminary efficacy of ABOR2014(IPM511) injection in relapsed/ refactory HCC. | NO | Advanced Hepatocellular Carcinoma | DRUG: Neoantigen vaccine, I.M injection | Peking Union Medical College Hospital | NA | INTERVENTIONAL | 10/07/2023 | neoantigen mRNA treatment |
| NCT05942378 | A Study of HRXG-K-1939 and Adebrelimab in Patients With Advanced Solid Tumors | NOT_YET_RECRUITING | This is a Phase 1, open-label study evaluating the efficacy and safety of HRXG-K-1939 in combination with Adebrelimab (anti-programmed death-ligand 1 \[anti-PD-L1\] antibody) in patients with advanced solid tumors. HRXG-K-1939 will be administered to patients in a dose escalation regimen to determine a recommended dose for expansion. | NO | Advanced Solid Tumors | DRUG: HRXG-K-1939\|DRUG: Adebrelimab | Fudan University | PHASE1 | INTERVENTIONAL | 01/07/2023 | mRNA drug |
| NCT05938387 | Safety and Tolerability of CVGBM in Adults With Newly Diagnosed MGMT-Unmethylated Glioblastoma or Astrocytoma | ACTIVE_NOT_RECRUITING | This study is an open-label, first-in-human, dose-escalation study of CV09050101 mRNA vaccine (CVGBM) in patients with newly diagnosed "MGMT-unmethylated" Glioblastoma (GBM). Patients with isocitrate dehydrogenase (IDH)-wildtype astrocytoma with a molecular signature of "unmethylated" GBM are also eligible.  After surgical resection and completion of radiotherapy for GBM with or without chemotherapy, patients will receive CVGBM i.e. as monotherapy after radiotherapy with or without chemotherapy.  The study consists of a dose-escalation part (Part A) which completes enrollment in February 2024 and a dose-expansion part (Part B) which is anticipated to begin enrolling in June/July 2024.  Patients will receive a total of 7 administrations of CVGBM on Days 1, 8, 15, 29, 43, 57, and 71. At the discretion of the Investigator in alignment with the Sponsor's medical monitor the vaccinations may continue beyond Day 71 every 6 weeks until one year after the first CVGBM vaccination or upon disease progression or undue toxicity. | NO | Glioblastoma | BIOLOGICAL: CV09050101 mRNA vaccine (CVGBM) 12 渭g\|BIOLOGICAL: CV09050101 mRNA vaccine (CVGBM) 25 渭g\|BIOLOGICAL: CV09050101 mRNA vaccine (CVGBM) 50 渭g\|BIOLOGICAL: CV09050101 mRNA vaccine (CVGBM) 100 渭g\|BIOLOGICAL: CV09050101 mRNA vaccine RDE\|BIOLOGICAL: CV09050101 mRNA vaccine (CVGBM) 6 渭g | CureVac | PHASE1 | INTERVENTIONAL | 09/05/2023 | mRNA drug |
| NCT05761717 | Clinical Study of mRNA Vaccine in Patients With Liver Cancer After Operation | NOT_YET_RECRUITING | This is an open, one-arm study to evaluate the safety and efficacy of mRNA personalized tumor vaccine (tumor vaccine) encoding neonatal antigen in combination with Sintilimab injection for adjuvant prevention of postoperative recurrence of hepatocellular carcinoma. | NO | Posto Perative Hepatocellular Carcinoma | DRUG: Neoantigen mRNA Personalised Cancer vaccine in combination with Stintilimab I njection | Shanghai Zhongshan Hospital | NA | INTERVENTIONAL | 20/04/2023 | neoantigen mRNA treatment |
| NCT05940181 | A Safety and Efficacy Study of XH001 Combined With Sintilimab Injection in Advanced Solid Tumors | RECRUITING | This is an investigator-initiated, single-center, open label, single-arm dose escalation study of XH001 (neoantigen tumor vaccine) in combination with sintilimab for advanced solid tumors. To evaluate the safety and tolerability of XH001 combined with sintilimab in subjects with advanced solid tumors, and preliminarily evaluate the efficacy of the combination therapy in subjects with advanced solid tumors.  The study will include pre-screening period (about 12 weeks), screening period (Weeks -4 to Day 1, and Week -1 to Day -1 will be baseline period), treatment period (Day 1 to Week 16 will be combination treatment period, followed by sintilimab monotherapy), and follow-up period. After signing pre-screening informed consent, tumor tissue and blood samples will be collected for gene sequencing, neoantigen prediction and vaccine preparation. During vaccine preparation, subjects will receive sintilimab (200mg, intravenous infusion, 21-day per cycle) or other antitumor therapy as deemed appropriate by the investigator. Subjects who sign and provide formal informed consent will enter the formal screening period, and qualified subjects will enter treatment period. During the treatment period, subjects will receive 6 cycles of XH001+ sintilimab, followed by sintilimab monotherapy (sintilimab will be administered for up to 18 cycles or for 1 year, whichever comes first).  The dose escalation phase follows standard 3+3 design. 9-12 subjects are expected to be enrolled at 2 given dose level. | NO | Solid Tumor | BIOLOGICAL: XH001+ sintilimab | jianming xu | NA | INTERVENTIONAL | 01/03/2023 | mRNA drug |
| NCT05738447 | Application of mRNA Immunotherapy Technology in Hepatitis B Virus-related Refractory Hepatocellular Carcinoma | RECRUITING | The purpose of this study is to evaluate the efficacy and safety of mRNA vaccine for HBV-positive Advanced Hepatocellular Carcinoma. | NO | Liver Cancer\|Hepatocellular Carcinoma | BIOLOGICAL: HBV mRNA vaccine | West China Hospital | PHASE1 | INTERVENTIONAL | 15/02/2023 | mRNA vaccine for HBV+ HCC |
| NCT05499013 | Study to Assess SLN124 in Patients With Polycythemia Vera | RECRUITING | This is a Phase 1/2, multicenter study with an open-label dose escalation followed by a randomized placebo controlled and double-blind phase of SLN124 in adult patients with Polycythemia Vera (PV) to assess the safety, tolerability, efficacy, pharmacokinetic (PK), and Pharmacodynamic (PD) response of SLN124. | NO | Polycythemia Vera | DRUG: SLN124\|DRUG: Placebo | Silence Therapeutics plc | PHASE1\|PHASE2 | INTERVENTIONAL | 26/01/2023 | mRNA drug |
| NCT05579275 | Evaluate the Safety and Tolerability of JCXH-212 Injection in the Treatment of Advanced Malignant Solid Tumors | RECRUITING | To evaluate the safety and tolerability of JCXH-212 injection in patients with advanced malignant solid tumors; to determine the maximum tolerated dose (MTD), and to evaluate the dose-limiting toxicity (DLT) of JCXH-212 injection. | NO | Advanced Malignant Solid Tumors | BIOLOGICAL: JCXH-212 Injection | Peking University Cancer Hospital & Institute | EARLY_PHASE1 | INTERVENTIONAL | 04/01/2023 | mRNA drug |
| NCT06076265 | Quantitative Detection of CEA mRNA in the Smog and Tissue Exudate Whole Process Collected During Laparoscopic (Robotic) Radical Gastrectomy in Gastric Cancer Patients | RECRUITING | The purpose of this SMOG 01 study is to observe the possibility of intraperitoneal dissemination of tumor cells throughout the entire laparoscopic (robotic) radical gastrectomy for gastric cancer and explore its related mechanisms and potential clinical significance with peritoneal metastasis. | NO | Gastric Cancer | PROCEDURE: Radical Gastric Cancer Surgery | The Affiliated Hospital of Qingdao University | | OBSERVATIONAL | 01/12/2022 | mRNA drug |
| NCT05714748 | Application of mRNA Immunotherapy Technology in Epstein-Barr Virus-related Refractory Malignant Tumors | RECRUITING | The purpose of this study is to evaluate the efficacy and safety of mRNA vaccine for the EBV-positive Advanced Malignant Tumors. | NO | Malignant Tumors | BIOLOGICAL: EBV mRNA vaccine | West China Hospital | PHASE1 | INTERVENTIONAL | 18/11/2022 | mRNA drug |
| NCT05359354 | Safety and Efficacy of Personalized Neoantigen Vaccine in Advanced Solid Tumors | RECRUITING | This trial is an investigator-initiated, single-center, open-label, single-arm exploratory study of mRNA personalized neoantigen tumor vaccine in the treatment of advanced solid tumors, including two phases: dose escalation and dose expansion. The main objective is to evaluate the safety and tolerability of personalized neoantigen tumor vaccine in subjects with advanced solid tumors, and secondary objective is to preliminarily evaluate the efficacy of personalized neoantigen tumor vaccine in subjects with advanced solid tumors. According to the characteristics of safety and efficacy data in the dose escalation phase, the dose expansion is performed at the intended clinical dose based on the investigator's judgment, and the treatment will be performed in combination with PD-1 to further evaluate the efficacy and safety profile of personalized neoantigen tumor vaccine at a specific dose.  Both the dose escalation phase and dose expansion phase include a screening period (Week -4 \~ Week -2), a baseline period (Week -1 \~ Day -1), a treatment period (Day 1 \~ Week 8 or 16), and a follow-up period. Subjects who signed and provided the formal informed consent entered the screening period. The treatment period included the initial treatment period (Day 1 \~ Week 8) and the enhanced treatment period (Week 12 \~ Week 16). The investigator determine if the subject is suitable to enter the enhanced treatment period based on the comprehensive judgment of the subject's efficacy, safety, compliance and other factors.  Dose escalation phase is the traditional 3 + 3 design,, 12-18 subjects are expected to be enrolled at 100 渭g, 200 渭g and 400 渭g (3-6 subjects in each group). The low dose group will be enrolled first.  The investigator will choose the optimal clinical dose for dose expansion, which can be one dose group or multiple dose groups. PD-1 will be administered in parallel to further confirm the efficacy and safety of neoantigen tumor vaccine. About 18 subjects will be enrolled. The usage and dosage of PD-1 should aligned with the package insert. | NO | Solid Tumor | BIOLOGICAL: Personalized neoantigen tumor vaccine | YueJuan Cheng | NA | INTERVENTIONAL | 01/09/2022 | neoantigen mRNA treatment |
| NCT05497453 | A Phase 1/2 Study to Evaluate OTX-2002 in Patients With Hepatocellular Carcinoma and Other Solid Tumor Types Known for Association With the MYC Oncogene | RECRUITING | This is a Phase 1/2 open-label study to evaluate the safety, tolerability, pharmacokinetics, pharmacodynamics, and preliminary antitumor activity of OTX-2002 as a single agent and in combination with standard of care in patients with hepatocellular carcinoma (HCC) and other solid tumor types known for association with the MYC oncogene.  The study consists of Part 1 (OTX-2002 monotherapy) and Part 2 (OTX-2002 combined with standard of care in hepatocellular carcinoma). Part 1 consists of escalation and expansion, and Part 2 consists of safety run-in and expansion. The objective of Part 1 escalation and Part 2 safety run-in will be safety and tolerability, while anti-tumor activity will be evaluated as the primary endpoint in Part 1 and Part 2 expansion. | NO | Hepatocellular Carcinoma\|Solid Tumor\|Hepatocellular Carcinoma Non-resectable\|Hepatocellular Carcinoma Recurrent\|Hepatocellular Cancer\|Liver Cancer\|Liver, Cancer of, Non-Resectable | DRUG: OTX-2002\|DRUG: Tyrosine kinase inhibitor One\|DRUG: Tyrosine kinase inhibitor Two\|DRUG: Checkpoint Inhibitor, Immune | Omega Therapeutics | PHASE1\|PHASE2 | INTERVENTIONAL | 19/08/2022 | mRNA drug |
| NCT05497453 | A Phase 1/2 Study to Evaluate OTX-2002 in Patients With Hepatocellular Carcinoma and Other Solid Tumor Types Known for Association With the MYC Oncogene | RECRUITING | This is a Phase 1/2 open-label study to evaluate the safety, tolerability, pharmacokinetics, pharmacodynamics, and preliminary antitumor activity of OTX-2002 as a single agent and in combination with standard of care in patients with hepatocellular carcinoma (HCC) and other solid tumor types known for association with the MYC oncogene.  The study consists of Part 1 (OTX-2002 monotherapy) and Part 2 (OTX-2002 combined with standard of care in hepatocellular carcinoma). Part 1 consists of escalation and expansion, and Part 2 consists of safety run-in and expansion. The objective of Part 1 escalation and Part 2 safety run-in will be safety and tolerability, while anti-tumor activity will be evaluated as the primary endpoint in Part 1 and Part 2 expansion. | NO | Hepatocellular Carcinoma\|Solid Tumor\|Hepatocellular Carcinoma Non-resectable\|Hepatocellular Carcinoma Recurrent\|Hepatocellular Cancer\|Liver Cancer\|Liver, Cancer of, Non-Resectable | DRUG: OTX-2002\|DRUG: Tyrosine kinase inhibitor One\|DRUG: Tyrosine kinase inhibitor Two\|DRUG: Checkpoint Inhibitor, Immune | Omega Therapeutics | PHASE1\|PHASE2 | INTERVENTIONAL | 19/08/2022 | mRNA drug |
| NCT05195294 | Study of HBV-TCR T Cells (LioCyx-M) as Monotherapy or as Combination With Lenvatinib for HBV-related HCC | NOT_YET_RECRUITING | This is a single arm, open-label and multi-center Phase 1b/2 study to evaluate the safety and efficacy of autologous T-cells transfected with mRNA encoding Hepatitis-B virus (HBV)-antigen-specific T cell receptor (TCR) (LioCyx-M) as monotherapy or as combination with lenvatinib for the treatment of advanced HBV-related hepatocellular carcinoma (HCC). | NO | Hepatocellular Carcinoma\|Liver Cancer, Adult\|Liver Cell Carcinoma | BIOLOGICAL: LioCyx-M\|DRUG: Lenvatinib | Lion TCR Pte. Ltd. | PHASE1\|PHASE2 | INTERVENTIONAL | 01/06/2022 | mRNA drug |
| NCT05392699 | ABOD2011 in Patients With Advanced Solid Tumors Progressed After Standard Systemic Therapy | RECRUITING | Based on the activation and regulation of immune system by cytokines, mRNA encoding cytokines has become one of the important directions of mRNA tumor drug development. This product (ABOD2011) is a new generation mRNA product for intratumoral injection.  The primary objective of this study is to assess the safety and tolerability, of ABOD2011 in patients with advanced solid tumors that progressed after standard systemic therapy. | NO | Patients With Advanced Solid Tumors | BIOLOGICAL: human single chain IL-12 mRNA-single dose\|BIOLOGICAL: human single chain IL-12 mRNA-multiple dose | Cancer Institute and Hospital, Chinese Academy of Medical Sciences | PHASE1 | INTERVENTIONAL | 25/05/2022 | Regulation of immune cytokine |
| NCT04745403 | Redirected HBV-Specific T Cells in Patients With HBV-related HCC (SAFE-T-HBV) | RECRUITING | This is a single center, single arm and open-label study to determine the safety of mRNA modified HBV-TCR redirected T-cells and to analyze the changes in tumor microenvironment caused by these HBV-TCR redirected T-cells in subjects with HBV-related HCC who are not amenable to/failed conventional treatment. | NO | Hepatocellular Carcinoma | DRUG: mRNA HBV/TCR T-cells | Lion TCR Pte. Ltd. | PHASE1 | INTERVENTIONAL | 20/05/2022 | mRNA vaccine for HBV+ HCC |
| NCT05456165 | Study of an Individualized Vaccine Targeting Neoantigens in Combination With Immune Checkpoint Blockade for Patients With Colon Cancer | TERMINATED | The primary objective is to assess and characterize the antitumor activity and safety and tolerability of adjuvant treatment with an individualized neoantigen vaccine called GRT-C901/GRT-R902 (chimpanzee adenovirus \[ChAd\] and self-amplifying messenger RNA \[samRNA\] vectors), in combination with checkpoint inhibitors. Antitumor activity will be based on molecular response in patients with colon cancer who have circulating tumor deoxyribonucleic acid (ctDNA) following surgical resection. | NO | Colonic Neoplasms\|Colorectal Neoplasms | DRUG: GRT-C901\|DRUG: GRT-R902\|DRUG: Atezolizumab\|DRUG: Ipilimumab\|DRUG: Adjuvant chemotherapy | Gritstone bio, Inc. | PHASE2 | INTERVENTIONAL | 19/05/2022 | neoantigen mRNA treatment |
| NCT05302037 | Allogeneic NKG2DL-targeting CAR 纬未 T Cells (CTM-N2D) in Advanced Cancers | UNKNOWN | CAR-T is a pioneering cancer treatment which has found success in some cancers. This treatment is made first by taking blood cells from the patient. Then in the lab, an artificial protein - a Chimeric Antigen Receptor (CAR), is grafted on the surface of immune cells. The modified cells, which are readministered to the patient, have enhanced abilities to target and destroy cancers than unmodified immune cells.  Currently approved CAR-T can only be used autologously. i.e. the patient will receive CAR-T treatment made from their own cells. This is because current CAR-T treatment uses 伪尾 T cells - a type of immune cell which are largely non-transferable between individual human beings due to the high risk of Graft-versus-Host Disease. However, autologous CAR-T comes with many limitations. A lengthy, manufacturing process follows after the patient donates their own blood, accompanied by a high risk of manufacturing failure, which can be attributed to the cell quality from cancer patients undergoing stressful anti-cancer therapy.  CytoMed Therapeutics pioneers a new CAR-T treatment (CTM-N2D) which may confer some benefit over current CAR-T treatment. CTM-N2D uses a subtype of immune cell -- 纬未 T cell. Secondly, the CAR on CTM-N2D targets a surface antigen called NKG2DL which are commonly present in many cancer. These two features may confer a safer product profile, of better quality and may be efficacious in cancers where previous CAR-T treatments has not.  The phase I clinical trial of CTM-N2D will be conducted at the National University Hospital, Singapore. The objective of this clinical trial is to determine the optimal dose of CTM-N2D, and to investigate its safety and tolerability. The subjects of the clinical trial will also be investigated for their tumour response to CTM-N2D.  CTM-N2D has undergone preclinical studies. Relevant data from other clinical trials are also used to infer the expected outcome, and strategies of management of this clinical trial. The institution's ethical review board must give its approval before the study may begin. An independent Data Safety Monitoring Board monitors the safety aspect of this trial. | NO | Cancer\|Malignancy\|Refractory Cancer\|Relapsed Cancer | BIOLOGICAL: Allogeneic NKG2DL-targeting Chimeric Antigen Receptor-grafted 纬未 T Cells (CTM-N2D) | CytoMed Therapeutics Pte Ltd | PHASE1 | INTERVENTIONAL | 01/04/2022 | mRNA drug |
| NCT05192460 | Safety and Efficacy of Personalized Neoantigen Vaccine in Advanced Gastric Cancer, Esophageal Cancer and Liver Cancer | RECRUITING | This trial is an investigator-initiated, single-center, open-label, single-arm exploratory study of mRNA neoantigen tumor vaccine in the treatment of advanced gastric cancer, esophageal cancer, and liver cancer, including two phases: dose escalation and dose expansion. To evaluate the safety and tolerability of neoantigen tumor vaccine in subjects with advanced gastric cancer, esophageal cancer and liver cancer by conducting dose escalation trial in subjects diagnosed with advanced gastric cancer, esophageal cancer and liver cancer, and preliminarily evaluate the efficacy of neoantigen tumor vaccine in subjects with advanced gastric cancer, esophageal cancer and liver cancer. According to the characteristics of safety and efficacy data in the dose escalation phase, the dose expansion is performed at the intended clinical dose based on the investigator's judgment, and the treatment is performed in combination with PD-1/L1 to further evaluate the efficacy and safety profile of neoantigen tumor vaccine at a specific dose.  Both the dose escalation phase and dose expansion phase include a screening period (Week -4 \~ Week -2), a baseline period (Week -1 \~ Day -1), a treatment period (Day 1 \~ Week 8 or 16), and a follow-up period. Subjects who signed and provided the formal informed consent entered the screening period. The treatment period included the initial treatment period (Day 1 \~ Week 8) and the enhanced treatment period (Week 12 \~ Week 16). The investigator determined whether to enter the enhanced treatment period based on the comprehensive judgment of the subject's efficacy, safety, compliance and other factors from Week 8 to Week 12.  The dose escalation phase follows standard 3+3 design. 12-18 subjects are expected to be enrolled at 3 given dose level.  The investigator will choose the optimal clinical dose for dose expansion, which can be one dose group or multiple dose groups. PD-1/L1 drugs are used in parallel to further confirm the efficacy and safety of neoantigen tumor vaccine, with about 18 subjects. The usage and dosage of PD-1/L1 should aligned with the package insert. | NO | Gastric Cancer\|Esophageal Cancer\|Liver Cancer | BIOLOGICAL: neoantigen tumor vaccine with or without PD-1/L1 | jianming xu | NA | INTERVENTIONAL | 28/03/2022 | neoantigen mRNA treatment |
| NCT05198752 | A Study of Neoantigen mRNA Personalised Cancer in Patients With Advanced Solid Tumors | RECRUITING | This is a Phase 1 open label study to evaluate the tolerability, safety, immunogenicity, and efficacy of SW1115C3, a neoantigen mRNA personalised cancer vaccine, in patients with advanced malignant solid tumours. | NO | Solid Tumor | DRUG: Neoantigen mRNA Personalised Cancer SW1115C3 | Stemirna Therapeutics | PHASE1 | INTERVENTIONAL | 18/03/2022 | neoantigen mRNA treatment |
| NCT05169489 | A Study of bbT369 in Relapsed and/or Refractory B Cell Non-Hodgkin's Lymphoma (NHL) | RECRUITING | A Phase 1/2 Study of bbT369, a dual targeting CAR T cell drug product with a gene edit, in Relapsed and/or Refractory B cell Non-Hodgkin's Lymphoma. | NO | Diffuse Large B Cell Lymphoma (DLBCL) | BIOLOGICAL: bbT369 | 2seventy bio | PHASE1\|PHASE2 | INTERVENTIONAL | 24/01/2022 | mRNA drug |
| NCT04683939 | Safety, Pharmacokinetics, Pharmacodynamics, and Preliminary Efficacy Trial of BNT141 in Patients With Unresectable or Metastatic CLDN18.2-positive Gastric, Pancreatic, Ovarian and Biliary Tract Tumors | TERMINATED | This trial is an open-label, multi-site, Phase I/IIa dose escalation, safety, and pharmacokinetic (PK) trial of BNT141 followed by expansion cohorts in patients with CLDN18.2-positive tumors.  The trial design consists of three parts:  Part 1A is a dose escalation of BNT141 as monotherapy in patients with advanced unresectable or metastatic Claudin 18.2 (CLDN18.2)-positive solid tumors for which there is no available standard therapy likely to confer clinical benefit, or the patient is not a candidate for such available therapy. The dose of BNT141 will be escalated until the maximum tolerated dose (MTD) and/or recommended phase II dose (RP2D) of BNT141 as monotherapy are defined. Eligible tumor types are gastric cancer, gastroesophageal junction (GEJ) and esophageal adenocarcinoma, pancreatic, biliary tract (cholangiocarcinoma and gallbladder cancer), and mucinous ovarian cancers. Additionally, patients with specific tumors (including colorectal cancer, non-small-cell lung cancer, gastric subtype of endocervical adenocarcinoma) where there is scientific evidence that the CLDN18.2 could be elevated can be tested for CLDN18.2 expression.  Part 1B is a dose escalation of BNT141 in combination with nab-paclitaxel and gemcitabine in patients with advanced unresectable or metastatic CLDN18.2-positive pancreatic adenocarcinoma or cholangiocarcinoma who are eligible for treatment with nab-paclitaxel and gemcitabine. Part 1B intends to define the MTD and/or RP2D of the combination.  Part 2 with adaptive design elements will be added at a later stage. | NO | Solid Tumor\|Gastric Cancer\|Gastroesophageal Junction Adenocarcinoma\|Esophageal Adenocarcinoma\|Pancreatic Cancer\|Biliary Tract Cancer\|Cholangiocarcinoma\|Metastatic Cancer | BIOLOGICAL: BNT141\|DRUG: Nab-paclitaxel\|DRUG: Gemcitabine | BioNTech SE | PHASE1\|PHASE2 | INTERVENTIONAL | 18/01/2022 | mRNA drug |
| NCT04573140 | A Study of RNA-lipid Particle (RNA-LP) Vaccines for Newly Diagnosed Pediatric High-Grade Gliomas (pHGG) and Adult Glioblastoma (GBM) | RECRUITING | The primary objective will be to demonstrate the manufacturing feasibility and safety, and to determine the maximum tolerated dose (MTD) of RNA-LP vaccines in (Stratum 1) adult patients with newly diagnosed GBM (MGMT unmethylated). Funding Source - FDA OOPD | NO | Adult Glioblastoma | BIOLOGICAL: Autologous total tumor mRNA and pp65 full length (fl) lysosomal associated membrane protein (LAMP) mRNA loaded DOTAP liposome vaccine administered intravenously (RNA loaded lipid particles, RNA-LPs) | University of Florida | PHASE1 | INTERVENTIONAL | 26/10/2021 | mRNA drug |
| NCT04981691 | Anti-mesothelin CAR-T Cells With Advanced Refractory Solid Tumors | UNKNOWN | The goal of this clinical trial is to study the safety, efficacy, and pharmacokinetics of mRNA-engineered anti-Mesothelin (MESO) Chimeric Antigen Receptor T-Cell (CAR-T cells) therapy in patients with mesothelin expression-positive, advanced solid tumors that have failed at least first-line or second-line therapy. | NO | Refractory Malignant Solid Neoplasm | BIOLOGICAL: anti-MESO CAR T cells | Ruijin Hospital | PHASE1 | INTERVENTIONAL | 01/10/2021 | mRNA drug |
| NCT04837547 | PEACH TRIAL- Precision Medicine and Adoptive Cellular Therapy | RECRUITING | A Phase I open-label, multicenter study, to evaluate the safety, feasibility, and maximum tolerated dose (MTD) of treating children with newly diagnosed DIPG or recurrent neuroblastoma with molecular targeted therapy in combination with adoptive cell therapy (Total tumor mRNA-pulsed autologous Dendritic Cells (DCs) (TTRNA-DCs), Tumor-specific ex vivo expanded autologous lymphocyte transfer (TTRNA-xALT) and Autologous G-CSF mobilized Hematopoietic Stem Cells (HSCs)). | NO | Neuroblastoma\|Diffuse Intrinsic Pontine Glioma | BIOLOGICAL: Tumor-specific ex vivo expanded autologous lymphocyte transfer (TTRNA-xALT) | Giselle Sholler | PHASE1 | INTERVENTIONAL | 20/09/2021 | Dendritic mRNA therapy |
| NCT04911621 | Adjuvant Dendritic Cell Immunotherapy for Pediatric Patients With High-grade Glioma or Diffuse Intrinsic Pontine Glioma | ACTIVE_NOT_RECRUITING | Childhood aggressive gliomas are rare brain tumors with very poor prognosis. Due to the tumor's location and infiltrative nature, surgical removal is not always possible, and even when resection is performed and combined with chemo- and/or radiotherapy, tumor cells frequently persist, eventually giving rise to tumor recurrence. A promising strategy to eradicate persisting tumor cells is vaccination with dendritic cells (DC). DC are immune cells that play an important role in organizing the body's defense against cancer. The goal of DC vaccination is to activate these natural anti-tumor defense mechanisms to delay or prevent tumor progression or recurrence. Previous clinical studies have demonstrated that DC vaccination is well-tolerated, safe and capable of eliciting tumorspecific immunity.  A clinical study including 10 pediatric patients (aged 鈮?12 months and \< 18 years at the time of signing the informed consent) with brain (stem) tumors is initiated at the Antwerp University Hospital to investigate intradermal vaccination with WT1 mRNA-loaded autologous monocyte-derived DCs, either combined with first-line chemoradiation treatment or administered as adjuvant therapy following previous therapies. The general objective of this phase I/II clinical study is (1) to demonstrate that WT1-targeted DC vaccine production and administration in pediatric patients with HGG and DIPG, either combined with first-line chemoradiation treatment or administered as adjuvant therapy following previous therapies, is feasible and safe, (2) to study vaccine-induced immune responses, (3) to document patients' quality of life and clinical outcome for comparison with current patients' outcome allowing indication of the added value. | NO | High Grade Glioma\|Diffuse Intrinsic Pontine Glioma | BIOLOGICAL: Dendritic cell vaccination + temozolomide-based chemoradiation\|BIOLOGICAL: Dendritic cell vaccination +- conventional next-line treatment | University Hospital, Antwerp | PHASE1\|PHASE2 | INTERVENTIONAL | 10/09/2021 | Dendritic mRNA therapy |
| NCT05016622 | Booster Dose Trial | RECRUITING | The goal of this study is to assess the safety and effectiveness of a COVID vaccine booster in patients with cancer who have not developed an antibody after the U.S. Food and Drug Administration (FDA) Emergency Use Authorized COVID vaccination series | NO | Cancer | BIOLOGICAL: BNT162b2 vaccine | Montefiore Medical Center | PHASE2 | INTERVENTIONAL | 10/08/2021 | mRNA drug |
| NCT04844983 | A Study to Evaluate Safety, Efficacy of Intralesional Injection of STP705 in Patients With isSCC | COMPLETED | The purpose of this trial is to evaluate safety, tolerability and efficacy of various doses of STP705 administered as intralesional injection in subjects with cutaneous squamous cell carcinoma (in situ) skin cancer (isSCC). | NO | Squamous Cell Carcinoma in Situ | DRUG: STP705\|OTHER: Placebo Saline | Sirnaomics | PHASE2 | INTERVENTIONAL | 18/05/2021 | mRNA drug |
| NCT04534205 | A Clinical Trial Investigating the Safety, Tolerability, and Therapeutic Effects of BNT113 in Combination With Pembrolizumab Versus Pembrolizumab Alone for Patients With a Form of Head and Neck Cancer Positive for Human Papilloma Virus 16 and Expressing the Protein PD-L1 | RECRUITING | An open-label, controlled, multi-site, interventional, 2-arm, Phase II trial of BNT113 in combination with pembrolizumab vs pembrolizumab monotherapy as first line treatment in patients with unresectable recurrent or metastatic HPV16+ HNSCC expressing programmed cell death ligand -1 (PD-L1) with combined positive score (CPS) 鈮?.  This trial has two parts.  Part A, an initial non-randomized Safety Run-In Phase to confirm the safety and tolerability at the selected dose range level of BNT113 in combination with pembrolizumab.  Part B, the Randomized part of the trial to generate pivotal efficacy and safety data of BNT113 in combination with pembrolizumab versus pembrolizumab monotherapy in the first line setting in patients with unresectable recurrent or metastatic HPV16+ HNSCC expressing PD-L1 with CPS 鈮?.  For Part B, an optional pre-screening phase is available for all patients where patients' tumor samples may be submitted for central HPV16 DNA and central PD-L1 expression testing prior to screening into the main trial. | NO | Unresectable Head and Neck Squamous Cell Carcinoma\|Metastatic Head and Neck Cancer\|Recurrent Head and Neck Cancer | BIOLOGICAL: BNT113\|BIOLOGICAL: Pembrolizumab | BioNTech SE | PHASE2 | INTERVENTIONAL | 07/01/2021 | mRNA drug |
| NCT04286672 | Metadherin mRNA Expression in Bladder Cancer | UNKNOWN | The study aims to study alterations of MTDH gene expression in the serum of bladder cancer patients compared to control group to evaluate its role as a marker for diagnosis.  ,to compare the diagnostic accuracy of MTDH with the previously used marker Bladder Cancer-Specific Antigen-1 (BLCA-1 in the serum of bladder cancer patients .  and to study correlation between expression of the metadherin gene and serum level of BLCA-1, and clinical and histopathological staging in patients with bladder cancer | NO | Bladder Cancer | DIAGNOSTIC_TEST: Metadherin | Assiut University | | OBSERVATIONAL | 01/12/2020 | mRNA drug |
| NCT03688178 | DC Migration Study to Evaluate TReg Depletion In GBM Patients With and Without Varlilumab | ACTIVE_NOT_RECRUITING | Patients with newly diagnosed glioblastoma will be consented following tumor resection then undergo leukapheresis for harvest of peripheral blood leukocytes for generation of dendritic cells. Subjects will then receive standard of care (planned 6 weeks) radiation therapy (RT) and concurrent temozolomide (TMZ) at a standard targeted dose of 75 mg/m2/day.  The study cycle of TMZ comprises a targeted dose of 150-200mg/m2/day for 5 days every 4 (+2) weeks for up to 12 cycles (patients with unmethylated MGMT gene promoter will receive only cycle 1). All patients will receive up to a total of 10 DC vaccines called pp65 CMV dendritic cells (DC). Dendritic Cell (DC) vaccines #1-3 will be given every two weeks, thus delaying the initiation of TMZ cycle 2 for patients receiving TMZ. All remaining TMZ/vaccine cycles will be 4 (+2) weeks in length.  After the first 3 DC vaccines given during Cycle 1 of TMZ, the remaining DC vaccine injections are given on Day 21 (+/- 2 days) of each TMZ cycle. Subjects with unmethylated MGMT will only receive one cycle of adjuvant TMZ; however, their vaccine schedule will follow the same 4 (+ 2) week TMZ cycle schedule.  Following RT, patients will be randomized into 1 of 3 groups. Groups 1 and 2 will be blinded. The groups differ in the type of pre-conditioning received prior to DC vaccine #4; additionally, Group 3 will be receiving infusions of varlilumab 7 days prior to and with vaccine #1 and 7 days prior to vaccine #3+. The pre-conditioning for each group is as follows: Group 1: Unpulsed DC pre-conditioning prior to DC vaccine #4; Group 2: Tetanus-diphtheria (Td) pre-conditioning prior to DC vaccine #4; Group 3: Td pre-conditioning prior to DC vaccine #4 and varlilumab infusion at 7 days prior to each DC vaccine (except DC vaccine #2) with Td pre-conditioning prior to vaccine #4. | NO | Glioblastoma | BIOLOGICAL: Human CMV pp65-LAMP mRNA-pulsed autologous DCs\|DRUG: Temozolomide\|BIOLOGICAL: Varlilumab\|BIOLOGICAL: Td\|BIOLOGICAL: Unpulsed DCs | Annick Desjardins, MD | PHASE2 | INTERVENTIONAL | 26/08/2020 | Dendritic mRNA therapy |
| NCT04423549 | Predictive Value of a mRNA Signature and Liquid Biopsy in Diffuse Large B Cell Lymphoma | UNKNOWN | The purpose of this study is to determine the predictive value of a mRNA signature and liquid biopsy in patients with Diffuse Large B cell lymphoma. | NO | Diffuse Large B Cell Lymphoma | | Sun Yat-sen University | | OBSERVATIONAL | 01/06/2020 | mRNA drug |
| NCT04382898 | PRO-MERIT (Prostate Cancer Messenger RNA Immunotherapy) | TERMINATED | Open-label, multicenter, dose titration and four-arm expansion trial to evaluate the safety, tolerability, immunogenicity, and preliminary efficacy of BNT112 cancer vaccine (BNT112) monotherapy or in combination with cemiplimab in patients with metastatic castration resistant prostate cancer (mCRPC: Part 1 and Part 2 Arms 1A and 1B) and in patients with high-risk, localized prostate cancer (LPC).  As of February 2023, the trial will be only recruiting LPC patients and no longer mCRPC patients. | NO | Prostate Cancer | BIOLOGICAL: BNT112\|DRUG: Cemiplimab | BioNTech SE | PHASE1\|PHASE2 | INTERVENTIONAL | 19/12/2019 | mRNA drug |
| NCT04382898 | PRO-MERIT (Prostate Cancer Messenger RNA Immunotherapy) | TERMINATED | Open-label, multicenter, dose titration and four-arm expansion trial to evaluate the safety, tolerability, immunogenicity, and preliminary efficacy of BNT112 cancer vaccine (BNT112) monotherapy or in combination with cemiplimab in patients with metastatic castration resistant prostate cancer (mCRPC: Part 1 and Part 2 Arms 1A and 1B) and in patients with high-risk, localized prostate cancer (LPC).  As of February 2023, the trial will be only recruiting LPC patients and no longer mCRPC patients. | NO | Prostate Cancer | BIOLOGICAL: BNT112\|DRUG: Cemiplimab | BioNTech SE | PHASE1\|PHASE2 | INTERVENTIONAL | 19/12/2019 | mRNA drug |
| NCT04382898 | PRO-MERIT (Prostate Cancer Messenger RNA Immunotherapy) | TERMINATED | Open-label, multicenter, dose titration and four-arm expansion trial to evaluate the safety, tolerability, immunogenicity, and preliminary efficacy of BNT112 cancer vaccine (BNT112) monotherapy or in combination with cemiplimab in patients with metastatic castration resistant prostate cancer (mCRPC: Part 1 and Part 2 Arms 1A and 1B) and in patients with high-risk, localized prostate cancer (LPC).  As of February 2023, the trial will be only recruiting LPC patients and no longer mCRPC patients. | NO | Prostate Cancer | BIOLOGICAL: BNT112\|DRUG: Cemiplimab | BioNTech SE | PHASE1\|PHASE2 | INTERVENTIONAL | 19/12/2019 | mRNA drug |
| NCT04163094 | Ovarian Cancer Treatment With a Liposome Formulated mRNA Vaccine in Combination With (Neo-)Adjuvant Chemotherapy | TERMINATED | This is a first-in-human, open label phase I study in ovarian cancer patients with primary disease eligible for standard-of-care treatment with neo-adjuvant chemotherapy, i.e. 3 cycles carboplatin/paclitaxel, interval surgery and 3 additional cycles carboplatin/paclitaxel. Eight doses of the W_ova1 vaccine will be administered prior and in combination with the (neo-)adjuvant chemotherapy to induce an anti-tumor immune response. Systemic immune responses are determined using peripheral blood mononuclear cells collected before, during and after vaccinations. Intratumoral accumulation of T-cells recognizing vaccine-encoded TAAs will be determined before vaccination in a tumor biopsy and after the 3 cycles of chemotherapy and the 5th vaccination using tumor tissue derived from interval surgery. \[18F\]FB-IL2 PET-CT will be used for the non-invasive assessment of T-cell activation and correlated to immunohistochemistry tumor tissue data from pre-treatment biopsy and interval debulking surgery | NO | Ovarian Cancer | DRUG: W_ova1 Vaccine | University Medical Center Groningen | PHASE1 | INTERVENTIONAL | 25/11/2019 | mRNA drug |
| NCT04943380 | Efficacy and Utility of Cxbladder Tests in Hematuria Patients | ACTIVE_NOT_RECRUITING | Prospective observational study to validate the performance characteristics and clinical utility of Cxbladder tests in a Veterans Affairs cohort. | NO | Hematuria\|Urothelial Carcinoma | DIAGNOSTIC_TEST: Cxbladder | Pacific Edge Limited | | OBSERVATIONAL | 08/11/2019 | mRNA drug |
| NCT04100733 | Surveillance of High-grade Non-muscle Invasive Bladder Tumours Using the Xpert Bladder Cancer Monitor | ACTIVE_NOT_RECRUITING | The study aims to evaluate the potential clinical impact of a highly sensitive urinary marker, the Xpert Bladder Cancer Monitor, regarding possible reduction in number of flexible cystoscopies in an outpatient setting without decreasing recurrence-free survival or increasing risk of progression. | NO | Non-muscle Invasive Bladder Cancer\|Urinary Biomarker | DIAGNOSTIC_TEST: Xpert Bladder Cancer Monitor\|DIAGNOSTIC_TEST: Flexible cystoscopy (WL/NBI) and urinary cytology | J酶rgen Bjerggaard Jensen | NA | INTERVENTIONAL | 01/11/2019 | mRNA drug |
| NCT03908671 | Clinical Study of Personalized mRNA Vaccine Encoding Neoantigen in Patients With Advanced Esophageal Cancer and Non-small Cell Lung Cancer | RECRUITING | A single arm, open-label pilot study is designed to determine the safety, tolerability and effectiveness of personalized mRNA tumor vaccine encoding neoantigen in Patients with advanced esophageal cancer and non-small cell lung cancer | NO | Esophageal Cancer\|Non Small Cell Lung Cancer | BIOLOGICAL: Personalized mRNA Tumor Vaccine | Stemirna Therapeutics | NA | INTERVENTIONAL | 18/10/2019 | neoantigen mRNA treatment |
| NCT03927222 | Immunotherapy Targeted Against Cytomegalovirus in Patients With Newly-Diagnosed WHO Grade IV Unmethylated Glioma | TERMINATED | This single-arm phase II study will assess the impact of tetanus pre-conditioning and adjuvant Granulocyte Macrophage Colony Stimulating Factor (GM-CSF) on overall survival of patients newly diagnosed with World Health Organization (WHO) Grade IV glioblastoma who have undergone definitive tumor resection, are cytomegalovirus (CMV) positive and unmethylated, and completed standard temozolomide (TMZ) and radiation treatment. After completion of the standard of care radiotherapy with concurrent TMZ, patients will receive 1 cycle of dose-intensified TMZ followed by pp65-loaded dendritic cell (DC) vaccination beginning on day 23. | NO | Glioblastoma | BIOLOGICAL: Human CMV pp65-LAMP mRNA-pulsed autologous DCs containing GM CSF\|DRUG: Temozolomide\|BIOLOGICAL: Tetanus-Diphtheria Toxoid (Td)\|BIOLOGICAL: GM-CSF\|BIOLOGICAL: 111-Indium-labeling of Cells for in vivo Trafficking Studies | Mustafa Khasraw, MBChB, MD, FRCP, FRACP | PHASE2 | INTERVENTIONAL | 30/09/2019 | Dendritic mRNA therapy |
| NCT03897881 | An Efficacy Study of Adjuvant Treatment With the Personalized Cancer Vaccine mRNA-4157 and Pembrolizumab in Participants With High-Risk Melanoma (KEYNOTE-942) | RECRUITING | The purpose of this study is to assess whether postoperative adjuvant therapy with mRNA-4157 and pembrolizumab improves recurrence free survival (RFS) compared to pembrolizumab alone in participants with complete resection of cutaneous melanoma and a high risk of recurrence. | NO | Melanoma | DRUG: mRNA-4157\|BIOLOGICAL: Pembrolizumab | ModernaTX, Inc. | PHASE2 | INTERVENTIONAL | 18/07/2019 | mRNA drug |
| NCT03948763 | A Study of mRNA-5671/V941 as Monotherapy and in Combination With Pembrolizumab (V941-001) | COMPLETED | This study will determine the safety and tolerability and establish a preliminary recommended Phase 2 dose of V941(mRNA-5671/V941) as a monotherapy and in combination with pembrolizumab infusion. | NO | Neoplasms\|Carcinoma, Non-Small-Cell Lung\|Pancreatic Neoplasms\|Colorectal Neoplasms | BIOLOGICAL: V941\|BIOLOGICAL: Pembrolizumab | Merck Sharp & Dohme LLC | PHASE1 | INTERVENTIONAL | 26/06/2019 | mRNA drug |
| NCT03638427 | Screening for Cervical Intraepithelial Neoplasia Using Self-collected Menstrual Blood | ENROLLING_BY_INVITATION | The purpose of this study is to investigate the feasibility and efficacy of assessing HR-HPV DNA and HPV E6/E7 mRNA via self-collected menstrual blood in a smart menstrual pad. In other words, can the investigators detect the high risk strains of the human papilloma virus (HPV) that are associated with cervical cancer in self-collected menstrual blood, as an alternative to collecting vaginal swabs. | NO | HPV - Anogenital Human Papilloma Virus Infection | DIAGNOSTIC_TEST: Menstrual Blood Analysis (Menstrual Blood Analysis) | Stanford University | | OBSERVATIONAL | 01/01/2019 | mRNA drug |
| NCT03673202 | The Cxbladder Rule-out of Recurrent Urothelial Carcinoma | UNKNOWN | This observational study is designed to collect urine and relevant clinical information from patients who have a known diagnosis of bladder cancer and currently on clinically driven surveillance. The study aims to compare the urine test to the flexible cystoscopy procedure (which the patient is already scheduled). | NO | Bladder Cancer | DIAGNOSTIC_TEST: Cx bladder Monitor test | London Health Sciences Centre | | OBSERVATIONAL | 26/11/2018 | mRNA drug |
| NCT03788083 | Intratumoral TriMix Injections in Early Breast Cancer Patients | RECRUITING | Patients with early breast cancer and accessible tumor lesions (1.00 to 10 ml volume) that are eligible to either surgical removal of their tumor or neoadjuvant chemotherapy will be injected with the IMP. Patients will be either treated with placebo (buffer alone, 12 patients) or with TriMix mRNA at three dose levels \[8 at dose level I (1mg/ml), 8 at dose level II (3mg/ml), and 8 at dose level III (6mg/ml). The volume injected in this group will be adjusted to the tumour volume to ensure a perfusion of around 33% of the tumour volume (33% +/- 5%). Therefore, depending on the patients' tumour size, 500, 1000 or 2000 l of TriMix mRNA solution or placebo solution will be injected into each tumor. Each patient will receive three administrations of TriMix prior to start of general treatment (surgery or neoadjuvant chemotherapy) separated by one week (7 days +/- 2 days) interval. The last administration will be performed 2 days preoperatively or start of neoadjuvant chemotherapy.  The tumor and peripheral blood samples will be analyzed for immunological changes. If it is decided by the multidisciplinary team that neoadjuvant therapy is more appropriate for the patient, a second tumor biopsy (instead of surgical resection) will be taken 2 days after third administration of TriMix mRNA to assess immunological changes within the tumor. Similarly, patients that refuses to undergo surgery or to receive neoadjuvant chemotherapy can be enrolled into the trial, if they accept three administrations of TriMix followed by a second tumor biopsy.  The study will start with recruitment of the placebo group. The enrollment of the first three patients in each cohort with Trimix mRNA will be staggered with at least one day between the first dose of each individual patient. One week after the third patient of a cohort received the third TriMix mRNA administration, an overall evaluation of the safety and tolerability of this cohort will be done by the principal investigator. The results will be reviewed by an in-house dose evaluation committee overseeing the safety and tolerability of TriMix mRNA. | NO | Breast Cancer Female\|Early-stage Breast Cancer | DRUG: Trimix\|DRUG: Placebo | Universitair Ziekenhuis Brussel | PHASE1 | INTERVENTIONAL | 12/11/2018 | mRNA drug |
| NCT03634683 | A Study of LioCyx in Patient With Recurrent HBV-related HCC Post Liver Transplantation | WITHDRAWN | Hepatocellular carcinoma (HCC) represent approximately 70-85% of liver cancer, in which Hepatitis B virus (HBV) is the major etiologic agent accounting for at least 80% of HCC in Asian countries. Overall, transplantation remains the best option however, HCC recurrence rate is high among liver transplant patients.  While there are limited treatment measures for HBV-related HCC recurrences, the study hypothesized that LioCyx is capable of lysing target liver cells expressing the HBV cognate antigens and provide clinical benefit to patients with HBV-related HCC. | NO | Recurrent Hepatocellular Carcinoma | BIOLOGICAL: LioCyx | Lion TCR Pte. Ltd. | PHASE1\|PHASE2 | INTERVENTIONAL | 01/08/2018 | mRNA drug |
| NCT03396575 | Brain Stem Gliomas Treated With Adoptive Cellular Therapy During Focal Radiotherapy Recovery Alone or With Dose-intensified Temozolomide (Phase I) | ACTIVE_NOT_RECRUITING | The standard of care for children with DIPG includes focal radiotherapy (RT) but outcomes have remained dismal despite this treatment. The addition of oral Temozolomide (TMZ) concurrently with RT followed by monthly TMZ was also found to be safe but ineffective. Recent studies in adults have shown that certain types of chemotherapy induce a profound but transient lymphopenia (low blood lymphocytes) and vaccinating and/or the adoptive transfer of tumor-specific lymphocytes into the cancer patient during this lymphopenic state leads to dramatic T cell expansion and potent immunologic and clinical responses. Therefore, patients in this study will either receive concurrent TMZ during RT and immunotherapy during and after maintenance cycles of dose-intensive TMZ (Group A) or focal radiotherapy alone and immunotherapy without maintenance DI TMZ (Group B). Immune responses during cycles of DC vaccination with or without DI TMZ will be evaluated in both treatment groups. | NO | Diffuse Intrinsic Pontine Glioma (DIPG)\|Brain Stem Glioma | BIOLOGICAL: TTRNA-DC vaccines with GM-CSF\|BIOLOGICAL: TTRNA-xALT\|DRUG: Cyclophosphamide + Fludarabine Lymphodepletive Conditioning\|DRUG: Dose-Intensified TMZ\|DRUG: Td vaccine\|BIOLOGICAL: Autologous Hematopoietic Stem Cells (HSC) | University of Florida | PHASE1 | INTERVENTIONAL | 17/07/2018 | Dendritic mRNA therapy |
| NCT03562832 | Investigation of Anti-tumour Effect and Tolerability of the PARP Inhibitor 2X-121 in Patients With Metastatic Breast Cancer Selected by the 2X-121 DRP | ACTIVE_NOT_RECRUITING | 2X-121 is a small molecule targeted inhibitor of Poly ADP ribose polymerase (PARP), a key enzyme involved in DNA damage repair in cancer cells. The PARP inhibitor demonstrated clinical activity in a prior Phase 1 study in a number of solid tumors. 2X-121 has a novel dual-inhibitory action against both PARP 1/2 and Tankyrase 1/2. The molecule is also active in P-glycoprotein expressing cells, suggesting it may overcome some of the PARP inhibitor resistance.  The Phase 2 study is using 2x-121 DRP庐 biomarker in metastatic breast cancer patients to identify patients likely to respond to and benefit from treatment with 2X-121. | NO | Metastatic Breast Cancer | DRUG: PARP inhibitor 2X-121 | Allarity Therapeutics | PHASE2 | INTERVENTIONAL | 20/06/2018 | mRNA drug |
| NCT03562832 | Investigation of Anti-tumour Effect and Tolerability of the PARP Inhibitor 2X-121 in Patients With Metastatic Breast Cancer Selected by the 2X-121 DRP | ACTIVE_NOT_RECRUITING | 2X-121 is a small molecule targeted inhibitor of Poly ADP ribose polymerase (PARP), a key enzyme involved in DNA damage repair in cancer cells. The PARP inhibitor demonstrated clinical activity in a prior Phase 1 study in a number of solid tumors. 2X-121 has a novel dual-inhibitory action against both PARP 1/2 and Tankyrase 1/2. The molecule is also active in P-glycoprotein expressing cells, suggesting it may overcome some of the PARP inhibitor resistance.  The Phase 2 study is using 2x-121 DRP庐 biomarker in metastatic breast cancer patients to identify patients likely to respond to and benefit from treatment with 2X-121. | NO | Metastatic Breast Cancer | DRUG: PARP inhibitor 2X-121 | Allarity Therapeutics | PHASE2 | INTERVENTIONAL | 20/06/2018 | mRNA drug |
| NCT04603612 | The Role of mRNA-based Urine Test (Xpert Bladder Cancer Monitor) in Predicting the Need for Second Look Biopsy After Transurethral Resection of Non Muscle-invasive Bladder Tumor. | COMPLETED | The Xpert BC Monitor is an mRNA-based urinary marker test for BC surveillance which measures the levels of five target mRNAs (ABL1, ANXA10, UPK1B, CRH and IGF2) from a voided urine sample by real-time RT-PCR. The Xpert BC Monitor automates and integrates sample processing, nucleic acid amplification, and the detection of target sequences.  The performance of Xpert BC monitor regarding its sensitivity and negative predictive value was shown to be considerably high reaching 84% and 93%, respectively. Moreover, this high sensitivity was maintained in low-grade (77%) and Ta tumors (82%). Xpert BC monitor is going to gain now a wide popularity among practitioners in surveillance of NMIBC patients due to its simplicity to do, reliability and reproducibility.  The investigators hypothesize that; Xpert BC monitor may be a useful tool in evaluation of patients, who are potentially candidates for repeat TURBT. It can refine the indications of repeat biopsy by exclusion of cases with negative test. | NO | Bladder Cancer | PROCEDURE: Primary TURBT | Mansoura University | NA | INTERVENTIONAL | 01/06/2018 | mRNA drug |
| NCT03480152 | Messenger RNA (mRNA)-Based, Personalized Cancer Vaccine Against Neoantigens Expressed by the Autologous Cancer | TERMINATED | Background:  Exome sequencing can identify certain gene mutations in a person's tumor. This can then be used to create cancer treatments. In this study, researchers will make a treatment called a messenger ribonucleic acid (mRNA) vaccine. The vaccine might cause certain tumors to shrink.  Objective:  To see if the mRNA vaccine is safe and can cause metastatic melanoma or epithelial tumors to shrink.  Eligibility:  People 18-70 years old with metastatic melanoma or epithelial cancer  Design:  Participants will be screened under protocol 99-C-0128.  Participants will provide samples under protocol 03-C-0277:  Participants will provide a piece of their tumor from a previous surgery or biopsy.  Participants will have leukapheresis: Blood is removed through a needle in one arm and circulated through a machine that takes out the white blood cells. The blood is then returned through a needle in the other arm.  Participants will have many tests:  Scans and x-rays  Heart and lung function tests  Blood and urine tests  Participants will receive the mRNA vaccine every 2 weeks for up to 8 weeks. They will get the vaccine as an injection into the upper arm or thigh. They may receive a second course of vaccines if the study doctor determines it is needed.  Participants will have follow-up visits approximately 2 weeks after their final vaccine, then 1 month later, then every 1-2 months for the first year, and then once a year for up to 5 years. Each visit may take up to 2 days and include:  Physical exam  Blood tests  Scans  Leukapheresis at the first visit | YES | Melanoma\|Colon Cancer\|Gastrointestinal Cancer\|Genitourinary Cancer\|Hepatocellular Cancer | BIOLOGICAL: National Cancer Institute (NCI)-4650, a messenger ribonucleic acid (mRNA)-based, Personalized Cancer Vaccine | National Cancer Institute (NCI) | PHASE1\|PHASE2 | INTERVENTIONAL | 18/05/2018 | neoantigen mRNA treatment |
| NCT03468244 | Clinical Study of Personalized mRNA Vaccine Encoding Neoantigen in Patients With Advanced Digestive System Neoplasms | UNKNOWN | A single arm, open-label pilot study is designed to determine the safety, tolerability and effectiveness of personalized mRNA tumor vaccine encoding neoantigen in Patients with advanced esophageal squamous carcinoma, gastric adenocarcinoma, pancreatic adenocarcinoma and colorectal adenocarcinoma | NO | Advanced Esophageal Squamous Carcinoma\|Gastric Adenocarcinoma\|Pancreatic Adenocarcinoma\|Colorectal Adenocarcinoma | BIOLOGICAL: Personalized mRNA Tumor Vaccine | Changhai Hospital | NA | INTERVENTIONAL | 01/05/2018 | neoantigen mRNA treatment |
| NCT03431311 | T Cell Receptor Based Therapy of Metastatic Colorectal Cancer | TERMINATED | T Cell Receptor Based Therapy of Metastatic Colorectal Cancer With mRNA-engineered T Cells Targeting Transforming Growth Factor Beta Receptor Type II (TGF尾II) | NO | Colorectal Cancer | BIOLOGICAL: Adoptive Cell Therapy (ACT) | Oslo University Hospital | PHASE1\|PHASE2 | INTERVENTIONAL | 08/03/2018 | mRNA drug |
| NCT03444987 | The Role of Fibroblast Activation in Uterine Fibroid | COMPLETED | Uterine fibroids (UFs), also called uterine leiomyomas or myomas, are steroid hormone-responsive, benign tumors of the smooth muscle compartment (myometrium) of the uterus. They are the most common neoplasm affecting women in their reproductive age. It is estimated that up to 77% of women develop UF in their life. UFs are one of the leading causes of hospitalisations for gynaecological disorders and are the most frequent reason for hysterectomy. According to relevant literature, 40%-60% of all the hysterectomies performed are due to the presence of UFs. | NO | Uterine Fibroid | GENETIC: Measurement of protein expression in tissues. | Assiut University | | OBSERVATIONAL | 01/03/2018 | mRNA drug |
| NCT04317261 | Role of Novel Urinary Genomic and Metablomic Markers in Diagnosis of Bladder Cancer in Patients With Hematuria | COMPLETED | Our goal is to develop a noninvasive, fast and simple assay to detect BCa on the GeneXpert system and metablomic genes. | NO | Hematuria\|Bladder Cancer | DIAGNOSTIC_TEST: Xpert Bladder Cancer Monitor (Xpert Monitor) test and assay of urinary metabolomics (gene expression of CRAT and SLC) | Mansoura University | NA | INTERVENTIONAL | 01/03/2018 | mRNA drug |
| NCT03164772 | Phase 1/2 Study of Combination Immunotherapy and Messenger Ribonucleic Acid (mRNA) Vaccine in Subjects With NSCLC | COMPLETED | This is an open-label, multicenter, 2-arm study to evaluate the safety and preliminary efficacy of the addition of a vaccine therapy to 1 or 2 checkpoint inhibitors for NSCLC.  Arm A: messenger ribonucleic acid (mRNA) Vaccine \[BI 1361849 (formerly CV9202)\] + anti-programmed death ligand 1 (PD-L1) antibody \[durvalumab\]  Arm B: messenger ribonucleic acid (mRNA) Vaccine \[BI 1361849\] + anti-programmed death ligand 1 (PD-L1) \[durvalumab\] + anti-cytotoxic T-lymphocyte-associated protein 4 (CTLA-4) antibody \[tremelimumab\]  The run-in evaluation phase is followed by an expansion phase in which the cohort is expanded to 20 subjects (inclusive of subjects from the run-in). | YES | Metastatic Non-small Cell Lung Cancer\|NSCLC | DRUG: Durvalumab\|DRUG: Tremelimumab\|BIOLOGICAL: BI 1361849\|DEVICE: PharmaJet Tropis device | Ludwig Institute for Cancer Research | PHASE1\|PHASE2 | INTERVENTIONAL | 20/12/2017 | mRNA drug |
| NCT03164772 | Phase 1/2 Study of Combination Immunotherapy and Messenger Ribonucleic Acid (mRNA) Vaccine in Subjects With NSCLC | COMPLETED | This is an open-label, multicenter, 2-arm study to evaluate the safety and preliminary efficacy of the addition of a vaccine therapy to 1 or 2 checkpoint inhibitors for NSCLC.  Arm A: messenger ribonucleic acid (mRNA) Vaccine \[BI 1361849 (formerly CV9202)\] + anti-programmed death ligand 1 (PD-L1) antibody \[durvalumab\]  Arm B: messenger ribonucleic acid (mRNA) Vaccine \[BI 1361849\] + anti-programmed death ligand 1 (PD-L1) \[durvalumab\] + anti-cytotoxic T-lymphocyte-associated protein 4 (CTLA-4) antibody \[tremelimumab\]  The run-in evaluation phase is followed by an expansion phase in which the cohort is expanded to 20 subjects (inclusive of subjects from the run-in). | YES | Metastatic Non-small Cell Lung Cancer\|NSCLC | DRUG: Durvalumab\|DRUG: Tremelimumab\|BIOLOGICAL: BI 1361849\|DEVICE: PharmaJet Tropis庐 device | Ludwig Institute for Cancer Research | PHASE1\|PHASE2 | INTERVENTIONAL | 20/12/2017 | mRNA drug |
| NCT03291002 | Study of Intratumoral CV8102 in cMEL, cSCC, hnSCC, and ACC | UNKNOWN | This study evaluates intratumoral administration of CV8102 in patients with advanced melanoma, squamous cell carcinoma of the skin, squamous cell carcinoma of the head and neck, or adenoid cystic carcinoma.  Patients will receive CV8102 as single agent or in combination with SoC anti-PD-1 therapy. | NO | Melanoma (Skin)\|Squamous Cell Carcinoma of the Skin\|Carcinoma, Squamous Cell of Head and Neck\|Carcinoma, Adenoid Cystic | BIOLOGICAL: CV8102\|BIOLOGICAL: CV8102 + anti-PD-1 therapy | CureVac | PHASE1 | INTERVENTIONAL | 25/09/2017 | mRNA drug |
| NCT03203005 | IMA970A Plus CV8102 in Very Early, Early and Intermediate Stage Hepatocellular Carcinoma Patients | COMPLETED | This study is being carried out in order to evaluate a new cancer vaccine called IMA970A combined with CV8102, a new adjuvant for the treatment of liver cancer (hepatocellular carcinoma). It will be investigated whether IMA970A and CV8102 is safe and can trigger an immune response against the tumor, which may prevent the tumor (cancer) from recurring or spreading or may even lead to tumor shrinkage following the standard treatments the patients have previously received. | NO | Hepatocellular Carcinoma | DRUG: IMA970A plus CV8102 and Cyclophosphamide | National Cancer Institute, Naples | PHASE1\|PHASE2 | INTERVENTIONAL | 18/09/2017 | mRNA drug |
| NCT03323398 | Dose Escalation and Efficacy Study of mRNA-2416 for Intratumoral Injection Alone and in Combination With Durvalumab for Participants With Advanced Malignancies | TERMINATED | This clinical study will assess the safety and tolerability of escalating doses of mRNA-2416 alone and in combination with administered fixed doses of durvalumab in participants with relapsed/refractory solid tumor malignancies or lymphoma, as well as the objective response rate (ORR) of mRNA-2416 alone or in combination with durvalumab in ovarian cancer based on Response Evaluation Criteria in Solid Tumors (RECIST) v1.1. The applicable dose of mRNA-2416 will be injected directly into the participant's tumor (intratumoral) and the applicable dose of durvalumab will be administered intravenously. | NO | Relapsed/Refractory Solid Tumor Malignancies or Lymphoma\|Ovarian Cancer | BIOLOGICAL: mRNA-2416\|BIOLOGICAL: Durvalumab | ModernaTX, Inc. | PHASE1\|PHASE2 | INTERVENTIONAL | 15/08/2017 | mRNA drug |
| NCT03313778 | Safety, Tolerability, and Immunogenicity of mRNA-4157 Alone and in Combination in Participants With Solid Tumors | RECRUITING | The purpose of this study is to assess the safety, tolerability, and immunogenicity of mRNA-4157 alone and in combination in participants with solid tumors. | NO | Solid Tumors | BIOLOGICAL: mRNA-4157\|BIOLOGICAL: Pembrolizumab\|BIOLOGICAL: SoC Treatment | ModernaTX, Inc. | PHASE1 | INTERVENTIONAL | 14/08/2017 | mRNA drug |
| NCT02649829 | Autologous Dendritic Cell Vaccination in Mesothelioma | ACTIVE_NOT_RECRUITING | In this multicenter phase I/II trial, dendritic cells (DCs) loaded with the mesothelioma-associated tumor antigen WT1 will be used in conjunction with conventional chemotherapy for the frontline treatment of malignant pleural mesothelioma (MPM). The general objective is to provide the first-in-human experimental demonstration that the combination of platinum/pemetrexed-based chemotherapy with WT1-targeted DC vaccination is feasible and safe and enables the induction of both systemic and in situ mesothelioma-specific immune responses in patients with MPM. | NO | Malignant Pleural Mesothelioma | BIOLOGICAL: dendritic cell vaccination plus chemotherapy | University Hospital, Antwerp | PHASE1\|PHASE2 | INTERVENTIONAL | 01/08/2017 | Dendritic mRNA therapy |
| NCT03394937 | Safety of Intranodal ECI-006 in Melanoma Patients | TERMINATED | The purpose of this study is to assess the safety and tolerability of cancer immunotherapy ECI-006 and to determine its ability to induce a measurable immune response against the tumor associated antigens.  In Cohort 1, ECI-006 will be administered 5 times by intranodal injection in melanoma patients after resection of their tumor.  In Cohort 2, ECI-006 will be administered 9 times by intranodal injection on top of standard of care anti PD1 in metastatic melanoma patients with stable disease after 3 to 12 months treatment.  ECI-006 activates key immunologically active cells to direct the immune system against the cancer. Expected potential risks for ECI-006 are non-serious and related to the local administration of the product. Hence, the therapy suggested here has the promise to offer considerable benefit to patients without any major risk. | NO | Melanoma | BIOLOGICAL: ECI-006 | eTheRNA immunotherapies | PHASE1 | INTERVENTIONAL | 27/06/2017 | mRNA drug |
| NCT02864836 | Study of Copper Isotope in Head and Neck Cancer | UNKNOWN | The distribution of stable (non-radioactive) isotopes in living organisms is increasingly studied, in particular the zinc (Zn), copper (Cu) and iron (Fe), not only in primitive organisms, but also in mammals.  The scientific community shows a growing interest in the study of the isotopic distribution of Cu in humans: this distribution can vary according to gender or nutrition. Concerning pathology, the isotopic distribution of Cu seems interesting in Wilson's disease or in cirrhosis.  Additionally, a promising area of study focuses on the role of Cu in cancerous tumors, neoangiogenesis, the mechanisms of free radicals reduction and signaling pathways.  Head and neck cancers are sensitive to platinum salts. Links between platinum and Cu are important: platinum penetrates into the cell through a Cu receptor, it interacts with the regulation mechanisms of Cu and platinum.  Preliminary studies suggest a variation of the measurable isotopic distribution of Zn in patients with breast tumor and of Cu in patients presenting breast as well as colorectal tumors.  The Larner et al. study suggest a promising role of Zn in breast cancer, indeed, results highlight a variation of distribution of Zn in 10 breast tumors. Concerning the study of T茅louk et al. on 8 patients presenting colorectal tumors and 20 patients presenting breast tumors, results are in favor of an increase of mortality when Cu 65 is decreased in the serum and the isotopic modifications happen earlier than usual modifications of biochemical tumor markers such as: carbohydrate antigen (CA) 19.9, Carcinoma Antigen (CA) 15.3, Carcinoembryonic antigen (CEA).  Currently, there is no information about the distribution of the stable isotopes of Cu in head and neck tumors.  The objective of the study is to determine if the distribution of 65Cu / 63Cu is modified in tumoral tissues compared to healthy tissues. The isotopic distribution of the Cu in 2 tumor types, head and neck tumors and lymphomas, will be also investigated in order to determine if this distribution is specific of a tumor type or not.  In case of positivity of this variation, the prognostic interest of these parameters will be evaluated. | NO | Head and Neck Tumors | PROCEDURE: Samples collection | Lille Catholic University | | OBSERVATIONAL | 15/05/2017 | mRNA drug |
| NCT02872025 | Immunotherapy in High-risk Ductal Carcinoma in Situ (DCIS) | RECRUITING | This is a study to investigate the change in the immune microenvironment of high risk ductal carcinoma in situ (DCIS) after short term exposure to immunotherapy. | NO | Carcinoma, Intraductal, Noninfiltrating | DRUG: Pembrolizumab\|BIOLOGICAL: Intralesional mRNA 2752 | Laura Esserman | EARLY_PHASE1 | INTERVENTIONAL | 12/12/2016 | mRNA drug |
| NCT02465268 | Vaccine Therapy for the Treatment of Newly Diagnosed Glioblastoma Multiforme | COMPLETED | The purpose of this research study is to determine if an investigational dendritic cell vaccine, called pp65 DC, is effective for the treatment of a specific type of brain tumor called glioblastoma (GBM) when given with stronger doses of routine chemotherapy. | NO | Glioblastoma Multiforme\|Glioblastoma\|Malignant Glioma\|Astrocytoma, Grade IV\|GBM | BIOLOGICAL: pp65-shLAMP DC with GM-CSF\|BIOLOGICAL: unpulsed PBMC and saline\|DRUG: Td\|DRUG: Saline\|BIOLOGICAL: pp65-flLAMP DC with GM-CSF | University of Florida | PHASE2 | INTERVENTIONAL | 09/08/2016 | Dendritic mRNA therapy |
| NCT03083054 | Cellular Immunotherapy for Patients With High Risk Myelodysplastic Syndromes and Acute Myeloid Leukemia | UNKNOWN | The main objective of this work is to conduct a clinical study for the development and application of a vaccine with autologous dendritic cells submitted to electroporation with Wilm's tumor 1 (WT1) messenger ribonucleic acid (mRNA), as an adjuvant treatment of high-risk Myelodysplastic Syndromes and Acute Myeloid Leukemia, aiming to delay the progression of the disease or its relapse and increase overall and event-free survival. | NO | Myelodysplastic Syndromes\|Acute Myeloid Leukemia | BIOLOGICAL: Autologous dendritic cells electroporated with WT1 mRNA | University of Campinas, Brazil | PHASE1\|PHASE2 | INTERVENTIONAL | 01/08/2016 | Dendritic mRNA therapy |
| NCT02700971 | RATIO: Rational Approach To Immuno-Oncology | UNKNOWN | This trial will utilize a Molecular MicroscopeTM diagnostic system (MMDxTM) that combines the molecular and histopathological features of biopsies, plus clinical and laboratory parameters, to create the first Integrated Diagnostic System. This MMDxTM will be utilized to phenotype cutaneous melanoma biopsy samples to detect an immune responsive mRNA signature. | NO | Metastatic Melanoma | PROCEDURE: Molecular Microscope Diagnostic system | AHS Cancer Control Alberta | NA | INTERVENTIONAL | 23/06/2016 | mRNA drug |
| NCT02564614 | A Study of Hypoxia-inducible Factor 1a (HIF1A) Messenger Ribonucleic Acid (mRNA) Antagonist (RO7070179), to Demonstrate Proof-of-mechanism in Adult Participants With Hepatocellular Carcinoma (HCC) | COMPLETED | This open-label study will demonstrate proof-of-mechanism of HIF1A inhibition by a decrease of HIF1A mRNA after intravenous (IV) infusion of RO7070179 in participants with hepatocellular carcinoma (HCC) who have failed at least one line of systemic therapy. This will be a single arm study and all participants will receive RO7070179, 13 milligram per kilogram per week (mg/kg/week), 2-hour IV infusion on Days 1 and 4 during Week 1 of Cycle 1, followed by once weekly in 6 week cycle. Treatment with RO7070179 will be continued until disease progression or unacceptable toxicity. | NO | Carcinoma, Hepatocellular | DRUG: RO7070179 | Hoffmann-La Roche | PHASE1 | INTERVENTIONAL | 02/05/2016 | mRNA drug |
| NCT02808364 | Personalized Cellular Vaccine for Recurrent Glioblastoma (PERCELLVAC2) | COMPLETED | The treatment option for recurrent glioblastoma is limited. Immune cell based therapy for glioblastoma has shown some efficacy. This study is designed to perform a personalized clinical trial by first analyzing the expression of tumor associated antigens in patients with recurrent glioblastoma and then immunizing the patients with personalized antigen pulsed DCs. Immune responses to the immunized antigens will be monitored. Safety and efficacy will be observed in this study. | NO | Glioblastoma | BIOLOGICAL: Personalized cellular vaccine | Guangdong 999 Brain Hospital | PHASE1 | INTERVENTIONAL | 01/03/2016 | mRNA drug |
| NCT02808416 | Personalized Cellular Vaccine for Brain Metastases (PERCELLVAC3) | COMPLETED | Cancer patients with brain metastases (BM) have poor prognosis. Current treatments produce limited efficacy. Recent advance in cancer immunotherapy has provided important new means to treat cancer patients at advanced stages. This study is designed to perform a clinical trial to treat advanced caner patients with brain metastases with personalized dendritic cell-based cellular vaccines. The patients will receive vaccines consisting of mRNA tumor antigen pulsed DCs. Immune response to the immunized tumor antigens will be monitored. Safety and efficacy will be observed in this study. | NO | Brain Cancer\|Neoplasm Metastases | BIOLOGICAL: Personalized cellular vaccine | Guangdong 999 Brain Hospital | PHASE1 | INTERVENTIONAL | 01/03/2016 | mRNA drug |
| NCT02528682 | MiHA-loaded PD-L-silenced DC Vaccination After Allogeneic SCT | COMPLETED | Allogeneic stem cell transplantation (allo-SCT) is a potent treatment, and sometimes the only curative treatment for aggressive hematological malignancies. The therapeutic efficacy is attributed to the graft-versus-tumor (GVT) response, during which donor-derived CD8+ T cells become activated by recipient minor histocompatibility antigens (MiHA) presented on dendritic cells (DC). Consequently, these alloreactive donor T cells clonally expand, acquire effector functions and kill MiHA-positive malignant cells. However, in a substantial number of patients persistence and recurrence of malignant disease is observed, indicating that insufficient GVT immunity is induced. This is reflected by our observation that not all patients develop a productive CD8+ T cell response towards MiHA mismatched between the recipient and donor. We found that the PD-1/PD-L1 co-inhibitory pathway is involved in dampening MiHA-specific CD8+ T cell expansion and function post-transplantation. Therefore, a promising strategy to induce or boost GVT immune responses is pre-emptive or therapeutic vaccination with ex vivo-generated donor DCs loaded with MiHA that are exclusively expressed by recipient hematopoietic cells and their malignant counterparts. In contrast to pre-emptive donor lymphocyte infusion (DLI) with polyclonal donor T cells, this MiHA-DC vaccination approach has less risk of inducing graft-versus-host disease (GVHD) and the potency to induce more efficient GVT-associated T cell immunity. In addition, the potency of this DC vaccine will be further enhanced by interference with the PD-1/PD-L1 co-inhibitory pathway, using siRNA mediated PD-L1/PD-L2 silencing. | NO | Hematological Malignancies | BIOLOGICAL: MiHA-loaded PD-L-silenced DC Vaccination | Radboud University Medical Center | PHASE1\|PHASE2 | INTERVENTIONAL | 01/01/2016 | Dendritic mRNA therapy |
| NCT02529072 | Nivolumab With DC Vaccines for Recurrent Brain Tumors | COMPLETED | Patients will be randomized to one of two treatment arms - Group I and Group II. Group I will receive nivolumab monotherapy until surgical resection, and Group II will receive nivolumab alone and with DC vaccine therapy until surgical resection. During surgical resection blood and tumor samples will be assessed and compared. Following surgery, both groups will continue to receive DC vaccines (total of 8) and nivolumab therapy until confirmed progression. | YES | Malignant Glioma\|Astrocytoma\|Glioblastoma | DRUG: nivolumab\|BIOLOGICAL: DC | Gary Archer Ph.D. | PHASE1 | INTERVENTIONAL | 01/01/2016 | Dendritic mRNA therapy |
| NCT02649582 | Adjuvant Dendritic Cell-immunotherapy Plus Temozolomide in Glioblastoma Patients | RECRUITING | In this phase I/II trial, the primary objective is to determine overall and progression-free survival of patients with newly diagnosed glioblastoma when autologous Wilms' tumor 1 (WT1) messenger (m)RNA-loaded dendritic cell (DC) vaccination is added to adjuvant temozolomide maintenance treatment following (sub)total resection and temozolomide-based chemoradiation. | NO | Glioblastoma Multiforme of Brain | BIOLOGICAL: Dendritic cell vaccine plus temozolomide chemotherapy | University Hospital, Antwerp | PHASE1\|PHASE2 | INTERVENTIONAL | 01/12/2015 | Dendritic mRNA therapy |
| NCT02366728 | DC Migration Study for Newly-Diagnosed GBM | COMPLETED | This randomized phase II study will assess the impact of pre-conditioning on migration and survival among newly diagnosed glioblastoma (GBM) patients who have undergone definitive resection and completed standard temozolomide (TMZ) and radiation treatment, as well as the impact of tetanus pre-conditioning and basiliximab together on survival. After completing standard of care radiotherapy with concurrent TMZ, patients will be randomized to 1 of 3 treatment arms: 1). receive cytomegalovirus (CMV)-specific dendritic cell (DC) vaccines with unpulsed (not loaded) DC pre-conditioning prior to the 4th vaccine; 2). receive CMV-specific DC vaccines with Tetanus-Diphtheria Toxoid (Td) pre-conditioning prior to the 4th vaccine; 3). receive basiliximab infusions prior to the 1st and 2nd DC vaccines along with Td pre-conditioning prior to the 4th vaccine. A permuted block randomization algorithm using a 1:1:1 allocation ratio will be used to assign patients to a treatment arm. Randomization will be stratified by CMV status (positive, negative), with the assignment to arms I and II being double-blinded. Effective March 2017, randomization to Group III has been terminated. | YES | Glioblastoma\|Astrocytoma, Grade IV\|Giant Cell Glioblastoma\|Glioblastoma Multiforme | BIOLOGICAL: Unpulsed DCs\|BIOLOGICAL: Td\|BIOLOGICAL: Human CMV pp65-LAMP mRNA-pulsed autologous DCs\|BIOLOGICAL: 111In-labeled DCs\|DRUG: Temozolomide\|DRUG: Saline\|DRUG: Basiliximab | Mustafa Khasraw, MBChB, MD, FRCP, FRACP | PHASE2 | INTERVENTIONAL | 12/10/2015 | Dendritic mRNA therapy |
| NCT02692976 | Natural Dendritic Cells for Immunotherapy of Chemo-naive Metastatic Castration-resistant Prostate Cancer Patients | COMPLETED | Prostate cancer is the only type of cancer in which conventional dendritic cells (DC) treatment has a beneficial effect on the overall survival. In this study investigators aim to show immunologic efficacy of tumor-peptide loaded natural DC in metastatic castration-resistant prostate cancer patients (mCRPC).  The immunomonitoring will include:  1. functional response and tetramer analysis of delayed-type hypersensitivity infiltrating lymphocytes against tumor peptides and 2. type I interferon (IFN) gene expression in peripheral blood mononuclear cells, and 3. proliferative, effector cytokine- and humoral responses to keyhole limpet hemocyanin, a immunogenic protein providing T cell help.  The secondary objectives are the safety and feasibility of natural DC vaccinations, the influence on the quality of life during treatment with natural DC, and the clinical efficacy of treatment. | NO | Prostatic Neoplasms\|Immunotherapy\|Dendritic Cells\|Vaccines | BIOLOGICAL: mDC vaccination\|BIOLOGICAL: pDC vaccination\|BIOLOGICAL: mDC and pDC vaccination | Radboud University Medical Center | PHASE2 | INTERVENTIONAL | 01/09/2015 | Dendritic mRNA therapy |
| NCT02438007 | A Study of Galeterone Compared to Enzalutamide In Men Expressing Androgen Receptor Splice Variant-7 mRNA (AR-V7) Metastatic CRPC | TERMINATED | The purpose of this study is to compare galeterone to enzalutamide in men expressing androgen receptor spice variant-7 mRNA (AR-V7) in metastatic (M1) castrate resistant prostate cancer (CRPC). | NO | Prostate Cancer | DRUG: Galeterone\|DRUG: Enzalutamide | LTN PHARMACEUTICALS, INC. | PHASE3 | INTERVENTIONAL | 01/06/2015 | mRNA drug |
| NCT03357991 | E6/E7 mRNA Performance to Detect HSIL and Cost-effectiveness Analysis of This Screening Strategy in HIV + MSM | UNKNOWN | This study evaluates the positive and negative predictive value of E6/E7 mRNA expression for anal HSIL and its capacity to predict incident HSIL in HIV + MSM. We also analyse the cost-effectiveness of this new screening strategy. It is an ambispective study with 355 participants and a follow-up period of 2 to 5 years. | NO | HPV - Anogenital Human Papilloma Virus Infection\|HSIL, High Grade Squamous Intraepithelial Lesions\|Anal Cancer\|Hiv | | Hospital Universitari de Bellvitge | | OBSERVATIONAL | 11/01/2015 | mRNA drug |
| NCT02294578 | Salivary Biomarkers for Non-small Cell Lung Cancer Detection | UNKNOWN | The investigators plan to recruit patients for a prospective study in patients in need of evaluation for lung lesions suspicious for cancer. Saliva samples will be collected before diagnostic evaluation including biopsy with subsequent blinded examination of the salivary markers without knowledge of the disease status. This prospective recruitment with retrospective blinded evaluation or PRoBE design satisfies the highest standards recommended by the National Cancer Institute for biomarker development. This process limits the selection bias that can confound retrospective studies. As the primary endpoint, a pre-specified multi-marker panel will be evaluated based on the combination of sensitivity and specificity. In addition, seven pre-specified individual candidate mRNA cancer markers and six internal reference or "housekeeping" genes will be evaluated. The performance of new multi-marker panels will also be assessed and compared with the prior pre-specified model based on sensitivity and specificity combinations as well as the area under the receiver operating characteristic curve. | NO | Lung Cancer | | PeriRx |  | OBSERVATIONAL | 01/12/2014 | mRNA drug |
| NCT02423590 | Study of Gemcitabine/Carboplatin First-line Chemotherapy +/- Apatorsen in Advanced Squamous Cell Lung Cancers | UNKNOWN | This study is being carried out to see if a new drug called Apatorsen in combination with standard gemcitabine/carboplatin chemotherapy is effective in treating squamous cell lung cancer.  This study is part of a research project for collecting information about the effectiveness and safety of Apatorsen when used with gemcitabine/carboplatin chemotherapy. The main purpose of this study is to see if Apatorsen, when combined with gemcitabine/carboplatin, is an effective treatment for squamous cell lung cancer.  Recent research has found that a protein called Hsp27 can help cancer cells protect themselves against the effects of cancer treatments. Hsp27 is only found in some lung cancers but when it is present, cancer drugs might not work as well as they would without Hsp27 being present. Blocking the action of Hsp27 or removing Hsp27 from cancer cells with Apatorsen may slow down or stop the cancer growing. This study will therefore look at the relationship between the Hsp27 levels in tumour and blood and the effect of the treatment.  The development of Apatorsen is intended to provide a new treatment option for patients with cancer. Apatorsen may also make the cancer more sensitive to gemcitabine and carboplatin and so make this chemotherapy treatment more effective. | NO | Squamous Cell Lung Cancer | DRUG: Apatorsen (OGX-427)\|DRUG: Gemcitabine\|DRUG: Carboplatin | Queen Mary University of London | PHASE2 | INTERVENTIONAL | 01/06/2014 | mRNA drug |
| NCT02140138 | An Open Label Randomised Trial of RNActive庐 Cancer Vaccine in High Risk and Intermediate Risk Patients With Prostate Cancer | TERMINATED | The purpose of this study is to evaluate the induction of immune responses against CV9104 administered by conventional intradermal injection or with a needle-free intradermal injection device and to assess the safety and tolerability of CV9104 administered by conventional intradermal injection versus injection with a needle-free intradermal injection device versus no injection. | NO | Prostate Carcinoma | BIOLOGICAL: CV9104\|DEVICE: needle free injection device (Tropis庐) | CureVac | PHASE2 | INTERVENTIONAL | 01/06/2014 | mRNA drug |
| NCT01995708 | CT7, MAGE-A3, and WT1 mRNA-electroporated Autologous Langerhans-type Dendritic Cells as Consolidation for Multiple Myeloma Patients Undergoing Autologous Stem Cell Transplantation | COMPLETED | The purpose of this study is to see if the investigator can help the immune system to work against myeloma.  This study will see if a vaccine made with altered dendritic cells will make T cells work against tumor cells. The stem cells collected for the transplant will also be used to grow dendritic cells in the lab. The dendritic cells will carry the antigens. These cells then will be injected under the skin. The investigators will do lab studies before and after the vaccination to find out if the vaccine is working. | YES | Multiple Myeloma | BIOLOGICAL: CT7, MAGE-A3, and WT1 mRNA-electroporated Langerhans cells ( LCs)\|OTHER: Standard of care | Memorial Sloan Kettering Cancer Center | PHASE1 | INTERVENTIONAL | 31/01/2014 | mRNA drug |
| NCT01734304 | DC Vaccination for Postremission Therapy in AML | COMPLETED | The aim of this study is to determine the feasibility and safety of an autologous DC immunotherapy in patients with AML of non-favorable risk profile. | NO | Acute Myeloid Leukemia | BIOLOGICAL: DC vaccination for postremission therapy in AML | Ludwig-Maximilians - University of Munich | PHASE1\|PHASE2 | INTERVENTIONAL | 05/11/2013 | Dendritic mRNA therapy |
| NCT01837693 | Cervical Cancer Prevention: From DNA to mRNA? - New Technologies for Cervical Cancer Screening 2 | UNKNOWN | In industrialized countries, cervical cancer is a well controlled disease thanks to the diffusion of Pap test and, in particular, to organized screening programs, which are able to detect and treat pre-invasive lesions (cervical intraepithelial neoplasia, CIN). The human papilloma virus (HPV) has been recognised as the necessary, but not sufficient, cause of cervical cancer, so a new screening test based on the identification of high risk (HR) HPV types has been developed(HPV DNA test). This test has demonstrated to be more effective than cytology in reducing the incidence and the mortality of cervical cancer, but it is less specific, so the use of a test triage is necessary to reduce the number of colposcopies and the risk of over-diagnosis (due to the potential regressivity of pre-invasive lesions). Until now, the triage test used is the cytology (Pap test).  Recently specific biomarkers (mRNA and p16 tests) have been introduced for high grade CIN, targeting the molecular alterations strictly associated to transformation rather than simply detecting HR-HPV infections. These tests are more specific than HPV DNA test with a modest reduction of sensitivity for high-grade lesions.  This is a multicenter randomised trial nested into some Italian screening programs based on the use of HPV DNA test as primary test.  All women with positive HPV DNA test will be tested for cytology and also for mRNA and p16. Women with positive cytology will be referred to colposcopy, while women with negative cytology will be randomized into two arms.  This study aims to evaluate if mRNA and p16 could be used as test of triage of HPV DNA or as a primary screening test with direct sending in colposcopy.  In particular the main objectives are:  * Measuring the cumulative detection rate of CIN2+ in the five years following a HPV DNA positive test and mRNA or p16 negative. * Measuring the potential reduction of overdiagnosis of using mRNA or p16 test instead of DNA, with direct sending in colposcopy * Measuring the reduction of overdiagnosis of cytological triage or triage with mRNA or p16 compared to the direct sending in colposcopy in women with HPV DNA test positive.  Secondary objectives are:  * to assess the feasibility of mRNA testing in primary screening * to validate the sample techniques for the new tests * to standardize quality controls for the the new tests | NO | Precancerous Conditions\|Neoplasms | PROCEDURE: Experimental: immediate colposcopy | Azienda Unit脿 Sanitaria Locale Reggio Emilia | NA | INTERVENTIONAL | 01/06/2013 | mRNA drug |
| NCT02116920 | HPV E6/E7 mRNA Versus HPV DNA as Triage for Cervical Cancer Screening | UNKNOWN | AIM: To develop and standardize a cost effective methodology or algorithm for mRNA E6/E7 for HPV genotypes 16, 18, 31, 33 and 45 as compared to commercially available assays which can be incorporated to triage excess false positives from primary screening for cervical cancers  Objectives:  1. Development and standardization of methodology /algorithm for mRNA E6/E7 testing for HPV genotypes 16, 18, 31, 33 and 45 using Real-time RT-PCR, in cervical samples. 2. To compare the test performance of this HPV E6/E7 mRNA assay to HPV DNA by HC 2 as secondary screening test, with the reference standard of colposcopy with biopsy, to triage women found positive in primary screening by VIA , in a population based screening for cancer of cervix. 3. To determine number of false positives in the primary screening test after testing VIA positives with a known high specificity secondary screening test (HPV-DNA HC II ) compared to HPV E6/E7 mRNA testing.  Study Population: Women in the age group of 30-65 years, who test positive on primary cervical screening test VIA will be enrolled for the proposed diagnostic tests along with reference standard of colposcopy with guided biopsy.  Methodology:  Women in the age group of 30-65 years undergoing routine cervical cancer screening through hospital ( Preventive Oncology screening clinic) and community based screening programs with abnormal test result using the primary cervical cancer screening test VIA will be recruited in the study. The primary screening test VIA will be administered by application of 5% Acetic Acid to the cervix and visualizing the cervix with the help of a halogen focus lamp. VIA will be considered to be positive if definite acetowhite lesions are visualized close to the squamocolumnar junction. | NO | CIN\|Cervix Cancer | | Tata Memorial Hospital | | OBSERVATIONAL | 01/05/2013 | mRNA drug |
| NCT01676779 | mRNA Electroporated Autologous Dendritic Cells for Stage III/IV Melanoma | COMPLETED | This is an open label, 2-arm, 1-stage, randomized controlled phase II study in patients with AJCC stage IIIB/C \& -IV melanoma. At baseline tumor assessment (using total body FDG-PET/CT), patients should be free from measurable tumor lesions (according to RECISTv1.1 definitions) following prior local therapy (e.g. following surgical resection, isolated limb perfusion, radiofrequency ablation, cryotherapy, radiotherapy, electrochemotherapy, ...). Patients should not have symptomatic non-measurable tumor lesions (e.g. bone metastasis, or pleural effusion), and lesions treated by prior local therapy should be free from progression. Patients should not have received any prior systemic therapy (non-experimental or experimental). | NO | Malignant Melanoma Stage III\|Malignant Melanoma Stage IV | BIOLOGICAL: Dendritic cell therapy | Universitair Ziekenhuis Brussel | PHASE2 | INTERVENTIONAL | 01/10/2012 | Dendritic mRNA therapy |
| NCT01686334 | Efficacy Study of Dendritic Cell Vaccination in Patients With Acute Myeloid Leukemia in Remission | ACTIVE_NOT_RECRUITING | The primary aim of this innovative immunotherapeutic study is to determine whether the antileukemic effects seen in our previous phase I/II study can be confirmed in a large cohort of patients and whether dendritic cell vaccination can significantly prevent relapse and increase survival of acute myeloid leukemia (AML) patients by eradicating minimal residual disease. | NO | Acute Myeloid Leukemia | BIOLOGICAL: DC vaccine | Zwi Berneman | PHASE2 | INTERVENTIONAL | 01/10/2012 | Dendritic mRNA therapy |
| NCT01456104 | Immune Responses to Autologous Langerhans-type Dendritic Cells Electroporated With mRNA Encoding a Tumor-associated Antigen in Patients With Malignancy: A Single-arm Phase I Trial in Melanoma | COMPLETED | This study is being done to see if the investigators can help the immune system to work against melanoma.A dendritic cell is another type of white blood cell. It has most, if not all, of the proteins needed to make T cells work to destroy cancer cells. However, dendritic cells do not normally have the cancer proteins on their surface. The challenge then is to combine the antigens with dendritic cells to make a vaccine. The investigators think that the body's T cells might then react against the tumor and help destroy it.This study will see if altered dendritic cells will make T cells work against tumor cells. The dendritic cells will be made in a lab and will carry the antigens. These cells then will be injected under the skin.In this study, the investigators are trying to help the body make a stronger immune response against the cancer. The patient will get the same kind of dendritic cell vaccine used in the earlier study, but with one major difference. The dendritic cells will contain messenger-RNA (mRNA). Cells use mRNA to make proteins. The mRNA will be put into dendritic cells by a laboratory method called electroporation. The mRNA is never given to the patient directly. This mRNA will help the dendritic cell make a tumor antigen like what the cancer expresses. The dendritic cell can then put this tumor antigen on its surface so that the body could make a stronger immune response against the tumor. | NO | Melanoma | BIOLOGICAL: Langerhans-type dendritic cells (a.k.a. Langerhans cells or LCs) | Memorial Sloan Kettering Cancer Center | PHASE1 | INTERVENTIONAL | 17/10/2011 | Dendritic mRNA therapy |
| NCT01446731 | Dendritic Cell Vaccination and Docetaxel for Patients With Prostate Cancer | COMPLETED | This is a randomized phase II trial including 40 patients with castration resistant metastatic cancer prostate (CRMPC).  Patients will be randomized between treatment with a dendritic cell vaccine plus docetaxel and docetaxel alone.  The primary objective is to evaluate the vaccine specific immune response and patients will be evlauated with blood tests and DTH reactions during the treatment course.  Secondary objectives are to evaluate clinical response by objective response (RECIST-criteria, 18F-NaF-PET/CT scan), PSA response, pain response and finally we determine time to progression and overall survival. | NO | Prostatic Neoplasms | BIOLOGICAL: mRNA transfected dendritic cell\|DRUG: Docetaxel | Inge Marie Svane | PHASE2 | INTERVENTIONAL | 01/10/2011 | Dendritic mRNA therapy |
| NCT01334047 | Trial of Vaccine Therapy in Recurrent Platinum Sensitive Ovarian Cancer Patients | TERMINATED | In this study the investigators will include patients with relapsed epithelial ovarian cancer. In spite of increased rates of complete response to initial chemotherapy, most patients with advanced ovarian cancer relapse and succumb to progressive disease. Immunotherapy may have potential for consolidation therapy. Dendritic cell vaccine is well toleranted in previous studies, with minor side effects and no serious adverse events registrated In this study, patients will receive DC-vaccine therapy after response to platinum treatment at relapse. The investigtors include patients in good clinical condition with no severe symptoms of the disease. If patients relapse during vaccine treatment, they will be discontinued from the study.  The investigators have included hTERT- and survivin mRNA in addition to amplified cancer stem cell mRNA in the vaccine. | NO | Recurrent Epithelial Ovarian Cancer | BIOLOGICAL: DC-006 vaccine | Steinar Aamdal | PHASE1\|PHASE2 | INTERVENTIONAL | 01/04/2011 | Dendritic mRNA therapy |
| NCT01337518 | A Phase 1a/1b Study to Evaluate the Safety of EZN-4176, in Adult Patients With Castration-Resistant Prostate Cancer | SUSPENDED | This study will evaluate an experimental drug called EZN-4176 to determine the anticancer effects when it is given to patients with an advanced form of prostate cancer called castration-resistant prostate cancer (CRPC). Goals of this phase I study include finding out the dose of EZN-4176 that can be safely given without serious side effects and to determine the amount of EZN-4176 that should be given in future studies. | NO | Prostatic Neoplasm | DRUG: EZN-4176 | Enzon Pharmaceuticals, Inc. | PHASE1 | INTERVENTIONAL | 01/03/2011 | mRNA drug |
| NCT02285413 | Platin-based Chemotherapeutics to Enhance Dendritic Cell Vaccine Efficacy in Melanoma Patients | COMPLETED | This is an exploratory study and the primary objective is the immunogenicity and feasibility of combined chemotherapy-DC vaccination. The secondary objectives are the toxicity and clinical efficacy. This study will provide important data on the immunological efficacy of DC immunochemotherapy. | NO | Melanoma | BIOLOGICAL: DC vaccination\|BIOLOGICAL: DC vaccination with cisplatinum | Radboud University Medical Center | PHASE2 | INTERVENTIONAL | 01/02/2011 | Dendritic mRNA therapy |
| NCT01197625 | Vaccine Therapy in Curative Resected Prostate Cancer Patients | ACTIVE_NOT_RECRUITING | In this study the investigators will include patients with high risk of PSA relapse scheduled to receive curative surgical treatment. This include patients with high Gleason score (9-10) or micrometastatic disease (tumor cells detected in specimens obtained from bone marrow). They are scheduled for regular follow-ups with PSA measurements. We have previously published that some patients with metastatic prostate cancer may respond to DC-vaccination with tumor mRNA, with a decrease in PSA. PSA response is related to immunological response. Patients receiving DC-vaccination may have a reduced risk of PSA relapse or increased time to PSA relapse. Previous experience with different DC-vaccine protocols in our hospital has resulted in only minor side-effects (grade 1-2 fever, rubor, fatigue, local swelling or pain). | NO | Prostate Cancer | BIOLOGICAL: Dendritic cell vaccine | Oslo University Hospital | PHASE1\|PHASE2 | INTERVENTIONAL | 01/09/2010 | Dendritic mRNA therapy |
| NCT01186328 | EZN-3042 Administered With Re-induction Chemotherapy in Children With Relapsed Acute Lymphoblastic Leukemia (ALL) | TERMINATED | An experimental drug called EZN-3042 targets survivin, a protein expressed in leukemia cells at relapse that promotes the leukemia cells to grow. The main goal of this phase I study is to find out the dose of EZN-3042 that can be safely given without serious side effects both alone and in combination with standard chemotherapy drugs during re-induction. | YES | Lymphoblastic Leukemia, Acute\|Lymphoblastic Leukemia, Acute, Childhood\|Leukemia, Lymphoblastic, Acute, T Cell\|Leukemia, Lymphoblastic, Acute | DRUG: EZN-3042\|DRUG: Cytarabine\|DRUG: Doxorubicin\|DRUG: Prednisone\|DRUG: Vincristine\|DRUG: PEG-asparaginase\|DRUG: Methotrexate\|DRUG: Hydrocortisone | Therapeutic Advances in Childhood Leukemia Consortium | PHASE1 | INTERVENTIONAL | 24/08/2010 | mRNA drug |
| NCT01291420 | Dendritic Cell Vaccination for Patients With Solid Tumors | COMPLETED | The aim of this study is to evaluate the immunogenicity and clinical efficacy of intradermal vaccination with autologous RNA-modified dendritic cells (DCs) - engineered to express the WT1 protein - in patients with limited spread metastatic solid tumors, i.e. breast cancers, glioblastoma grade IV, sarcomas, malignant mesothelioma and colorectal tumors. Based on the results of our previously performed phase I study with autologous WT1 mRNA-transfected DC, the investigators hypothesize that the vaccination with DC will be well-tolerated and will result in an increase in WT1-specific CD8+ T cell responses. | NO | Glioblastoma\|Renal Cell Carcinoma\|Sarcomas\|Breast Cancers\|Malignant Mesothelioma\|Colorectal Tumors | BIOLOGICAL: autologous dendritic cell vaccination | University Hospital, Antwerp | PHASE1\|PHASE2 | INTERVENTIONAL | 03/05/2010 | Dendritic mRNA therapy |
| NCT01530698 | Single-step Antigen Loading and TLR Activation of Dendritic Cells in Melanoma Patients | COMPLETED | Objectives: This is an exploratory study, consisting of two parts. In part I dose escalation is performed and the primary objective is the safety of different doses of TLR-DC and Trimix DC. In part II Trimix DC vaccination will be compared with TLR-DC vaccination and the primary objective of this part is the immunological response, with toxicity and clinical efficacy being secondary objectives. These studies will provide important data on the safety and immunological effects of TLR-DC and Trimix DC.  Study design: Part I of this study is an open label dose escalation study. Part II of this study is an open label randomized phase II study.  Study population: Our study population consists of melanoma patients, with proven expression of melanoma associated tumor antigens gp100 and tyrosinase. Melanoma patients with regional lymph node metastasis in whom a radical lymph node dissection is performed within 2 months of inclusion in this study (further referred to as stage III) and melanoma patients with measurable distant metastases (further referred to as stage IV) will be included. | NO | Melanoma | BIOLOGICAL: autologous dendritic cell vaccine\|BIOLOGICAL: autologous dendritic cell vaccine | Radboud University Medical Center | PHASE1\|PHASE2 | INTERVENTIONAL | 01/04/2010 | Dendritic mRNA therapy |
| NCT00965224 | Efficacy of Dendritic Cell Therapy for Myeloid Leukemia and Myeloma | UNKNOWN | Dendritic cell therapy is a promising strategy for adjuvant cancer therapy in the setting of minimal residual disease (MRD) to fight off cancer relapse and/or progression. The investigators already performed a phase I safety study in leukemia patients that were in complete remission demonstrating the absence of side effects and feasibility of the therapy. Here, the investigators want to extend on this strategy by studying the clinical efficacy of autologous DC vaccination in patients with acute and chronic myeloid leukemia and myeloma patients. Effects of DC therapy on the immune reactivity towards leukemia cells as well as clinical parameters such molecular MRD monitoring, time to relapse (TTR), progression-free survival (PFS) and overall survival(OS) will be studied in vaccinated and non-vaccinated (control) patients. Patients will be vaccinated using their own dendritic cells electroporated with mRNA coding for the full-length Wilms' tumor antigen WT1. | NO | Acute Myeloid Leukemia\|Chronic Myeloid Leukemia\|Multiple Myeloma | BIOLOGICAL: dendritic cell vaccination (active specific immunotherapy) | University Hospital, Antwerp | PHASE2 | INTERVENTIONAL | 01/01/2010 | Dendritic mRNA therapy |
| NCT01066390 | A Study on the Safety and Immunogenicity of Combined Intradermal and Intravenous Administration of an Autologous mRNA Electroporated Dendritic Cell Vaccine in Patients With Previously Treated Unresectable Stage III or IV Melanoma | COMPLETED | This phase I study plan is divided in the following four phases:  * Eligibility Screen Phase (week -4 to -1): Following written informed consent patients with metastatic melanoma (AJCC stage III/IV with unresectable disease) will undergo an eligibility screen (incl. blood analysis and PET/CT-scan). * TriMix-DC Vaccine Manufacturing Phase (week I to IV): eligible patients will undergo a leucapheresis (15 liter of venous blood) for the preparation of autologous TriMix-DC vaccine. Vaccine preparations will be manufactured and quality-controlled (during an interval of 4 weeks following the leucapheresis) and released for patient administration if the TriMix-DC preparation fulfills the predefined quality requirements. * TriMix-DC Vaccine Administration Phase (Week 1 to 24): 4 weeks after the leucapheresis patients will initiate therapeutic vaccination with the TriMix-DC vaccine by IV and ID administration. The vaccines will be administered at 4 different visits that will be separated with an interval of 2 weeks. At each vaccination a total of 12.106 DC per antigen will be administered. * Patients will be allocated to three different cohorts:   * The first cohort will receive 10% of TriMix-DC by iv and 90% by id injection.  * The second cohort 25% by iv and 75% by id injection.  * The third cohort 50% by iv and 50% by id injection. * During the week following the administration of the fourth vaccine (= week 8), a DTH-test and punch biopsy of the injection site will be performed as well as a second leucapheresis (for the purpose of immuno-monitoring) and tumor evaluation (by PET-CT). * A fifth vaccine will be administered and a repeat tumor staging performed in week 16 (= 8w after the fourth vaccine). * End of study visit: Patients will perform an "end of study visit" 8 weeks after the fifth vaccine (= week 24) as well as a new tumor evaluation (PET/CT). * Follow-up Phase: survival data will be obtained until 3 years after the initiation of vaccine therapy or the time of death. | NO | Melanoma | BIOLOGICAL: TriMix-DC | Bart Neyns | PHASE1 | INTERVENTIONAL | 01/12/2009 | Dendritic mRNA therapy |
| NCT01010256 | The Expression of PTEN Protein and mRNA in Malignant Cells of Chronic Myelomonocytic Leukemia | TERMINATED | The purpose of this study is to evaluate the level of a specific protein (PTEN) in the cancer cells of chronic myelomonocytic leukemia (CMML) patients. This protein might be involved in the transformation from normal blood cells to leukemia cells. The PTEN protein has not been investigated in CMML specifically but it has been discovered in closely related cancers. If this study demonstrates an abnormality in this protein, future testing will be designed to evaluate the genetic abnormality that resulted in lack of the normal presence of this protein. The goal is that the results of this study will help to develop new drugs and strategies to treat the future patients with CMML by understanding the abnormality of the disease at the cellular and molecular levels. The results of this study can also be utilized by future studies to develop individualized treatment to patients who have abnormal levels of this protein. | NO | Chronic Myelomonocytic Leukemia | | University of Arkansas | | OBSERVATIONAL | 01/11/2009 | mRNA drug |
| NCT00978913 | Transfected Dendritic Cell Based Therapy for Patients With Breast Cancer or Malignant Melanoma | COMPLETED | The primary aim of this study is to evaluate the toxicity of the vaccine and the combination of the vaccine and Cyclophosphamide, and to evaluate the immune response induced by the vaccine. The secondary aim is to investigate the clinical tumour response and duration of tumour and immune response. | NO | Breast Cancer\|Malignant Melanoma | BIOLOGICAL: DC vaccine | Inge Marie Svane | PHASE1 | INTERVENTIONAL | 01/09/2009 | Dendritic mRNA therapy |
| NCT00890032 | Vaccine Therapy in Treating Patients Undergoing Surgery for Recurrent Glioblastoma Multiforme | COMPLETED | RATIONALE: Vaccines made from a person's tumor cells and dendritic cells may help the body build an effective immune response to kill tumor cells.  PURPOSE: This phase I trial is studying the side effects of vaccine therapy in treating patients undergoing surgery for recurrent glioblastoma multiforme (GBM). | NO | Recurrent Central Nervous System Neoplasm | BIOLOGICAL: BTSC mRNA-loaded DCs | John Sampson | PHASE1 | INTERVENTIONAL | 01/09/2009 | Dendritic mRNA therapy |
| NCT00961844 | Trial for Vaccine Therapy With Dendritic Cells in Patients With Metastatic Malignant Melanoma | TERMINATED | In this trial the investigators want to combine chemotherapy with immunotherapy by giving the patients Temozolomide, before vaccination. The investigators have also included hTERT and survivin mRNA in the vaccine. Finally, the investigators want to introduce ex vivo T cell expansion after lymphodepletion for the patients who show an immune response. | NO | Metastatic Malignant Melanoma | BIOLOGICAL: Dendritic cells - transfected with hTERT-, survivin- and tumor cell derived mRNA + ex vivo T cell expansion and reinfusion\|DRUG: Temozolomide | Steinar Aamdal | PHASE1\|PHASE2 | INTERVENTIONAL | 01/08/2009 | Dendritic mRNA therapy |
| NCT01022333 | The Potential for Oral Diindolylmethane (DIM) Supplementation to Increase the Production of the BRCA1 Protein in BRCA1 Mutation Carriers | UNKNOWN | Women with a BRCA1 mutation face a lifetime risk of breast cancer of approximately 70% and a lifetime risk of ovarian cancer of approximately 40%. A number of potential anti-cancer nutrients have been proposed, however, it is important that diet supplements be evaluated prior to general recommendation.  The risk of breast and ovarian cancer in carriers of a BRCA1 mutation might be lowered by some nutritional supplements. For example, green tea, broccoli and vitamin D are of potential interest. One dietary supplement that is thought to have potential for BRCA1 carriers is diindolylmethane (DIM), which is an active ingredient in broccoli and other green vegetables. DIM - is found in vegetables like broccoli and is available as a supplement in health food stores. The investigators think that DIM may increase the production of the normal copy of BRCA1 and offset the effect of the mutation.  The purpose of this study is to determine that there is a potential for oral DIM supplementation to result in the increased production of the BRCA1 protein in BRCA1 mutation carriers. The results of the study will also serve as an evaluation of the current use and success of preventive strategies for BRCA1 mutation carriers. | NO | Breast Cancer | DIETARY_SUPPLEMENT: Diindolylmethane (DIM) | Women's College Hospital | PHASE1 | INTERVENTIONAL | 01/07/2009 | mRNA drug |
| NCT00929019 | Messenger Ribonucleic Acid (mRNA) Transfected Dendritic Cell Vaccination in High Risk Uveal Melanoma Patients | TERMINATED | 1. Rationale   Immunotherapy applying ex vivo generated and tumor antigen-loaded dendritic cells (DC) has now successfully been introduced in the clinic. A limited, but consistent, number of objective immunological and clinical responses have been observed. Most of the successful results have been observed in patients with minimal residual disease, rather than patients with advanced metastatic disease. Moreover, the investigators' preliminary results show that presence of tumor epitope specific T cells in biopsies taken from delayed type hypersensitivity (DTH) reaction sites highly correlates with prolonged progression free survival (PFS).   Within uveal melanoma patients, a group with high risk of metastatic disease can be identified on basis of tumor specific genetic changes in loss of chromosome 3.   At present no standard adjuvant or systemic treatment is available. Applying DC-based immunotherapy in this group of high risk patients might reduce the risk of recurrence without interference in the current treatment guidelines. 2. Objectives   In this joint clinical study of Radboud University Nijmegen Medical Centre (RUNMC) and Rotterdam Eye Hospital, the investigators aim to determine the in vivo immunological response induced in high risk uveal melanoma patients vaccinated with mRNA-transfected DC. 3. Study design   This study is an open label non-randomized phase II intervention study. 4. Study population   The investigators' study population consists of HLA-A2 positive patients with a high risk uveal melanoma with proven expression of melanoma associated antigens tyrosinase and/or gp100. 5. Main study endpoints  This is an exploratory study aiming to demonstrate proof of principle. The first study endpoints are in vivo immunological response induced in high risk uveal melanoma patients vaccinated with mRNA-transfected DC, administered i.v./i.d. and toxicity. Secondary study endpoints are progression free survival, overall survival, and toxicity. | NO | Uveal Melanoma | BIOLOGICAL: autologous dendritic cells electroporated with mRNA | Radboud University Medical Center | PHASE1\|PHASE2 | INTERVENTIONAL | 01/06/2009 | Dendritic mRNA therapy |
| NCT00940004 | Toll-like Receptor (TLR) Ligand Matured Dendritic Cell Vaccination in Melanoma Patients | COMPLETED | Objectives:  This is an exploratory study, consisting of two parts. In part I a dose escalation is performed and the primary objective is the safety of different doses of TLR-dendritic cell (TLR-DC). In part II TLR-DC vaccination will be compared with cytokine-matured DC vaccination and the primary objective of this part is the immunological response to TLR-DC vaccination, with toxicity and clinical efficacy being secondary objectives. These studies will provide important data on the safety and immunological effects of TLR-matured DC.  Study design:  This study is an open label prospective exploratory intervention study.  Study population:  The investigators' study population consists of HLA-A2.1 positive melanoma patients, with proven expression of melanoma associated tumor antigens gp100 and tyrosinase. Melanoma patients with regional lymph node metastasis in whom a radical lymph node dissection is planned or performed within 2 months of inclusion in this study (further referred to as stage III) and melanoma patients with measurable distant metastases (further referred to as stage IV) will be included. | NO | Melanoma | BIOLOGICAL: autologous dendritic cell vaccination | Radboud University Medical Center | PHASE1\|PHASE2 | INTERVENTIONAL | 01/06/2009 | Dendritic mRNA therapy |
| NCT00846456 | Safe Study of Dendritic Cell (DC) Based Therapy Targeting Tumor Stem Cells in Glioblastoma | COMPLETED | The study induces an immune response towards the stem-cell like part of glioblastomas in combination with standard therapy. The aim is to define and characterize the feasibility, potential adverse effects of such therapy and measure time to progression and survival. | NO | Glioblastoma\|Brain Tumor | BIOLOGICAL: Dendritic cell vaccine with mRNA from tumor stem cells | Oslo University Hospital | PHASE1\|PHASE2 | INTERVENTIONAL | 01/01/2009 | Dendritic mRNA therapy |
| NCT01153113 | Human Telomerase Reverse Transcriptase Messenger RNA (hTERT mRNA) Transfected Dendritic Cell Vaccines | WITHDRAWN | The purpose of this research is to develop a new and powerful type of immune therapy for prostate cancer patients. This therapy involves vaccinations with special stimulator cells found in the human body called dendritic cells. These dendritic cells can take up proteins released from cancer cells and present pieces of these proteins to immune cells called T lymphocytes to create a strong stimulatory signal to fight the cancer.  One of these proteins is called telomerase, which is found on prostate cancers and is critically important for prostate cancer cells to grow. However, in most cancer patients, the immune system does not adequately destroy the tumor because the T cells are not stimulated sufficiently. T cells require strong stimulation before they grow and become active against cancer cells.  We have discovered that substances called ribonucleic acids (RNA), which carry the genetic instructions for the production of telomerase, can be used to overcome this problem and stimulate a strong immune response in cancer patients.  In order to test this hypothesis we have designed a clinical study and will enroll patients with metastatic prostate cancer expressing telomerase in order to determine whether or not this vaccine will stimulate T cells, which can recognize and kill prostate tumor cells.  The main objectives of this study are to find out whether injections with dendritic cells grown from blood cells and "pulsed" (mixed together for a short period of time) with RNA derived from the patient's own tumor are:  1. Safe without inducing any major side effects. 2. And effective in boosting the patient body's immunity against telomerase expressing prostate cancer cells. 3. Finally, we will test whether or not tumor shrinkage based on serum PSA levels or on X-ray studies will occur.  We hope that this new form of immune therapy, although in its infancy, will ultimately slow down tumor growth and prolong survival of prostate cancer patients. | NO | Metastatic Prostate Cancer | BIOLOGICAL: hTERT mRNA DC\|BIOLOGICAL: hTERT mRNA DC | University of Florida | PHASE1\|PHASE2 | INTERVENTIONAL | 01/01/2008 | Dendritic mRNA therapy |
| NCT00514189 | Feasibility Study of Acute Myelogenous Leukemia mRNA Plus Lysate Loaded Dendritic Cell Vaccines | TERMINATED | Primary Objectives:  1. To determine the feasibility of delivering autologous dendritic cells (DCs) loaded with acute myelogenous leukemia (AML) lysate plus messenger RNA (mRNA) to AML patients following consolidation therapy. 2. To determine the toxicity of autologous DCs loaded with AML lysate plus mRNA. 3. To quantitate immune responses in patients who receive autologous DCs loaded with AML lysate plus mRNA.  Secondary Objectives:  1. To evaluate minimal residual disease following DC therapy using the polymerase chain reaction assay for the Wilm's Tumor-1 gene. 2. To asses the disease-free and overall survival of AML patients who receive the autologous DCs loaded with AML lysate plus mRNA. | NO | Leukemia | BIOLOGICAL: Autologous Dendritic Cells | M.D. Anderson Cancer Center | PHASE1 | INTERVENTIONAL | 01/07/2007 | Dendritic mRNA therapy |
| NCT00510133 | A Study of Active Immunotherapy With GRNVAC1 in Patients With Acute Myelogenous Leukemia (AML) | COMPLETED | This is a phase II study to evaluate the safety, feasibility and efficacy of immunotherapy with GRNVAC1 in patients with AML. | NO | Acute Myelogenous Leukemia | BIOLOGICAL: GRNVAC1 | Asterias Biotherapeutics, Inc. | PHASE2 | INTERVENTIONAL | 01/07/2007 | mRNA drug |
| NCT00626483 | Basiliximab in Treating Patients With Newly Diagnosed Glioblastoma Multiforme Undergoing Targeted Immunotherapy and Temozolomide-Caused Lymphopenia | COMPLETED | RATIONALE: Monoclonal antibodies, such as basiliximab, can block tumor growth in different ways. Some block the ability of tumor cells to grow and spread. Others find tumor cells and help kill them or carry tumor-killing substances to them. Drugs used in chemotherapy, such as temozolomide, work in different ways to stop the growth of tumor cells, either by killing the cells or by stopping them from dividing. Radiation therapy uses high-energy x-rays to kill tumor cells. Vaccines may help the body build an effective immune response to kill tumor cells. Giving these treatments together may kill more tumor cells. Granulocyte Macrophage-Colony Stimulating Factor (GM-CSF) is a powerful adjuvant capable of stimulating macrophage function, inducing proliferation and maturation of DCs, and is able to enhance T-lymphocyte stimulatory function. Intradermal administration of GM-CSF enhances the immunization efficacy at the site of administration  PURPOSE: This clinical trial is studying how well basiliximab works in treating patients with newly diagnosed glioblastoma multiforme and temozolomide-caused lymphopenia who are undergoing targeted immunotherapy. | NO | Malignant Neoplasms Brain | BIOLOGICAL: RNA-loaded dendritic cell vaccine\|DRUG: basiliximab | Gary Archer Ph.D. | PHASE1 | INTERVENTIONAL | 24/04/2007 | Dendritic mRNA therapy |
| NCT00204516 | Vaccination With Tumor mRNA in Metastatic Melanoma - Fixed Combination Versus Individual Selection of Targeted Antigens | COMPLETED | The purpose of the vaccination protocol is to induce specific immune responses against melanoma associated antigens by intradermal injections of mRNA coding for the corresponding antigen. | NO | Malignant Melanoma | BIOLOGICAL: mRNA coding for melanoma associated antigens\|DRUG: GM-CSF | University Hospital Tuebingen | PHASE1\|PHASE2 | INTERVENTIONAL | 01/04/2007 | neoantigen mRNA treatment |
| NCT00204594 | Local Treatment of Metastatic Melanoma With Autologous Lymphocytes and the Bispecific Antibody rM28 | COMPLETED | Phase I/II clinical trial to analyze safety and efficiency of intralesional application of the bispecific single chain antibody rM28 and autologous PBMCs in patients with metastatic melanoma stage III/IV and unresectable metastasis. | NO | Malignant Melanoma | DRUG: rM28\|DRUG: autologous PBMCs | University Hospital Tuebingen | PHASE1\|PHASE2 | INTERVENTIONAL | 01/10/2005 | mRNA drug |
| NCT00315653 | Survivin Urine mRNA Assay Risk of Bladder Cancer Study | COMPLETED | The purpose of this clinical trial is to evaluate the ability of urinary Survivin mRNA measurement to estimate the risk of bladder cancer at the time of cystoscopy in subjects with no prior history of bladder cancer presenting with microscopic or macroscopic hematuria. | NO | Bladder Cancer | | Fujirebio Diagnostics, Inc. | | OBSERVATIONAL | 01/09/2005 | mRNA drug |
| NCT00449137 | Arsenic Trioxide, Fluorouracil, and Leucovorin in Treating Patients With Stage IV Colorectal Cancer That Has Relapsed or Not Responded to Treatment | COMPLETED | RATIONALE: Drugs used in chemotherapy, such as fluorouracil and leucovorin, work in different ways to stop the growth of tumor cells, either by killing the cells or by stopping them from dividing. Arsenic trioxide may help fluorouracil and leucovorin work better by making tumor cells more sensitive to the drugs. Giving arsenic trioxide together with fluorouracil and leucovorin may kill more tumor cells.  PURPOSE: This phase I trial is studying the side effects and best dose of arsenic trioxide and fluorouracil when given together with leucovorin in treating patients with stage IV colorectal cancer that has relapsed or not responded to treatment. | NO | Colorectal Cancer | DRUG: Arsenic trioxide\|DRUG: Fluorouracil\|DRUG: Leucovorin calcium\|OTHER: Plasma levels of elemental arsenic\|GENETIC: Peripheral Blood Mononuclear Cells (PBMC) for mRNA analysis\|PROCEDURE: Tumor Biopsy (Fine-Needle Aspiration) | University of Miami | PHASE1 | INTERVENTIONAL | 01/06/2005 | mRNA drug |
| NCT00285103 | SPC2996 in Chronic Lymphocytic Leukaemia | COMPLETED | The purpose of this study is to determine whether SPC2996 is effective and safe in the treatment of Chronic Lymphocytic Leukaemia (CLL) | NO | Chronic Lymphocytic Leukemia | DRUG: SPC2996 | Santaris Pharma A/S | PHASE1\|PHASE2 | INTERVENTIONAL | 01/06/2005 | mRNA drug |
| NCT00834002 | Dendritic Cell Vaccination for Patients With Acute Myeloid Leukemia in Remission | COMPLETED | RATIONALE: Vaccines made from a patient's white blood cells (dendritic cells) and a specific leukemia antigen (Wilms tumor antigen-1) may induce an effective immune response to kill residual leukemic cells and/or prevent leukemia relapse.  PURPOSE: This phase I/II trial is studying the feasibility, safety and efficacy of intradermal mRNA-transfected dendritic cell vaccination therapy in patients with acute myeloid leukemia. | NO | Acute Myeloid Leukemia (AML) | BIOLOGICAL: injection of antigen-loaded cultured dendritic cells | University Hospital, Antwerp | PHASE1 | INTERVENTIONAL | 01/03/2005 | Dendritic mRNA therapy |
| NCT00204607 | Intradermal Vaccination With Stabilized Tumor mRNA - a Clinical Phase I/II Trial in Melanoma Patients | COMPLETED | vaccination protocol to induce specific immune responses against melanoma associated antigens by intradermal injections of mRNA coding for the corresponding antigen | NO | Malignant Melanoma | BIOLOGICAL: mRNA\|DRUG: GM-CSF s.c. | University Hospital Tuebingen | PHASE1\|PHASE2 | INTERVENTIONAL | 01/07/2004 | neoantigen mRNA treatment |
| NCT00243529 | Peptide-pulsed vs. RNA-transfected Dendritic Cell Vaccines in Melanoma Patients | COMPLETED | Dendritic cells (DCs)are the most potent antigen-presenting cells of the immune system, as such they are able to direct the immune system specifically against cancer cells. Currently DCs are used in clinical vaccination studies and immunological and clinical responses have been observed. For inducing anti-tumor immunity, the DCs have to be loaded with tumor antigen (i.e. molecular structures that are presented by the tumor, that are recognized by the immune system). Currently most studies use tumor peptides (small protein fragments) for this purpose. This approach has several disadvantages: only patients with a certain HLA-type can be treated and the immune response that is induced by the vaccine is limited to the used peptides. These disadvantages do not exist when the DCs present antigen which is endogenously processed, for example after RNA transfection. For this reason we investigate the immunogenicity of DCs that are pulsed with peptides or transfected with mRNA encoding melanoma associated antigens in stage III and IV melanoma patients. | NO | Melanoma Stage III or IV | BIOLOGICAL: autologous dendritic cell vaccine | Radboud University Medical Center | PHASE1\|PHASE2 | INTERVENTIONAL | 01/04/2004 | Dendritic mRNA therapy |
| NCT00228189 | Carcinoembryonic Antigen-loaded Dendritic Cells in Advanced Colorectal Cancer Patients | COMPLETED | Dendritic cells (DCs) are the professional antigen-presenting cells of the immune system. As such they are currently used in clinical vaccination protocols in cancer patients. We evaluate the ability of mature DCs pulsed with carcinoembryonic antigen (CEA)-peptide (arm A) or electroporated with CEA-mRNA (arm B) to induce CEA-specific T cell responses in patients with resectable liver metastases from colorectal cancer. To evaluate immune responses, CEA-specific T cell reactivity is monitored in peripheral blood, resected abdominal lymph nodes, tumor tissue and biopsies of vaccination sites and post-treatment DTH skin tests. Patients are vaccinated intradermally and intravenously with CEA-peptide pulsed mature DCs three times prior to resection of liver metastases. In 2007 a side-study has been added (arm C), in which patients with stage III or high-risk stage II colorectal cancer that are amenable for standard adjuvant oxaliplatin/capecitabine therapy are vaccinated with CEApeptide-pulsed DCs. Also in this group, safety and immune responses in peripheral blood and the DTH-skin test are the primary endpoints. Results are compared with the results obtained in arm A. | NO | Colorectal Cancer\|Liver Metastases | BIOLOGICAL: CEA-loaded dendritic cell vaccine | Radboud University Medical Center | PHASE1\|PHASE2 | INTERVENTIONAL | 01/12/2003 | Dendritic mRNA therapy |
| NCT01278940 | Trial of Vaccine Therapy With mRNA- Transfected Dendritic Cells in Patients With Advanced Malignant Melanoma | COMPLETED | PRIMARY OBJECTIVES: Determination of safety and toxicity of vaccination with patients' tumour mRNA transfected DCs .  SECONDARY OBJECTIVES:Determine immunological response to the vaccine (induction of specific T-cell response) and assessment of tumour response | NO | Malignant Melanoma | BIOLOGICAL: Dendritic Cells (DC) malignant melanoma\|PROCEDURE: IL-2 | Oslo University Hospital | PHASE1\|PHASE2 | INTERVENTIONAL | 01/03/2002 | Dendritic mRNA therapy |
| NCT01278914 | Trial of Vaccine Therapy With mRNA- Transfected Dendritic Cells in Patients With Androgen Resistant Metastatic Prostate Cancer | COMPLETED | PRIMARY OBJECTIVES:  Determination of safety and toxicity of vaccination with mRNA transfected DC (based on blood samples, and adverse events).  SECONDARY OBJECTIVES:  Determine immunological response to the vaccine (induction of specific T-cell response) and assessment of tumour response. | NO | Prostate Cancer | BIOLOGICAL: Dendritic Cells (DC) prostate | Oslo University Hospital | PHASE1\|PHASE2 | INTERVENTIONAL | 01/02/2002 | Dendritic mRNA therapy |
| NCT02449993 | Re-Examination of Tumor Material and Re-Evaluation of Patient Data From Patients Treated With Neo-adjuvant Therapy | COMPLETED | This is a prospective examination of ribonucleic acid (RNA) extracted from tumor material of breast cancer patients treated with a neo-adjuvant therapy. The RNA will be analysed for expression of estrogen receptor (ER 1), progesterone receptor (PgR), HER2 and Ki-67 with MammaTyper  According to the determined values for the individual parameters at least 4 subtypes can be distinguished to date  * Luminal A-type * Luminal B-type * HER2-type * Triple-negative-type As non-clinical endpoint, the agreement of new subtyping with Immunohistochemical methods will be evaluated.  As clinical objective, the 5 year Distant metastasis free survival (DMFS) and Overall survival (OS) will be reevaluated according to the new subtyping. | NO | Breast Cancer | DEVICE: MammaTyperBioNTech Diagnostics GmbH | | OBSERVATIONAL | Jun-14 |  | mRNA drug |
| NCT05978102 | A Dose Escalation and Dose Expansion Clinical Study of STI-7349 in Subjects With Advanced Solid Tumors | RECRUITING | This is a first-in-human, Phase open-label, 2-period dose escalation and expansion study of STI-7349 administered intravenously to subjects with advanced solid tumors:  * Period I is divided into two parts: Dose escalation for STI-7349 alone (1A) and dose expansion for STI-7349 alone (1B). In Part 1A, a rapid titration approach and traditional 3 + 3 trial design will be used to assess the safety, dose-limiting toxicities (DLTs), maximum tolerated dose (MTD), PK/biomarker profile, and to determine the recommended Phase 2 dose (RP2D) of STI-7349 alone; in Part 1B, an expansion study of STI-7349 alone will be conducted in target tumor types that may potentially benefit to assess the safety and preliminary efficacy of STI-7349 alone. * Period is divided into two parts: Dose escalation for STI-7349 in combination with Pembrolizumab (2A) and dose expansion for STI-7349 in combination with Pembrolizumab (2B). In Part 2A, a dose escalation study of STI-7349 in combination with Pembrolizumab is planned to be conducted using RP2D of STI-7349 alone as the starting dose, which will use a traditional 3 + 3 trial design to assess the safety, DLTs, MTD, PK/biomarker profile of STI-7349 in combination with Pembrolizumab, and to determine the RP2D of STI-7349 in combination with Pembrolizumab; in Part 2B, an expansion study of STI-7349 in combination with Pembrolizumab or add standard treatment on the basis of STI-7349 combined with pembrolizumab will be conducted in target tumor types that may potentially benefit to assess the safety and preliminary efficacy of the combination. | NO | Advanced Solid Tumor | DRUG: STI-7349\|DRUG: Pembrolizumab\|DRUG: Standard treatmentoCThe Fourth Affiliated Hospital of Zhejiang University School of Medicine | PHASE1\|PHASE2 | INTERVENTIONAL | 23/08/2023 |  | mRNA drug |

Data from ClinicalTrials.gov

Table S3. Summary of FDA approved combination therapies for cancer (January 23, 2023 - March 24, 2024)

| **Date** | **Tumor type** | **Combination agent** | **combination** |
| --- | --- | --- | --- |
| 01/23/2013 | mCRC has progressed on a first-line bevacizumab-containing regimen | bevacizumab+fluoropyrimidine-irinotecan or fluoropyrimidine-oxaliplatin based chemotherapy | Anti-angiogenesis+chenmotherapy |
| 09/06/2013 | metastatic adenocarcinoma of the pancreas | pertuzumab+gemcitabine | monoclonal antibody drug+chemotherapy |
| 01/10/2014 | unresectable or metastatic melanoma with a BRAF V600E or V600K mutation | trametinib+dabrafenib | small molecular inhibitor+small molecular inhibtor |
| 07/23/2014 | CLL | idelalisib+rituximab | small molecular inhibitor+monoclonal antibody drug |
| 08/14/2014 | persistent, recurrent or metastatic cervical cancer | bevacizumab+paclitaxel and cisplatin or paclitaxel and topotecan | Anti-angiogenesis+chenmotherapy |
| 11/05/2014 | GEJ | ramucirumab+paclitaxel | Anti-angiogenesis+chenmotherapy |
| 11/14/2014 | Platinum-resistant, recurrent EOC/FFT/PPC | bevacizumab+paclitaxel, pegylated liposomal doxorubicin, or topotecan | Anti-angiogenesis+chenmotherapy |
| 12/12/2014 | NSCLC with disease progression on or after platinum-based chemotherapy | ramucirumab+docetaxel | Anti-angiogenesis+chenmotherapy |
| 03/10/2015 | Pediatric HRNB pts with PR to prev first-line MMMT | dinutuximab+GM-CSF，IL-2，RA | monoclonal antibody drug+cytokine |
| 04/24/2015 | mCRC has progressed on a first line bevacizumab-, oxaliplatin- and fluoropyrimidine-containing regimen | ramucirumab+FOLFIRI | Anti-angiogenesis+chenmotherapy |
| 09/30/2015 | BRAF V600 wild-type, unresectable or metastatic melanoma | nivolumab+ipilimumab | ICIs+ICIs |
| 10/01/2015 | BRAFWT metastatic melanoma | Nivolumab + Ipilimumab | ICIs+ICIs |
| 11/10/2015 | unresectable or metastatic melanoma with BRAF V600E or V600K mutations | cobimetinib+vemurafenib | small molecular inhibitor+small molecular inhibtor |
| 11/20/2015 | unresectable or metastatic melanoma with BRAF V600E or V600K mutations | trametinib+dabrafenib | small molecular inhibitor+small molecular inhibtor |
| 11/24/2015 | NSCLC | necitumumab+gemcitabine and cisplatin | monoclonal antibody drug+chemotherapy |
| 01/23/2016 | Metastatic melanoma across BRAF status | Nivolumab + Ipilimumab | ICIs+ICIs |
| 02/19/2016 | HR+/HER2- ABC/MBC post-endocrine therapy progression | palbociclib+fulvestrant | small molecular inhibitor+small molecular inhibtor |
| 02/26/2016 | FL | obinutuzumab+bendamustine | monoclonal antibody drug+chemotherapy |
| 05/13/2016 | RCC | lenvatinib+everolimus | small molecular inhibitor+small molecular inhibtor |
| 05/10/2017 | First-line metastatic NSCLC | Pembrolizumab + Pemetrexed + Carboplatin | ICIs+small molecular inhibitor+chemotherapy |
| 06/22/2017 | follicular lymphoma, diffuse large B-cell lymphoma, and chronic lymphocytic leukemia | dabrafenib+trametinib | small molecular inibitor+small molecular inhibitor |
| 09/28/2017 | HER2-negative advanced or metastatic breast cancer with disease progression following endocrine therapy | abemaciclib+fulvestrant | small molecular inibitor+small molecular inhibitor |
| 11/16/2017 | FL | obinutuzumab+chemotherapy followed by obinutuzumab | monoclonal antibody drug+chemotherapy |
| 12/20/2017 | HER2-positive early breast cancer at high risk of recurrence | pertuzumab+trastuzumab+chemotherapy | monoclonal antibody drug+chemotherapy |
| 02/16/2018 | Stage III NSCLC | Durvalumab + Chemoradiation | ICIs+chemotherapy |
| 02/26/2018 | postmenopausal women with hormone receptor (HR)-positive, human epidermal growth factor receptor 2 (HER2)-negative advanced or metastatic breast cancer | abemaciclib+an aromatase inhibitor | small molecular inhibitor+small molecular inhibtor |
| 04/16/2018 | First-line intermediate or poor-risk advanced RCC | Nivolumab + Ipilimumab | ICIs+ICIs |
| 04/30/2018 | melanoma with BRAF V600E or V600K mutations | dabrafenib+trametinib | small molecular inhibitor+small molecular inhibtor |
| 06/13/2018 | epithelial ovarian, fallopian tube, or primary peritoneal cancer | bevacizumab+carboplatin+paclitaxel | ICIs+chemotherapy |
| 06/27/2018 | unresectable or metastatic melanoma with a BRAF V600E or V600K mutation | encorafenib+binimetinib | small molecular inhibitor+small molecular inhibtor |
| 07/10/2018 | MSI-H or dMMR metastatic CRC | Nivolumab + Ipilimumab | ICIs+ICIs |
| 07/18/2018 | pre/perimenopausal women with HR-positive, HER2-negative advanced or metastatic breast cance | ribociclib+an aromatase inhibitor | small molecular inhibitor+small molecular inhibtor |
| 08/20/2018 | Metastatic nonsquamous NSCLC | Pembrolizumab + Pemetrexed + Chemotherapy | ICIs+small molecular inhibitor+chemotherapy |
| 10/30/2018 | First-line metastatic squamous NSCLC | Pembrolizumab + Chemotherapy | ICIs+chemotherapy |
| 11/16/2017 | previously untreated systemic anaplastic large cell lymphoma or other CD30-expressing peripheral T-cell lymphomas | brentuximab vedotin+chemotherapy | monoclonal antibody drug+chemotherapy |
| 11/21/2017 | AML patients who are 75 years old or older | glasdegib+low-dose cytarabine (LDAC) | small molecular inhibitor+chemotherapy |
| 11/21/2018 | AML | venetoclax+azacitidine, decitabine, or low-dose cytarabine (LDAC) | small molecular inihitor+chemotherapy |
| 12/06/2018 | First-line NSCLC | Atezolizumab + bevacizumab, paclitaxel and carboplatin | ICIs+anti-small molecular inhibitor/Anti-angiogenesis+chemotherapy |
| 03/08/2019 | Metastatic TNBC | Atezolizumab + Nabpaclitaxel | ICIs+chemotherapy |
| 03/18/2019 | Extensive-stage SCLC | Atezolizumab + Carboplatin + Etoposide | ICIs+chemotherapy |
| 04/19/2019 | First-line advanced RCC | Pembrolizumab + Axitinib | ICIs+anti-anginogenesis+chemotherapy |
| 05/14/2019 | First-line advanced RCC | Avelumab + Axitinib | ICIs+small molecular inhibitor |
| 05/24/2019 | (HER2)-negative, PIK3CA-mutated, advanced or metastatic breast cance | alpelisib+fulvestrant | small molecular inhibitor+small molecular inhibtor |
| 05/28/2019 | MZL | lenalidomide+rituximab | monoclonal antibody drug+immunomodulator |
| 06/10/2019 | DLBCL | vedotin-piiq+bendamustine | ICIs+chemotherapy |
| 06/11/2019 | HNSCC | Pembrolizumab + Chemotherapy | ICIs+chemotherapy |
| 06/17/2019 | Metastatic SCLC | Pembrolizumab + Chemotherapy | ICIs+chemotherapy |
| 09/17/2019 | Advanced endometrial carcinoma | Pembrolizumab + Lenvatinib | ICIs+small molecular inhibitor+chemotherapy |
| 12/03/2019 | First-line Metastatic NSCLC without EGFR/ALK aberrations | Atezolizumab + Nab-paclitaxel + Carboplatin | ICIs+chemotherapy |
| 3/2/2020 | multiple myeloma | isatuximab-irfc+pomalidomide | monoclonal antibody drug+immunomodulator |
| 3/10/2020 | hepatocellular carcinoma | nivolumab+ipilimumab | ICIs+ICIs |
| 2/25/2020 | HER3-positive breast cancer | neratinib+capecitabine | small molecular inhibitor/Anti-angiogenesis+small molecular inhibtor |
| 3/30/2020 | small cell lung cancer | durvalumab+etoposide and either carboplatin or cisplatin | ICIs+chemotherapy |
| 4/8/2020 | metastatic colorectal cancer with a BRAF V601E mutation | encorafenib+cetuximab | monoclonal antibody drug+small molecular inhibitor |
|  |  |  |  |
| 4/17/2020 | HER3-positive metastatic breast cancer | tucatinib+trastuzumab+capecitabine | monoclonal antibody drug/Anti-angiogenesis+small molecular inhibitor |
| 4/21/2020 | chronic lymphocytic leukemia | ibrutinib+rituximab | monoclonal antibody drug+small molecular inhibitor |
| 5/8/2020 | ovarian, fallopian tube, or primary peritoneal cancers | olaparib+bevacizumab | small molecular inhibitor+Anti-angiogenesis |
| 5/15/2020 | first-line mNSCLC (PD-L1 tumor expression≥2%) | nivolumab+ipilimumab | ICIs+ICIs |
| 5/25/2022 | newly diagnosed acute myeloid leukemia | ivosidenib+azacitidine | small molecular inhibitor+small molecular inhibtor |
| 5/26/2020 | first-line treatment of metastatic NSCLC | nivolumab+ipilimumab+chemotherapy | ICIs+ICIs+chemotherapy |
| 5/29/2020 | first-line metastatic NSCLC | ramucirumab+erlotinib | small molecular inhibitor+anti-angiogenesis |
| 5/29/2020 | unresectable hepatocellular carcinoma | atezolizumab+bevacizumab | ICIs+Anti-angiogenesis |
| 5/29/2020 | first-line metastatic NSCLC | ramucirumab+erlotinib | monoclonal antibody drug+small molecular inhibitor |
| 6/29/2020 | HER3-positive breast cancer | pertuzumab+trastuzumab+hyaluronidase-zzxf | monoclonal antibody drug+monoclonal antibody drug |
| 7/7/2020 | myelodysplastic syndromes | decitabine+cedazuridine | small molecular inhibitor+small molecular inhibtor |
| 7/30/2020 | BRAF V601 unresectable or metastatic melanoma | atezolizumab+cobimetinib | ICIs+small molecular inhibitor |
| 7/31/2020 | DLBCL | tafasitamab-cxix+lenalidomide | monoclonal antibody drug+immunomodulator |
| 8/20/2020 | multiple myeloma | carfilzomib+daratumumab | small molecular inhibitor+monoclonal antibody drug |
| 9/8/2020 | breast cancer | atezolizumab+paclitaxel | ICIs+chemotherapy |
| 10/2/2020 | unresectable malignant pleural mesothelioma | nivolumab+ipilimumab | ICIs+ICIs |
| 10/16/2020 | AML in adults 75 years or older | venetoclax+azacitidine, decitabine, or low-dose cytarabine (LDAC) | small molecular inhibitor+chemotherapy |
| 11/25/2020 | TNBC whose tumors express PD-L1 (CPS ≥10) | pembrolizumab+chemotherapy | ICIs+chemotherapy |
| 12/16/2020 | metastatic HER2-positive breast cancer who have received two or more prior anti-HER2 regimens | margetuximab-cmkb+chemotherapy | monoclonal antibody drug+chemotherapy |
| 1/22/2021 | advanced renal cell carcinoma | nivolumab+cabozantinib | ICIs+small molecular inhibitor |
| 4/16/2021 | metastatic gastric cancer and esophageal adenocarcinoma | nivolumab+chemotherapy | ICIs+chemotherapy |
| 7/21/2021 | advanced endometrial carcinoma | pembrolizumab+lenvatinib | ICIs+small molecular inhibitor+chemotherapy |
| 8/10/2021 | advanced renal cell carcinoma | lenvatinib+pembrolizumab | ICIs+small molecular inhibitor/Anti-angiogenesis+chemotherapy |
| 12/2/2021 | pediatric cancer indications | rituximab+chemotherapy | monoclonal antibody drug+chemotherapy |
| 5/27/2022 | first-line esophageal squamous cell carcinoma indications | Opdivo+chemotherapy | ICIs+chemotherapy |
| 6/22/2022 | unresectable or metastatic solid tumors with BRAF V601E mutation | dabrafenib+trametinib | small molecular inhibitor+small molecular inhibtor |
| 10/21/2022 | unresectable hepatocellular carcinoma | tremelimumab+durvalumab | ICIs+ICIs |
| 11/8/2022 | non-small cell lung cancer | cemiplimab-rwlc+platinum-based chemotherapy | ICIs+chemotherapy |
| 11/10/2022 | metastatic non-small cell lung cancer | durvalumab+platinum-based chemotherapy | ICIs+chemotherapy |
| 11/10/2022 | pediatric patients with classical Hodgkin lymphoma | brentuximab vedotin+chemotherapy | monoclonal antibody drug+chemotherapy |
| 1/19/2023 | colorectal cancer | tucatinib+trastuzumab | monoclonal antibody drug+small molecular inhibitor |
| 6/20/2023 | HRR gene-mutated metastatic castration-resistant prostate cancer | talazoparib+enzalutamide | small molecular inhibitor+small molecular inhibtor |
| 8/11/2023 | BRCA-mutated metastatic castration-resistant prostate cancer | niraparib+abiraterone acetate+prednisone | small molecular inhibitor+small molecular inhibtor |
| 12/15/2023 | locally advanced or metastatic urothelial cancer | enfortumab vedotin-ejfv+pembrol | ICIs+Anti-angiogenesis |
| 3/7/2024 | unresectable or metastatic urothelial carcinoma | nivolumab+cisplatin and gemcitabine | ICIs+chemotherapy |
|  | | | |
| Note: Dates are listed as month/day/year. Source: https://www.fda.gov/drugs/resources-information-approved-drugs/oncology-cancer-hematologic-malignancies-approval-notifications. | | | |
| mCRC: Metastatic Colorectal Cancer, EOC/FFT/PPC: Epithelial Ovarian Cancer/Fallopian Tube Cancer/Primary Peritoneal Cancer, NSCLC: Non-Small Cell Lung Cancer, CLL: Chronic Lymphocytic Leukemia, GEJ: Gastroesophageal Junction Cancer, RCC: Renal Cell Carcinoma, FL: Follicular Lymphoma, HER2: Human Epidermal Growth Factor Receptor 2-positive Breast Cancer, ABC/MBC: Advanced Breast Cancer/Metastatic Breast Cancer, HRNB: High-Risk Neuroblastoma, MMMT: Multimodality Multiagent Therapy (not specific to a single cancer type), BRAF: B-Raf Proto-Oncogene Mutation-positive Cancer (e.g., Melanoma), AML: Acute Myeloid Leukemia, DLBCL: Diffuse Large B-Cell Lymphoma, MZL: Marginal Zone Lymphoma, TNBC: Triple-Negative Breast Cancer, SCLC: Small Cell Lung Cancer, HNSCC: Head and Neck Squamous Cell Carcinoma, CRC: Colorectal Cancer, HCC: Hepatocellular Carcinoma, MSI-H: Microsatellite Instability-High Cancer, dMMR: Deficient Mismatch Repair Cancer, HRR: Homologous Recombination Repair gene-mutated Cancer (e.g., Prostate Cancer), CRPC: Castration-Resistant Prostate Cancer. | | | |
